# Recruited atypical Ly6G^+^ macrophages license alveolar regeneration after lung injury

**DOI:** 10.1126/sciimmunol.ado1227

**Published:** 2024-08-02

**Authors:** C. Ruscitti, J. Abinet, P. Maréchal, M. Meunier, C. de meeûs, D. Vanneste, P. Janssen, M. Dourcy, M. Thiry, F. Bureau, C. Schneider, B. Machiels, A. Hidalgo, F. Ginhoux, B.G. Dewals, J. Guiot, F. Schleich, M-M. Garigliany, A. Bellahcène, C. Radermecker, T. Marichal

**Affiliations:** 1 Laboratory of Immunophysiology, GIGA Institute, University of Liège; Liège, Belgium; 2 Faculty of Veterinary Medicine, University of Liège; Liège, Belgium; 3 Department of Pathology, FARAH Institute, University of Liège; Liège, Belgium; 4 Laboratory of Immunology-Vaccinology, FARAH Institute, University of Liège; Liège, Belgium; 5 Laboratory of Cellular and Tissular Biology, GIGA Institute, University of Liège; Liège, Belgium; 6 Laboratory of Cellular and Molecular Immunology, GIGA Institute, University of Liège; Liège, Belgium; 7 Institute of Physiology, University of Zurich; Zurich, Switzerland; 8 Area of Cell & Developmental Biology, Centro Nacional de Investigaciones Cardiovasculares Carlos III; Madrid, Spain; 9 Vascular Biology and Therapeutics Program and Department of Immunobiology, Yale University School of Medicine; New Haven, CT, USA; 10 Shanghai Institute of Immunology, Shanghai JiaoTong University School of Medicine; Shanghai, China; 11 Inserm U1015, Gustave Roussy, Bâtiment de Médecine Moléculaire ; Villejuif, France; 12 Singapore Immunology Network (SIgN), Agency for Science, Technology and Research (A*STAR); Singapore, Singapore; 13 Translational Immunology Institute, SingHealth Duke-NUS Academic Medical Centre; Singapore, Singapore; 14 Laboratory of Pneumology, GIGA Institute, University of Liège; Liège, Belgium; 15 Department of Respiratory Medicine, CHU University Hospital; Liège, Belgium; 16 Metastasis Research Laboratory, GIGA Institute, University of Liège; Liège, Belgium; 17 Walloon Excellence in Life Sciences and Biotechnology (WELBIO) Department, WEL Research Institute; Wavre, Belgium

## Abstract

The lung is constantly exposed to airborne pathogens and particles that can cause alveolar damage. Hence, appropriate repair responses are essential for gas exchanges and life. Here, we deciphered the spatiotemporal trajectory and function of an atypical population of macrophages after lung injury. Post-influenza A virus (IAV) infection, short-lived monocyte-derived Ly6G-expressing macrophages (Ly6G^+^ Macs) were recruited to the alveoli of lung perilesional areas. Ly6G^+^ Macs engulfed immune cells, exhibited a high metabolic potential and clustered with alveolar type 2 (AT2) epithelial cells in zones of active epithelial regeneration. Ly6G^+^ Macs were partially dependent on GM-CSF and IL-4 receptor signaling and were essential for AT2-dependent alveolar regeneration. Similar macrophages were recruited in other models of injury and in the airspaces of lungs from patients suspected of pneumonia. This study identifies perilesional alveolar Ly6G^+^ Macs as a spatially-restricted, short-lived macrophage subset promoting epithelial regeneration post-injury, thus representing an attractive therapeutic target for treating lung damage.

## Introduction

Severe respiratory viral infections represent a global health issue and a major threat for the healthcare systems as they often require hospitalization, such as seen during annual influenza A virus (IAV) outbreaks or the Covid-19 pandemic. Acute lung infectious episodes are typically associated with excessive lung inflammation, damage, and abnormal tissue repair that can lead to acute respiratory distress syndrome (ARDS), pneumonia and death (1–5). Deciphering the mechanisms eliciting appropriate lung regeneration and host recovery after viral-triggered lung injury is urgently needed to improve clinical management and broaden therapeutic opportunities.

Blood monocytes are heavily recruited to the lungs during the acute inflammatory phase post-infection, thereby contributing to host innate defense mechanisms. When not appropriately regulated, they are also thought to contribute to uncontrolled inflammation via the aberrant release of cytokines, which, in its extreme form, is known as the “cytokine storm”, well described in severe Covid-19 patients (3, 6–9). Recruited monocytes can also differentiate into monocyte-derived macrophages (Mo-Macs) that are either short-lived or can establish long-term residency in particular niches and can have functional consequences for lung immunity (10–15). The idea that recruited Mo-Macs are considered pathogenic after influenza infection (15, 16) or Covid-19 (6, 17), while lung-resident alveolar macrophages (AM) and interstitial macrophages (IM) exert beneficial roles (13, 18–24) is likely oversimplistic. Indeed, recruited Mo-Macs are increasingly recognized as heterogenous and can adopt distinct functional identities that depend on their differentiation trajectory, the diseased tissue microenvironment, the extent and phase of inflammation, and their activation state (9, 13, 14, 18, 25). In this regard, the fate and functions of short-lived Mo-Macs after lung injury remain incompletely described.

Here, we used an *in vivo* model of IAV-triggered injury to investigate the spatiotemporal trajectory and function of an atypical population of Mo-Macs expressing Ly6G, a marker considered to be restricted to granulocytes. We found that Ly6G^+^ Macs emerged transiently from Ccr2-dependent monocytes during the early recovery phase post-IAV, populated the alveolar lumen of lung perilesional areas and could promote progenitor AT2 differentiation and alveolar re-epithelization. Our study thus unravels the fate and function of a previously undescribed Ly6G^+^ Mac population that engages in crosstalk with epithelial cells to promote epithelial repair after viral-triggered lung injury.

## Results

### Lung Ly6G^+^ Macs emerge in the early recovery phase post-IAV infection

**Fig. 1:**
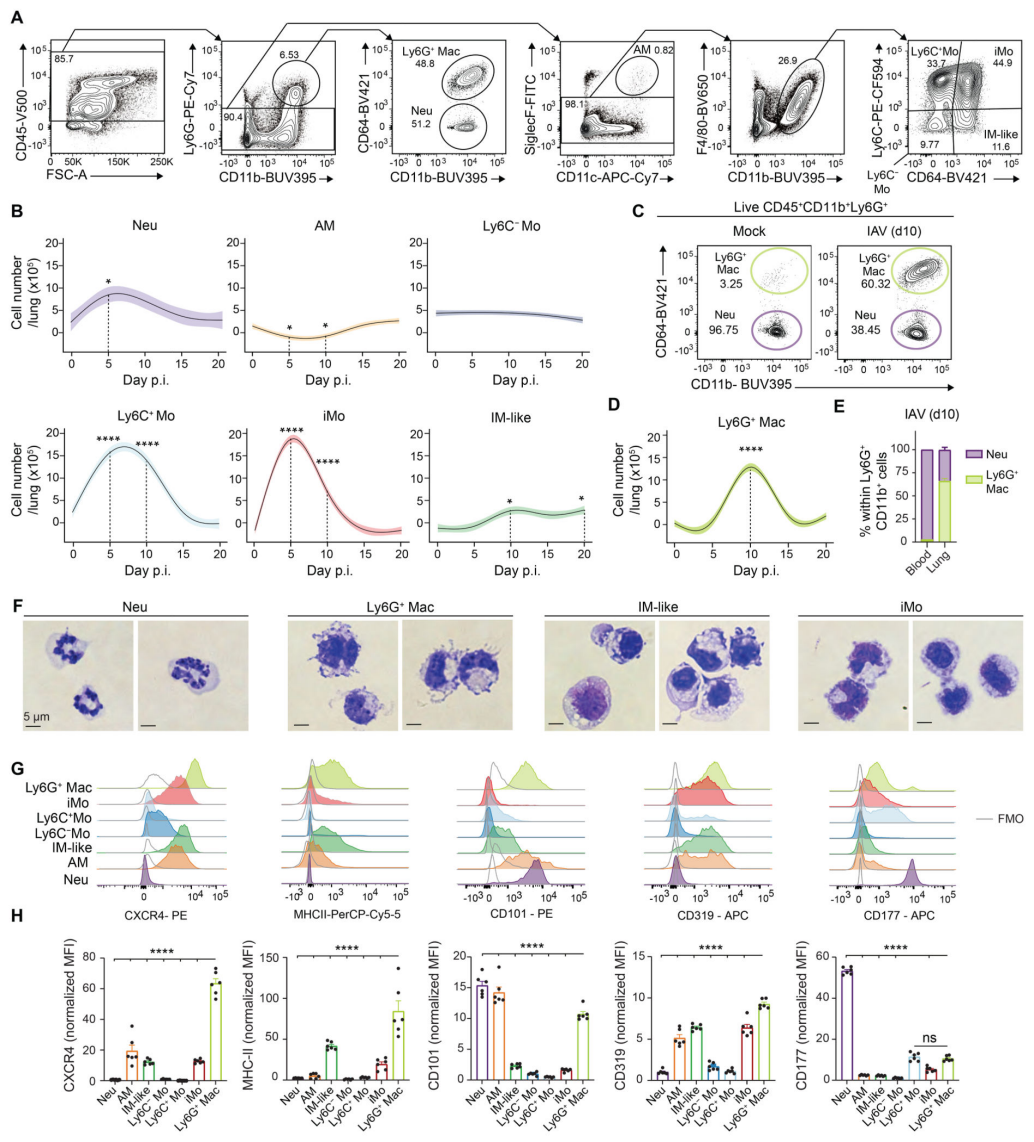
Ly6G^+^ Macs culminate during the early recovery phase post-IAV infection. (**A**) Representative flow cytometry gating strategy showing live CD45^+^CD11b^+^Ly6G^+^CD64^−^ neutrophils (Neu), CD45^+^CD11b^+^Ly6G^+^CD64^+^ macrophages (Ly6G^+^ Mac), CD45^+^Ly6G^−^CD11c^+^SiglecF^+^ alveolar macrophages (AM), CD45^+^Ly6G^−^SiglecF^−^F4/80^+^CD11b^+^Ly6C^+^CD64^−^ monocytes (Ly6C^+^ Mo), CD45^+^Ly6G^−^SiglecF^−^F4/80^+^CD11b^+^Ly6C^−^CD64^−^ monocytes (Ly6C^−^ Mo), CD45^+^Ly6G^−^SiglecF^−^F4/80^+^CD11b^+^Ly6C^+^CD64^+^ inflammatory monocytes (iMo) and CD45^+^Ly6G^−^SiglecF^−^F4/80^+^CD11b^+^Ly6C^−^CD64^+^ IM-like cells in lungs of C57BL/6 wild-type (WT) mice at day 10 post-IAV. (**B**) Time course of absolute numbers of Neu, AM, Ly6C^−^ Mo, Ly6C^+^ Mo, iMo and IM-like cells quantified by flow cytometry at days 0, 5, 10, 15 and 20 post-IAV in WT mice. (**C**) Representative contour plots of CD64 and CD11b expression within lung CD45^+^CD11b^+^Ly6G^+^ cells at day 10 p.i. in mock-infected or IAV-infected WT mice. (**D**) Time course of absolute numbers of Ly6G^+^ Macs quantified by flow cytometry, as in (B). (**E**) Percentage of Neu and Ly6G^+^ Macs within Ly6G^+^CD11b^+^ cells quantified by flow cytometry in the blood and lungs of WT mice at day 10 post-IAV. (**F**) Photographs of Neu, Ly6G^+^ Macs, IM-like cells and iMo sorted by FACS from IAV-infected WT mice at day 10 p.i.. Pictures are representative of 1 of 3 independent sorting experiments, each giving similar results. (**G**) Representative histograms of CXCR4, MHC-II, CD101, CD319 and CD177 expression in the indicated myeloid cell populations, quantified by flow cytometry at day 10 post-IAV in WT mice. (**H**) Quantification of expression of the indicated markers, as in (G). (B,D) Data show mean (centerline) ± SEM (colored area) and are pooled from 2-3 independent experiments (*n*=6 mice per time point); (E,H) mean + SEM and are pooled from 2 independent experiments (*n*=5-6 mice). (B,D,H) *P* values were calculated using a one-way ANOVA with Dunnett’s post hoc tests. *, *P*<0.05; ***, *P*<0.001; ****, *P*<0.0001. FMO, fluorescence minus one; ns, not significant; p.i., post-infection. (F) Scale bars: 5 μm.

To investigate the dynamic of myeloid cell responses after lung injury, we performed time-course flow cytometry studies in a clinically relevant mouse model of lung infectious injury following IAV infection (2). Eight to twelve weeks-old C57BL/6 wild-type (WT) mice were infected intranasally (i.n.) with 5 plaque-forming units (PFU) of IAV H1N1 strain PR8/34, which triggered self-limiting disease with a peak in viral RNA at day 5 post-IAV and viral clearance at day 10 post-IAV (fig. S1, A and B). In this model, Ly6G^+^CD11b^+^CD64^−^ neutrophils (Neu) increased at day 5 post-IAV and returned to baseline by day 15 (Fig. 1, A and B). A partial loss of Ly6G^−^CD64^+^SiglecF^+^CD11c^+^ AM was observed between day 5 and day 10 post-IAV, which was restored at day 15 post-IAV, as described (15) (Fig. 1, A and B). The numbers of CD64^−^Ly6C^−^ monocytes (Ly6C^−^ Mo) remained stable over the course of infection, unlike those of classical CD64^−^Ly6C^+^ Mo (Ly6C^+^ Mo) and inflammatory CD64^+^Ly6C^+^ monocytes (iMo) that peaked around day 5 post-IAV (Fig. 1, A and B). Macrophages (Macs) resembling IM (IM-like), defined as F4/80^+^CD11b^+^Ly6G^−^SiglecF^−^Ly6C^−^CD64^+^ cells and likely encompassing resident IM and recruited Mo-Macs, increased over time (Fig. 1, A and B). We also observed emergence, from day 5 onwards, of a distinct population of IAV-triggered Ly6G^+^CD11b^+^CD64^+^ Macs that fell in the classical Ly6G^+^CD11b^+^ neutrophil gate but were clearly distinct from neutrophils based on their elevated CD64 expression (Fig. 1, C and D), which we call Ly6G^+^ Macs hereafter. Ly6G^+^ Macs were absent in the blood (Fig. 1E), peaked at day 10 post-IAV in the lung and could still be detected at days 15 and 20 post-IAV (Fig. 1D). Morphologically, Ly6G^+^ Macs analyzed at day 10 post-IAV exhibited a kidney-shaped nucleus, like iMo, and possessed numerous cytoplasmic vacuolated structures and a cell membrane rich in protrusions (Fig. 1F and fig. S1C). Phenotypically, Ly6G^+^ Macs were F4/80^hi^SiglecF^lo^CD11c^lo^ (fig. S1, D and E) and expressed high levels of the chemokine receptor CXCR4, of type II major histocompatibility complex (MHC-II), of CD101 and of CD319, a regulator of Mac functions (26, 27) (Fig. 1, G and H). However, Ly6G^+^ Macs exhibited low expression of the neutrophil activation marker CD177 (Fig. 1, G and H).

**Fig. 2:**
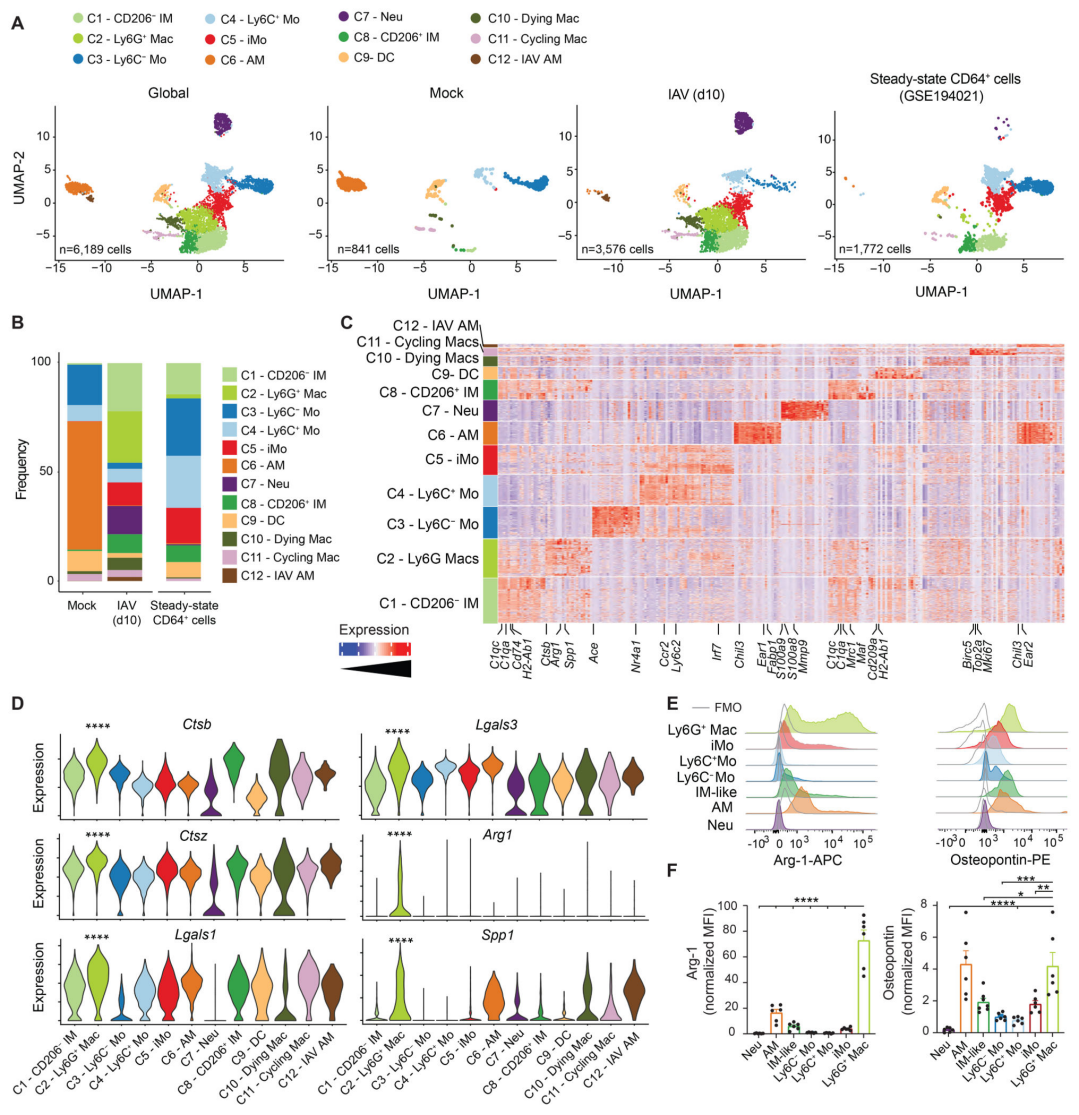
Ly6G^+^ Macs are transcriptionally distinct from other lung myeloid cells at day 10 post-IAV. (**A**) UMAP plots of scRNA-seq data depicting the transcriptional identity of FACS-sorted lung live CD45^+^F4/80^+^ and/or CD11b^+^ cells from mock- or IAV-infected WT mice 10 days p.i. (pooled from 5 mice per conditions), merged with a published dataset of steady-state lung monocytes and IMs (29). (**B**) Frequency of each cluster within each experimental condition, as in (A). (**C**) Heatmap depicting the single cell expression of the most upregulated genes within each cluster. (**D**) Expression of the indicated genes within each cluster, as depicted by violin plots (height: expression; width: abundance of cells). (**E**) Representative histograms of intracellular Arg-1 and osteopontin expression in the indicated lung myeloid cell populations, quantified by flow cytometry at day 10 post-IAV in WT mice. (**F**) Quantification of Arg-1 and osteopontin expression, as in (E). (F) Data show mean + SEM and are pooled from 2 independent experiments (*n*=6 mice). *P* values were calculated using (D) a Wilcoxon rank sum test and compare C2 vs. all other clusters or (F) a one-way ANOVA with Dunnett’s post hoc tests. *, *P*<0.05; **, *P*<0.01; ***, *P*<0.001; ****, *P*<0.0001. FMO, fluorescence minus one; p.i., post-infection.

Next, we performed single cell (sc) RNA-sequencing (scRNA-seq) analyses of lung myeloid cells at day 10 post-IAV. Lung CD45^+^F4/80^+^ and/or CD11b^+^ cells were sorted from five mock-infected and 5 IAV-infected mice and were subjected to sc droplet encapsulation (28), scRNA-seq and quality control filtering. The curated data were integrated with a published dataset of steady-state lung monocytes and IM (29) and projected to global and condition-specific uniform manifold approximation and projection (UMAP) plots (Fig. 2A). Myeloid cells from mock-infected mice mainly comprised clusters annotated as AM (*Chil3, Ear1, Fapb1*; C6), Ly6C^−^ Mo (*Ace, Nr4a1, Fcgr4*; C3), Ly6C^+^ Mo (*Ccr2, Ly6c2*; C4) and dendritic cells (DC; *H2-Ab1, Cd209a, Flt3;* C9) (Fig. 2A-C and fig. S2A). Few cycling Macs (*Birc5, Top2a, Mki67*; C11) were also detected in mock-infected mice, along with few CD206^−^ IM (*C1qa, C1qc, H2-Ab1, Cd74, Tmem119;* C1) and CD206^+^ IM (*C1qa, C1qc, Mrc1, Maf;* C8) (29) (Fig. 2A-C and fig. S2A). Ten days post-IAV, AM (C6) disappeared, a small cluster of IAV-associated AM was present instead (*Chil3, Ear1, Ear 2*; C12), Neu were recruited (*S100a8, S100a9, Mmp9*; C7), and IM (C1, C8) and iMo (*Ccr2, Ly6c2, Irf7*; C5) expanded (Fig. 2A-C and fig. S2A). Of note, clusters C2 and C10 were specifically triggered by IAV. C10 had an elevated content in mitochondrial genes and low numbers of detected genes (fig. S2B) and was therefore annotated as dying Macs. C2 expressed significantly higher levels of cathepsins (*Ctsb, Ctsz*), galectins (*Lgals1, Lgals3*), Arginase-1 (*Arg1*) and osteopontin (*Spp1*) as compared to the other clusters (Fig. 2D). Intracellular flow cytometry staining for Arginase-1 and osteopontin showed that the combined expression of these two proteins was restricted to Ly6G^+^ Macs at day 10 post-IAV (Fig. 2, E and F), supporting that C2 corresponded to Ly6G^+^ Macs identified by flow cytometry. Of note, Ly6G^+^ Macs expressed high levels of *Csf1r* and *Slamf7* (coding for CD319) but did not express any of the neutrophil-related transcripts *Csf3r, S100a8, S100a9, Mmp8, Mmp9, Mpo, Slpi* or *Cd177* (fig. S2, A and C). *Ly6g* transcripts were not detectable in Neu nor Ly6G^+^ Macs (fig. S2, A and C). Ly6G^+^ Macs (C2) displayed both “M1-like” or “M2-like” signature scores and genes and could not be categorized as such on the basis of their expression profile (fig. S2, D and E). Together, our data show that a phenotypically and transcriptionally distinct subset of Ly6G^+^ Macs emerges during a specific time window corresponding to early weight recovery post-IAV.

### Ly6G^+^ Macs arise from recruited monocytes and are partially dependent on GM-CSF receptor signaling

We next investigated the origin of Ly6G^+^ Macs and asked whether they could expand via local proliferation. We observed that the percentage of cells positive for the proliferation Ki67 was very low in Ly6G^+^ Macs (fig. S3A). Next, we treated mice at day 10 post-IAV intraperitoneally (i.p.) with 5-ethynyl-2′-deoxyuridine (EdU) 4 hours before analysis. While the percentage of EdU^+^ cells was higher in AM compared to all other lung myeloid cells, indicative of active proliferation, virtually no EdU^+^ cells were detected in Ly6G^+^ Macs, ruling out their active proliferation (fig. S3, B and C). Third, we assessed whether Ly6G^+^ Macs arose from the BM or from local progenitor monocytes (30). We generated chimeric mice in which lethally irradiated, thorax-protected CD45.2 WT mice were reconstituted with BM cells from *Ms4a3*^Cre^*R26*^*LSL*tdTomato^ mice (subsequently referred to as *Ms4a3^tdTom^*), in which the progeny of granulocyte monocyte progenitors (GMPs) is constitutively labelled (31). At week 4 after transfer, the percentages of tdTomato^+^ blood Ly6C^+^ Mo was around 50%, while the percentages of tdTomato^+^ lung AM and IM were very low, confirming efficient reconstitution and thorax protection (fig. S3, D-G). At day 10 post-IAV, we found that the percentage of tdTomato^+^ Ly6G^+^ Macs was similar to that of Ly6C^+^ Mo (fig. S3, H and I), consistent with a major contribution of BM-derived GMPs, but not local progenitors, to Ly6G^+^ Macs.

**Fig. 3:**
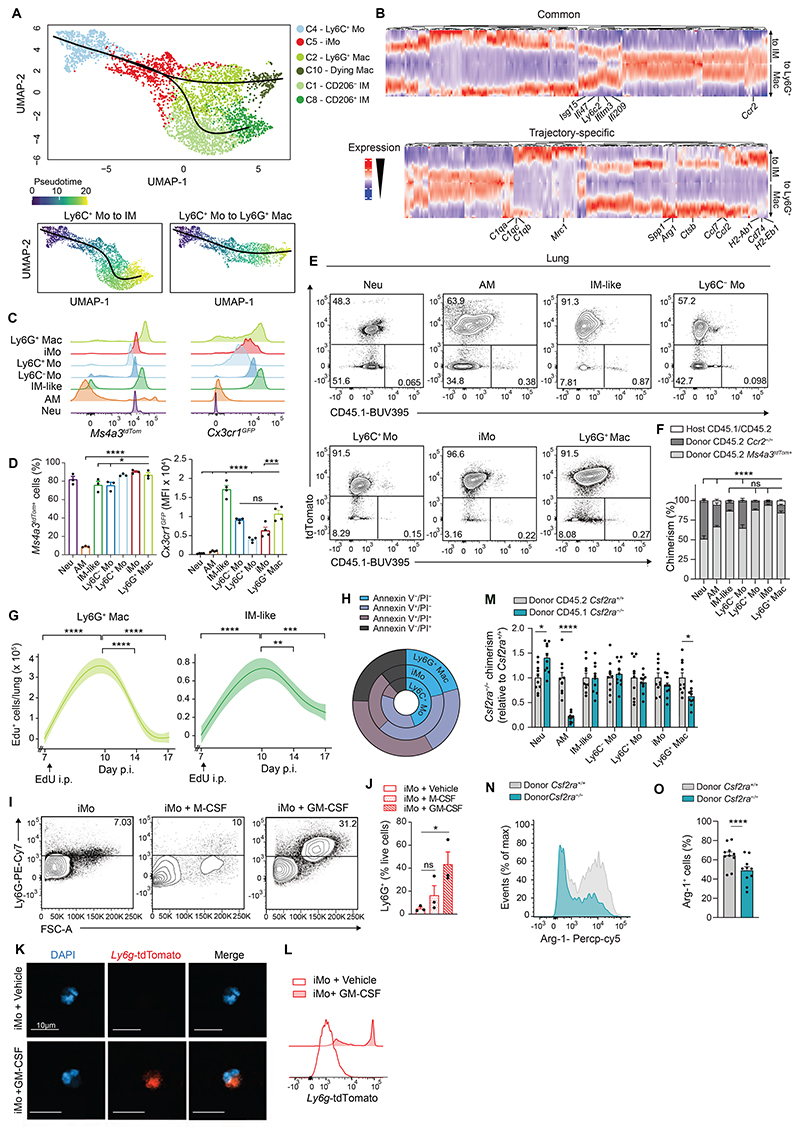
IAV-triggered Ly6G^+^ Macs are recruited from classical monocytes and are short-lived. (**A**) UMAP plot depicting the transcriptional identity and cell trajectories (top), and pseudotime trajectory values (below) of lung Ly6C^+^ Mo, iMo, Ly6G^+^ Mac, dying Mac, CD206^−^ IM and CD206^+^ IM, as in Fig. 2A, evaluated by Slingshot trajectory analyses. (**B**) Heatmap plot depicting the differentially expressed genes along pseudotime evaluated by tradeSeq in the trajectory starting from Ly6C^+^ Mo and ending either in IM or in Ly6G^+^ Mac. (**C**) Representative histograms of tdTomato (left) and GFP (right) expression in the indicated myeloid cell populations, quantified by flow cytometry at day 10 post-IAV in *Ms4a3^tdTom^* and *Cx3Cr1^GFP^* mice, respectively. (**D**) Quantification of tdTomato^+^ cells (left) and GFP expression (right), as in (C). (**E**) Representative tdTomato and CD45.1 contour plots and (**F**) bar graph showing % of host, donor *Ccr2*^−/−^ and donor *Ms4a3^tdTom+^* chimerism in the indicated cell populations from lethally-irradiated CD45.1/CD45.2 mice reconstituted with a 1:1 mix of CD45.2 *Ccr2*^−/−^ and *Ms4a3^tdTom+^* BM cells, infected with IAV 4 weeks later and evaluated at day 10 post-IAV. (**G**) Time course of absolute numbers of EdU^+^ Ly6G^+^ Macs and EdU^+^ IM-like cells quantified by flow cytometry at days 7, 10, 14 and 17 post-IAV in EdU-pulsed WT mice at day 7 post-IAV. (**H**) Pie chart representation of the mean frequency of Annexin V and PI negative and/or positive fractions within lung Ly6C^+^ Mo, iMo and Ly6G^+^ Macs, quantified at day 10 post-IAV in WT mice. (**I**) Representative Ly6G and FSC contour plots and (**J**) bar graph showing % of Ly6G^+^ cells within lung iMo sorted from WT mice at day 10 post-IAV and cultured 18 hours *ex vivo* with vehicle, M-CSF or GM-CSF. (**K**) Representative confocal microscopy pictures and (**L**) representative flow cytometry histograms of tdTomato expression within lung iMo sorted from *Ly6g^tdTom^* mice at day 10 post-IAV and treated *ex vivo* with tamoxifen and GM-CSF or vehicle for 18 hours. (**M**) Bar graph showing donor *Csf2ra*^−/−^ chimerism relative to donor *Csf2ra^+/+^* chimerism in the indicated cell populations from thorax-protected, lethally-irradiated CD45.1/CD45.2 mice reconstituted with a 1:1 mix of CD45.1 *Csf2ra*^−/−^ and CD45.2 *Csf2ra^+/+^* BM cells, infected with IAV 4 weeks later and evaluated at day 10 post-IAV. (**N**) Representative histograms and (**O**) quantification of Arg-1^+^ cells (%) in Ly6G^+^ Macs from donor *Csf2ra^+/+^* and *Csf2ra*^−/−^ BM cells, as in (M). Data show (D,F,J,M,O) mean + SEM and (D,F) are representative of 1 of 3 independent experiments (*n*=3-4 mice), (J) are pooled from 3 independent sorting experiments, each dot representing one biological replicate, (M,O) are pooled from 2 independent experiments (*n*=10 mice); (G) mean (centerline) ± SEM (colored area) and are pooled from 2 independent experiments (*n*=6 mice per time point). *P* values compare CD45.2 donor *Ccr2*^−/−^ chimerism in (F). *P* values were calculated using (D) a one-way ANOVA with Dunnett’s post hoc tests, (F) a two-way ANOVA with Tukey’s post hoc tests, (G,J) a one-way ANOVA with Tukey’s post hoc tests, (M) a two-way ANOVA with Sidak’s post hoc tests, (O) a two-tailed Student’s *t* test. *, *P*<0.05; ***, *P*<0.001; ****, *P*<0.0001. FMO, fluorescence minus one; ns, not significant.

The kinetics of Ly6G^+^ Mac emergence post-IAV was comparable but delayed compared to that of Ly6C^+^ Mo and iMo, consistent with the idea that recruited Ly6C^+^ Mo could give rise to Ly6G^+^ Macs. Supporting this, Slingshot trajectory analyses of the scRNA-seq data identified two main trajectories starting from Ly6C^+^ Mo, transiting through iMo to give rise to either IM-like cells or Ly6G^+^ Macs (32) (Fig. 3A). Genes that exhibited the same pattern of downregulation along pseudotime in each trajectory encompassed the classical monocyte-associated genes *Ccr2* and *Ly6c2* (Fig. 3B). We also found, in both trajectories, a time-restricted upregulation of interferon-stimulated genes (*Ifi209, Ifitm3, Ifi47, Isg15*) that likely corresponded to transitioning iMo (Fig. 3B). Finally, trajectory-specific genes were gradually and specifically upregulated along pseudotime in the IM-like (e.g., *C1qa, C1qc, C1qb, Mrc1, Cd74, H2-Ab1, H2-Eb1*) or the Ly6G^+^ Mac (e.g., *Arg1, Spp1, Ccl2, Ccl7, Ctsb*) trajectories (Fig. 3B).

Next, we infected the monocyte fate-mapper mice *Ms4a3^tdTom^* (31) and *Cx3cr1*^GFP^ (33) with IAV and found that, at day 10 post-IAV, Ly6G^+^ Macs were Tomato^+^ and GFP^hi^, respectively, consistent with their GMP-derived monocytic origin (Fig. 3, C and D). To address the dependency of Ly6G^+^ Mac on Ccr2-dependent BM-derived Ly6C^+^ Mo (34), we generated BM competitive chimeras in which lethally irradiated CD45.1/CD45.2 WT mice were engrafted with a 1:1 mix of CD45.2 *Ccr2*^−/−^ and CD45.2 *Ms4a3^tdTom^* BM cells. At week 4 after reconstitution, most blood Ly6C^+^ Mo were of donor Tomato^+^ origin, as expected (fig. S3, J and K) (34). At day 10 post-IAV, the majority of Ly6G^+^ Macs was also of donor *Ms4a3^tdTom^* origin, indicating their dependency on Ccr2 (Fig. 3, E and F).

We next sought to assess the fate and lifespan of Ly6G^+^ Macs. The abundance of Ly6G^+^ Macs during a limited time window post-IAV suggested that they might be short-lived. In line with this, trajectory analyses in Fig. 3A suggested that Ly6G^+^ Macs (C2) gave rise to Macs with low RNA content (C10) (fig. S2B). We performed EdU pulse experiments at day 7 post-IAV and found that Ly6G^+^ Macs staining positive for EdU at day 10 post-IAV were completely cleared from the lung at day 17 (Fig. 3G). Annexin V/Propidium iodide (PI) staining at day 10 post-IAV further supported that Ly6C^+^ Mo transitioning to iMo and differentiating into Ly6G^+^ Macs became progressively more sensitive to death, with a substantial portion of Ly6G^+^ Macs being either early or late apoptotic (Annexin V^+^/PI^+/−^), or necrotic (Annexin V^−^/PI^+^) (Fig. 3H and fig. S3L). Hence, our data show that Ly6G^+^ Macs represent a short-lived Mac subset arising from Ccr2-dependent Ly6C^+^ Mo.

The Ly6G signal on Ly6G^+^ Macs, considered to be neutrophil-specific, was unexpected and required careful validation. First, we verified that the Ly6G fluorescence intensity was virtually absent in unstained or isotype antibody (Ab)-stained CD11b^+^ cells from IAV-infected WT mice (fig. S4A). Second, we evaluated whether Ly6G^+^ Mac precursors, namely iMo, could intrinsically upregulate Ly6G on their surface when isolated from the lungs of IAV-infected mice at day 10 post-IAV. We found that iMo from IAV-infected mice and stimulated *ex vivo* with GM-CSF, and to a less extent with M-CSF, upregulated Ly6G protein on their surface (Fig. 3, I and J). Importantly, we also found that lung iMo isolated from IAV-infected *Ly6g^CreERT2^R26*^*LSL*tdTomato^ mice (*Ly6g^tdTom^*) treated with tamoxifen and stimulated with GM-CSF ex vivo became tdTomato^+^, indicative of active *Ly6g* gene transcription in monocytic cells (Fig. 3, K and L). These data demonstrate that Ly6G can be actively expressed by Mo-Macs.

Given the ability of GM-CSF to trigger Ly6G expression on lung iMo isolated from IAV-infected mice, we assessed the dependency of Ly6G^+^ Macs on GM-CSF receptor signaling *in vivo*. We generated BM competitive chimeras in which thorax-protected, lethally irradiated CD45.1/CD45.2 WT mice were engrafted with a 1:1 mix of CD45.1 *Csf2ra*^-/-^ and CD45.2 *Csf2ra*^+/+^ BM donor cells. At week 4 after reconstitution, blood Neu and Mo of donor origin arose equally from CD45.1 *Csf2ra*^−/−^ and CD45.2 *Csf2ra*^+/+^ BM cells (fig. S4, B and C). At day 10 post-IAV, we found that CD45.2 *Csf2ra*^+/+^ BM cells had a competitive advantage over CD45.1 *Csf2ra*^−/−^ BM cells to become Ly6G^+^ Macs, which was not observed among most other lung myeloid cells (Fig. 3M and fig. S4C-E), and the percentage of Arg-1^+^ cells was lower in *Csf2ra*^−/−^ Ly6G^+^ Macs as compared to *Csf2ra*^−/−^ Ly6G^+^ Macs (Fig. 3, N and O), indicating their partial dependency on GM-CSF receptor signaling.

### Ly6G^+^ Macs exhibit distinct ultrastructural features associated with elevated metabolic and phagocytic abilities

**Fig. 4:**
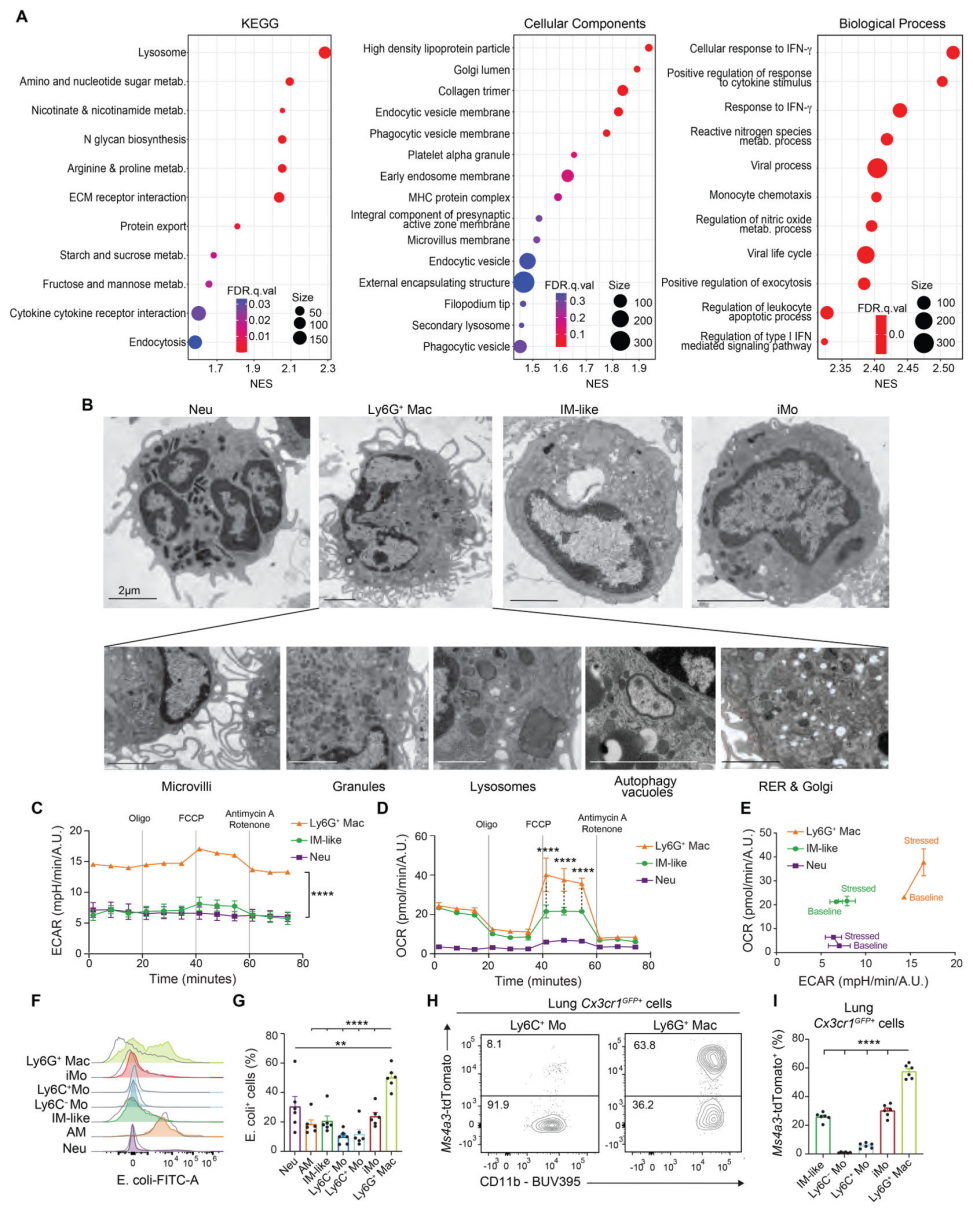
Ly6G^+^ Macs exhibit an atypical morphology and possess distinct metabolic, phagocytic and efferocytic capabilities. (**A**) GSEA analyses of Ly6G^+^ Mac (C2) profile compared to other clusters using KEGG, Cellular Components and Biological Process gene sets. The Normalized Enrichment Score (NES), False Discovery Rate (FDR) and the size of the gene set are shown for each process. (**B**) Representative transmission electron microscopy pictures of Neu, Ly6G^+^ Mac, IM-like cells and iMo FACS-sorted from lungs of WT mice at day 10 post-IAV. (**C**) Extracellular acidification rate (ECAR) of FACS-sorted Ly6G^+^ Mac, Neu and IM-like cells, as in (B), quantified at baseline and under stress over time using a Seahorse assay. (**D**) Oxygen consumption rate (OCR) of Ly6G^+^ Mac, Neu and IM-like cells, as in (C). (**E**) ECAR and OCR of Ly6G^+^ Macs, Neu and IM-like cells, as in (C,D). (**F**) Representative histograms of E. coli-FITC signal in the indicated myeloid cell populations, quantified by flow cytometry at day 10 post-IAV and 3 hours after i.t. injection of E. coli-FITC particles. Non-injected mice were used as controls (grey line). (**G**) Quantification of E. coli-FITC^+^ cells, as in (F). (**H**) Representative tdTomato and CD11b contour plots of and (**I**) bar graph showing % of tdTomato^+^ cells in the indicated *Cx3cr1^GFP+^* donor cell populations from lethally-irradiated CD45.2 WT mice reconstituted with a 1:1 mix of CD45.2 *Cx3cr1^GFP+^* and *Ms4a3^tdTom+^* BM cells, infected with IAV 4 weeks later and evaluated at day 10 post-IAV. Data show (C,D) mean ± SEM and are representative of 1 of 3 independent experiments, each giving similar results; (G,I) mean + SEM and are pooled from 2 independent experiments (*n*=6 mice). *P* values (C,D) compare Ly6G^+^ Macs vs. IM-like cells or Neu and were calculated using a two-way ANOVA with Bonferroni’s post hoc tests; (G,I) were calculated using a one-way ANOVA with Dunnett’s post hoc tests. **, *P*<0.01; ****, *P*<0.0001. (B) Scale bars: 2 μm.

Gene Set Enrichment Analyses (GSEA) of the transcriptomic profile of Ly6G^+^ Macs (C2) compared to all other clusters identified a response to interferon-γ and cytokines, chemotactic and viral processes, an active metabolic state, a highly developed endomembrane system and elevated phagocytic abilities in Ly6G^+^ Macs (Fig. 4A). We analyzed FACS-sorted Ly6G^+^ Macs by transmission electron microscopy at day 10 post-IAV and found that they exhibited a kidney-shaped nucleus, microvilli-rich membrane, secretory granules and a cytoplasm rich in rugous endoplasmic reticulum (RER), Golgi apparatus, lysosomes and autophagy vacuoles, distinguishing them from Neu, Ly6C^+^ Mo and IM-like cells (Fig. 4B). The morphology of Ly6G^+^ Macs was reminiscent to that of an atypical population of monocytes, called SatM monocytes, which arise from particular GMPs during the fibrotic phase in lungs post-bleomycin, contribute to fibrosis and were regulated by CCAAT/enhancer binding protein β (C/EBPβ) (35). We conducted a single-cell regulatory network inference and clustering (SCENIC) analysis (36) and found that C/EBPβ activity was lower in Ly6G^+^ Macs compared to Ly6C^−^ Mo, Ly6C^+^ Mo, iMo and Neu (fig. S5A). Moreover, we generated a SatM signature score based on the genes unregulated in SatM monocytes (35), mapped such score to our scRNA-seq data and found that Ly6G^+^ Macs displayed a lower SatM score compared to IM, Ly6C^+^ Mo, Ly6C^-^ Mo or iMo (fig. S5, B and C), supporting that Ly6G^+^ Macs are not dependent on C/EBPβ and are transcriptionally distinct from SatM monocytes (35).

Next, we characterized the metabolic profile of Ly6G^+^ Macs using a metabolic flux assay and found that the extracellular acidification rate (ECAR) was higher in Ly6G^+^ Mac compared to IM-like cells, both at baseline and under stress, supporting that the glycolytic pathway was highly active in Ly6G^+^ Macs (Fig. 4C). Moreover, while the basal mitochondrial oxygen consumption rate (OCR) was similar between Ly6G^+^ Mac and IM-like cells, the OCR under stress conditions was higher in Ly6G^+^ Macs compared to IM-like cells (Fig. 4D), supporting that they possessed a high metabolic potential (Fig. 4E).

We administered infected mice with fluorescently-labeled E. coli particles intratracheally (i.t.) at day 10 post-IAV and confirmed that Ly6G^+^ Macs were highly phagocytic compared to Neu, Ly6C^+^ Mo, IM-like cells and AM (Fig. 4, F and G). Hence, we asked whether Ly6G^+^ Macs could perform efferocytosis *in vivo*. To this end, we infected BM competitive chimeras in which lethally irradiated CD45.2 WT mice were engrafted with a 1:1 mix of *Cx3cr1^GFP/+^* and *Ms4a3^tdTom^* BM cells. Of note, at day 10 post-IAV, 60% of *Cx3cr1^GFP/+^* Ly6G^+^ Macs were also tdTomato^+^ (Fig. 4, H and I and fig. S6, A-C), demonstrating that they were highly potent in engulfing myeloid cells *in vivo*. To assess whether Ly6G molecules could be transferred from Neu to Ly6G^+^ Macs during efferocytosis, we infected BM competitive chimeras in which lethally irradiated CD45.1/CD45.2 WT mice were engrafted with a 1:1 mix of *Cx3cr1^GFP/+^* cells and *Ly6g*^−/−^ (i.e., homozygous *Ly6g^CreERT2^* mice) (37) or *Ly6g^+/+^* BM cells. At day 10 post-IAV, we found that the levels of Ly6G on *Cx3cr1^GFP+^* Ly6G^+^ Macs from *Cx3cr1^GFP+^: Ly6g*^−/−^ BM chimeric mice, in which half of the Neu were *Ly6g*^−/−^, were similar to those from *Cx3cr1^GFP+^: Ly6g^+/+^* BM chimeric mice, supporting no evidence for a Ly6G transfer from Neu to Ly6G^+^ Macs (fig. S6, D and E). Together, these data show that IAV-triggered Ly6G^+^ Macs are characterized by metabolic, morphological and efferocytic properties distinct from other lung myeloid cells.

### Ly6G^+^ Macs populate the alveolar lumen of perilesional regenerating areas

**Fig. 5:**
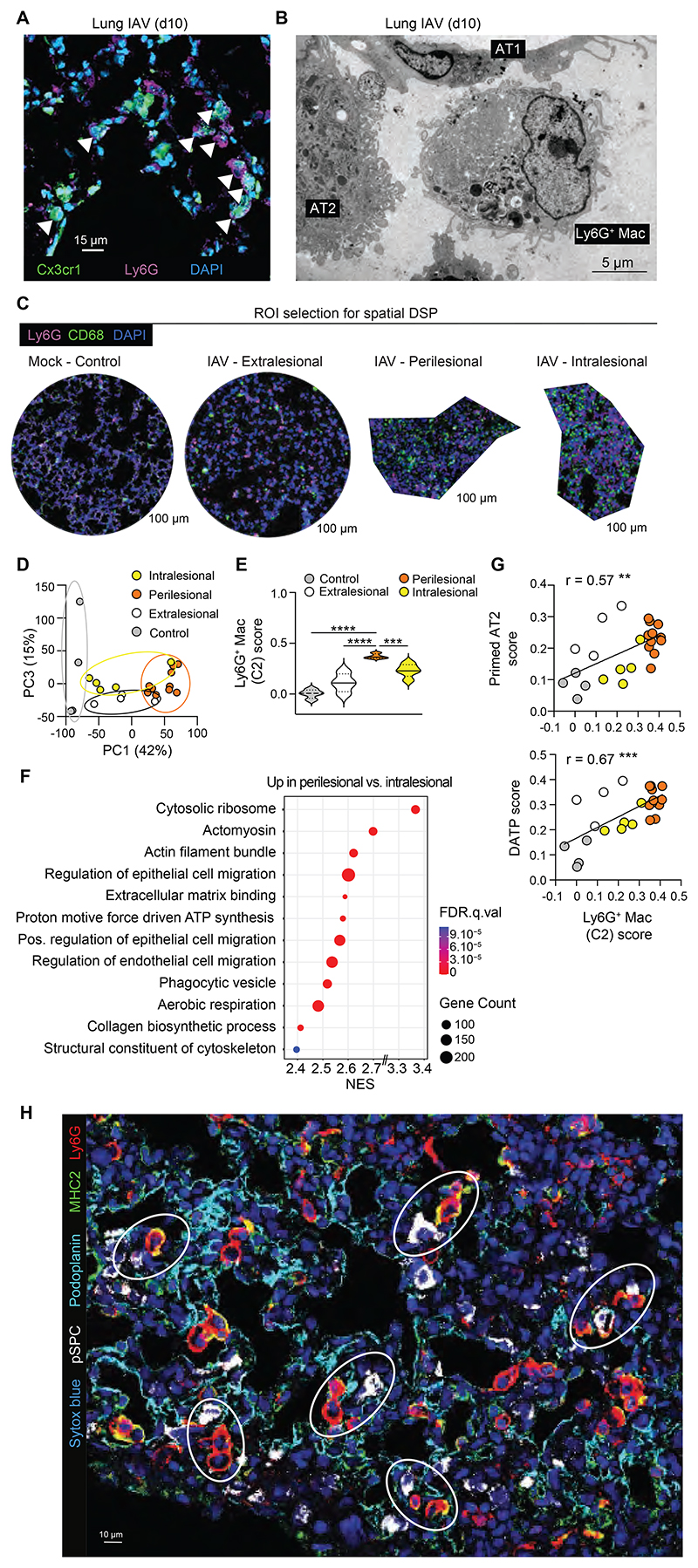
Ly6G^+^ Macs populate the alveoli of perilesional regenerating areas. (**A**) Representative confocal microscopy picture of lung sections from *Cx3cr1^GFP^* mice at day 10 post-IAV, with Ly6G^+^ Macs identified as Ly6G^+^*Cx3cr1^GFP+^* cells. (**B**) Representative *in situ* electron microscopy picture of Ly6G^+^ Macs in the vicinity of AT2 and AT1 cells, identified on lung sections from WT mice at day 10 post-IAV. (**C**) Representative examples of regions of interest (ROIs) selected on lung sections from mock- or IAV-infected WT mice at day 10 post-IAV stained with anti-Ly6G and anti-CD68 antibodies. (**D**) Unsupervised Principal Component (PC) analysis of the ROIs analyzed by DSP. Percentages indicate the variability explained by each component. (**E**) Ly6G^+^ Mac signature score within control, extralesional, perilesional and intralesional ROIs, as depicted by violin plots (height: scores; width: abundance of cells). (**F**) GSEA analysis of perilesional ROIs compared to intralesional ROIs using Cellular Components, Molecular Function and Biological Process gene sets. The Normalized Enrichment Score (NES), False Discovery Rate (FDR) and the size of the gene set are shown for each process. (**G**) Correlation of Ly6G^+^ Mac score with primed AT2 (top) and DATP (bottom) scores of the ROIs. (**H**) Representative picture of perilesional Ly6G^+^ Macs (i.e., Ly6G^+^MHC-II^+^ cells), pSPC^+^ AT2 and podoplanin^+^ AT1 identified by confocal microscopy on lung sections from WT mice at day 10 post-IAV. (A,B,H) Pictures are representative of one of 6 mice, each giving similar results. (E) *P* values were calculated using a one-way ANOVA with Tukey’s post hoc tests. (G) The correlation analysis used was parametric Pearson correlation coefficient. **, *P*<0.01; ***, *P*<0.001. Scale bars: (A) 15, (B) 5, (C) 100, (D) 10 μm.

Next, we investigated the localization and the spatial organization of Ly6G^+^ Macs. First, we performed confocal microscopy staining of lung sections of infected *Cx3cr1*^GFP^ mice at day 10 post-IAV. By defining Ly6G^+^ Macs as cells double positive for Ly6G and GFP, we found that they were located in the alveolar lumen (Fig. 5A), which was also confirmed by *in situ* electron microscopy (Fig. 5B).

To further investigate the spatial distribution of Ly6G^+^ Macs and the molecular signatures of Ly6G^+^ Macs-rich areas, we performed spatial transcriptomic analyses using GeoMx Digital Spatial Profiler (DSP), which allows whole-genome transcript analyses within regions of interest (ROIs). Lung sections from 2 mock- and 4 IAV-infected mice were collected at day 10 post-IAV, stained with anti-CD68 and anti-Ly6G antibodies, and ROIs were selected in control lungs (4 ROIs), extralesional zones (4 ROIs), intralesional zones (5 ROIs) and zones rich in Ly6G^+^CD68^+^ cells that were mostly located in the periphery of consolidated areas (perilesional, 11 ROIs) (Fig. 5C and fig. S7A). Unsupervised principal component (PC) analysis showed that perilesional ROIs were separated from the other regions (Fig. 5D). Volcano plot representation of the differentially expressed (DE) genes between conditions and the heatmap of the 522 significantly upregulated genes in perilesional areas compared to the other areas supported that perilesional areas were transcriptionally very active (fig. S7, B and C). General cellular deconvolution indicated that perilesional zones were also enriched in tissue Macs as compared to the other regions (fig. S7D). We next mapped cell signature scores of lung myeloid cell populations analyzed by scRNA-seq to the ROIs and confirmed that perilesional zones were enriched in Ly6G^+^ Mac compared to the other zones (Fig. 5E and fig. S7E). GSEA analyses indicated that perilesional areas were enriched in biological responses related to cytoskeleton activity, epithelial cell migration and elevated metabolic activity compared to intralesional areas, consistent with intense remodeling activities (Fig. 5F).

Next, we took advantage of a publicly available scRNA-seq dataset of alveolar epithelial cell states present during alveolar regeneration after bleomycin-induced lung injury (38) and containing type 1 and type 2 alveolar epithelial cells (AT1 and AT2, respectively), as well as transitional states appearing during AT2 to AT1 differentiation, called primed AT2 and damage-associated transient progenitors (DATPs) (38). By mapping the signature scores of such transitional epithelial cell states to the ROIs, we found that Ly6G^+^ Mac-rich perilesional zones were enriched in primed AT2 and DATPs compared to control and intralesional zones (fig. S7, F and G). Accordingly, the Ly6G^+^ Mac score correlated positively with those of primed AT2 and DATPs (Fig. 5G). We also confirmed by confocal microscopy that Ly6G^+^ Macs were particularly abundant in the periphery of consolidated areas and clustered with AT2 cells, while intralesional consolidated areas, which exhibited low levels of staining for AT1 and AT2, contained few Ly6G^+^ Macs (Fig. 5H and fig. S8). Altogether, these data are consistent with perilesional areas serving as the site of active epithelial regeneration post IAV, and that Ly6G^+^ Macs, which cluster in such areas, contribute to this process.

### Ly6G^+^ Macs promote alveolar epithelial regeneration through IL-4R signaling

**Fig. 6:**
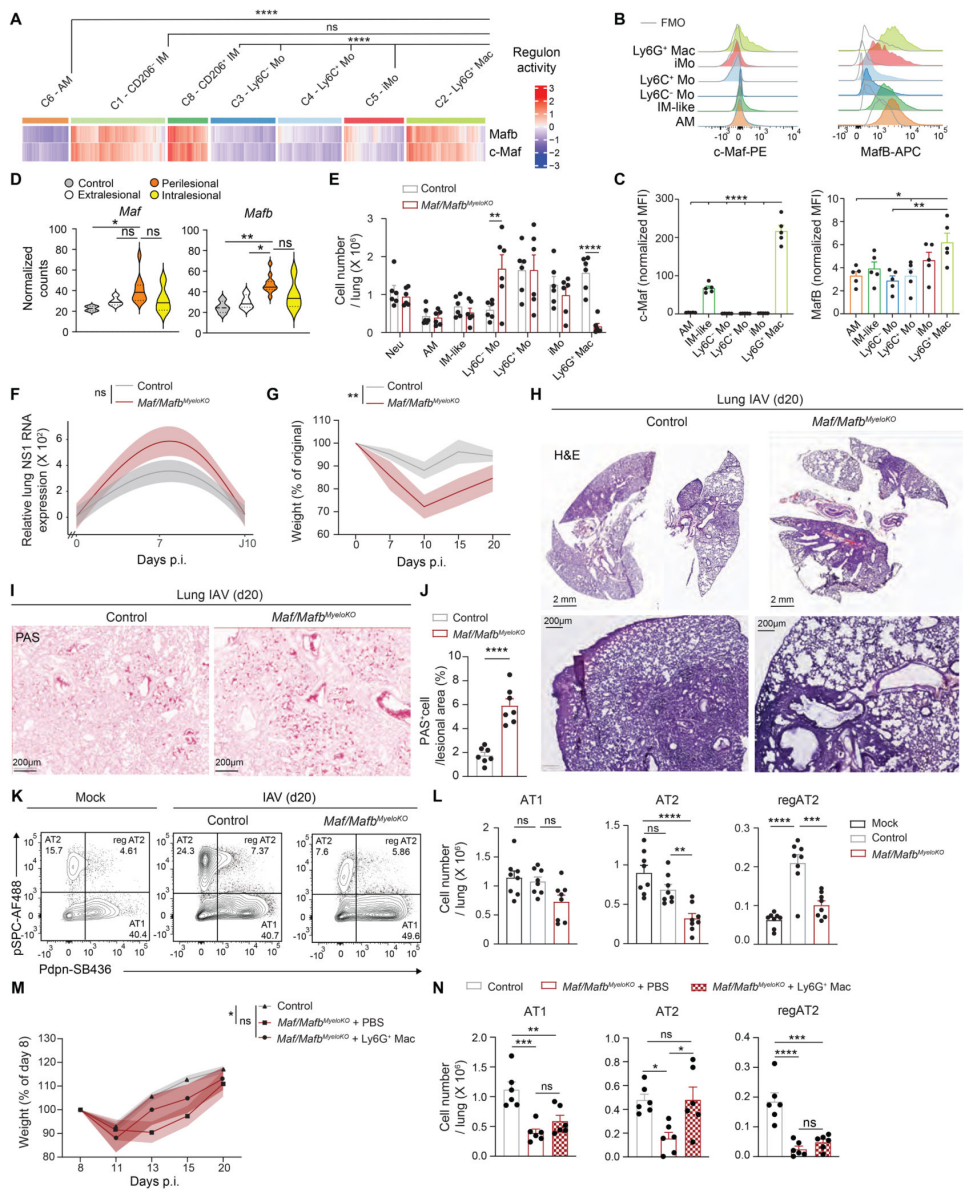
C-Maf/MafB-dependent Ly6G^+^ Macs promote euplastic alveolar epithelial regeneration. (**A**) Heatmap depicting predicted activities of c-Maf and MafB across lung myeloid cells post-IAV, evaluated by SCENIC analysis of the scRNA-seq data, as in Fig. 2A. (**B**) Representative histograms of intracellular c-Maf and MafB expression in the indicated lung myeloid cell populations, quantified at day 10 post-IAV. (**C**) Quantification of expression of intracellular c-Maf and MafB, as in (B). (**D**) Expression of *Maf* and *Mafb* within control, extralesional, perilesional and intralesional ROIs, as depicted by violin plots (height: normalized counts; width: abundance of cells). (**E**) Absolute numbers of the indicated lung myeloid cell populations, quantified at day 10 post-IAV in control and *Maf/Mafb*^MyeloKO^ mice. (**F**) Time course of relative lung NS1 RNA expression, assessed by RT-qPCR at days 0, 7 and 10 post-IAV in control and *Maf/Mafb*^MyeloKO^ mice. (**G**) Time course of weight, expressed as the % of the original weight at day 0 and assessed at days 0, 7, 10, 15, 20 post-IAV in control and *Maf/Mafb*^MyeloKO^ mice. (**H-I**) Representative (H) Hematoxylin & Eosin and (I) Periodic Acid Schiff (PAS, bottom) pictures of lung sections of control and *Maf/Mafb*^MyeloKO^ mice at day 20 post-IAV. Pictures are representative of 1 of 7 mice analyzed. (**J**) Percentage of PAS^+^ cells in lung lesional areas of control and *Maf/Mafb*^MyeloKO^ mice at day 20 post-IAV. (**K**) Representative pSPC and Pdpn contour plots of CD45^-^CD31^-^EpCam^+^ cells in mock- or IAV-infected mice at day 20 post-IAV. (**L**) Absolute numbers of pSPC^+^Pdpn^−^ AT2, pSPC^−^Pdpn^+^ AT1 and pSPC^+^Pdpn^+^ regenerating AT2 (reg AT2), quantified as in (K). (**M**) Time course of weight, expressed as the % of the original weight at day 8 and assessed at days 8, 11, 13, 15 and 20 post-IAV in control mice, in *Maf/Mafb*^MyeloKO^ mice instilled i.t. at days 8, 11, 13 and 15 post-IAV with PBS or with 3 x 10^5^ Ly6G^+^ Macs isolated from WT mice at day 10 post-IAV. (**N**) Absolute numbers of pSPC^+^Pdpn^-^ AT2, pSPC^-^Pdpn^+^ AT1 and pSPC^+^Pdpn^+^ regenerating AT2 (reg AT2), quantified at day 20 post-IAV, as in (M). (C,E,J,L,N) Data show mean + SEM and are pooled from 2 independent experiments (C:*n*=5 mice; E,N: *n*=6 mice/group; J: *n*=7 mice/group; L: *n*=8 mice/group). (F,G,M) Data show mean (centerline) ± SEM (colored area) and are pooled from 2-3 independent experiments (F: *n*=10 mice/group; G: *n*=7 mice/group; M: *n*=6 mice/group). *P* values were calculated using (A) a Wilcoxon rank sum test, (C) a one-way ANOVA with Dunnett’s post hoc tests, (D,L) a one-way ANOVA with Tukey’s post hoc tests, (E,F,G,M) a two-way ANOVA with Sidak’s post hoc tests, (J) a two-tailed Student’s *t* test, *, *P<0.05;* **, *P*<0.01; ***, *P*<0.001; ****, *P*<0.0001. FMO, fluorescence minus one; ns, not significant; p.i., post-infection. Scale bars: (H, top) 2 mm, (H, bottom; I) 200 μm.

To formally assess the function of Ly6G^+^ Macs *in vivo*, we aimed to generate a transgenic mouse strain in which Ly6G^+^ Mac differentiation was impaired. Thus, we applied the SCENIC algorithm to our scRNA-seq data to map gene regulatory networks and predict transcription factor activities in Ly6G^+^ Macs (36). Of note, c-Maf and MafB exhibited a high regulon activity in Ly6G^+^ Macs and IM-like cells, as described (39), but not in other lung myeloid cells (Fig. 6A and fig. S9). Elevated c-Maf and MafB protein levels were also detected in Ly6G^+^ Macs at day 10 post-IAV by flow cytometry (Fig. 6, B and C), and *Maf* and *Mafb* transcript levels were elevated in lung Ly6G^+^ Mac-rich perilesional areas of IAV-infected mice (Fig. 6D). We generated mice with myeloid-restricted *Maf* and *Mafb* deficiency by crossing *Maf* and *Mafb* floxed mice (*Maf/Mafb^fl/fl^*) with mice constitutively expressing Cre recombinase under the control of the lysozyme M promoter (*Lyz2^Cre^*), called *Maf/Mafb^MyeloKO^* mice hereafter. At day 10 post-IAV, *Maf/Mafb^MyeloKO^* mice showed a virtual absence of Ly6G^+^ Macs, while numbers of Neu, AM, IM-like cells, Ly6C^+^ Mo and iMo were similar and numbers of Ly6C^−^ Mo were higher compared to control mice (Fig. 6E). Hence, we employed this model to address the consequences of Ly6G^+^ Mac deficiency on viral control, morbidity and alveolar epithelial repair following IAV infection.

We assessed the levels of lung mRNA coding for the non-structural influenza protein NS1 post-IAV and found that they were not significantly different between *Maf/Mafb^MyeloKO^* and controls and returned to baseline at day 10 post-IAV (Fig. 6F), supporting that Ly6G^+^ Macs did not substantially influence host viral control. However, *Maf/Mafb^MyeloKO^* mice lost more weight post-IAV compared to controls (Fig. 6G), suggestive of a more severe IAV-induced pathology.

Histopathological analyses of lung sections at day 20 post-IAV indicated broader lesional areas in *Maf/Mafb^MyeloKO^* mice compared to controls, as well as more pronounced dysplastic repair and bronchiolization of the alveoli, based on quantification of mucus area in lung lesional areas (Fig. 6H-J). These results suggested that, in the absence of Ly6G^+^ Macs, the classical pathway of alveolar epithelial regeneration involving progenitor AT2 expansion and differentiation towards AT1 (40, 41) was defective and compensated by dysplastic repair. Next, we evaluated the numbers of AT1, AT2 and regenerating AT2 (regAT2) at day 20 post-IAV in *Maf/Mafb^MyeloKO^* and control mice by flow cytometry and observed a significant decrease in the numbers of AT2 and regAT2 in *Maf/Mafb^MyeloKO^* mice compared to controls (Fig. 6, K and L), confirming that AT2 were less able to expand and differentiate into AT1 in the absence of Ly6G^+^ Macs. Of note, i.t. transfer of Ly6G^+^ Macs isolated from lungs of WT mice at day 10 post-IAV into IAV-infected *Maf/Mafb^MyeloKO^* mice partially improved weight recovery and restored numbers of AT2 to the levels observed in IAV-infected control mice (Fig. 6, M and N). These results suggest that Ly6G^+^ Macs are key players of euplastic epithelial regeneration after IAV-induced lung injury.

**Fig. 7:**
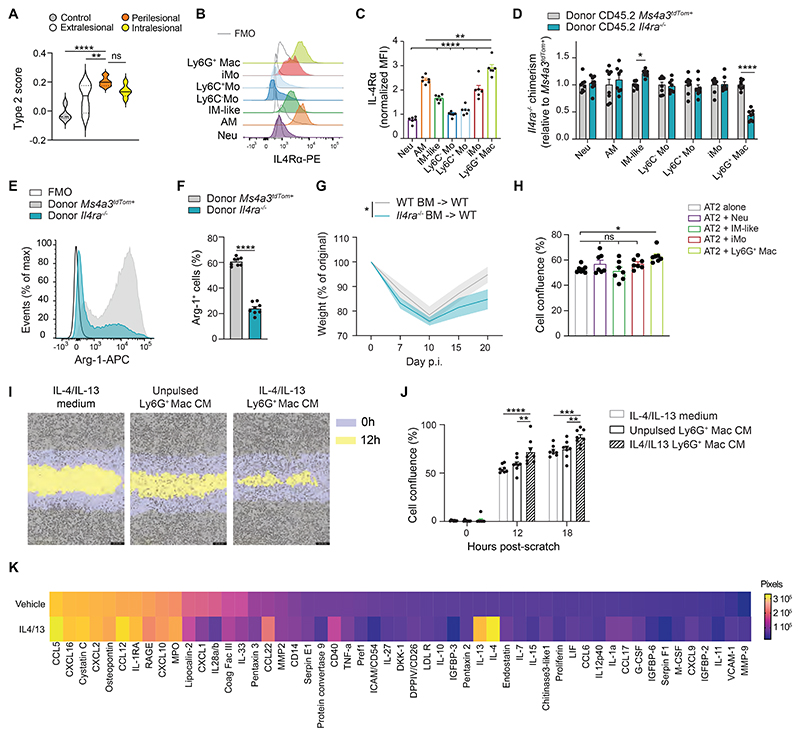
IL-4R-dependent Ly6G^+^ Macs release soluble factors that improve alveolar regeneration from AT2 cells. (**A**) Type 2 signature score within control, extralesional, perilesional and intralesional ROIs, as depicted by violin plots (height: scores; width: abundance of cells). (**B**) Representative histograms of IL-4R expression in the indicated lung myeloid cell populations, quantified at day 10 post-IAV. (**C**) Quantification of IL-4R expression, as in (H). (**D**) Bar graph showing donor *Il4ra*^−/−^ chimerism relative to donor *Ms4a3^tdTom+^* chimerism in the indicated cell populations from lethally-irradiated CD45.1 mice reconstituted with a 1:1 mix of CD45.2 *Il4ra*^−/−^ and *Ms4a3^tdTom+^* BM cells, infected with IAV 4 weeks later and evaluated at day 10 post-IAV. (**E**) Representative histograms and (**F**) quantification of Arg-1^+^ cells (%) in Ly6G^+^ Macs from donor *Il4ra*^−/−^ or *Ms4a3^tdTom+^* BM cells, as in (F). (**G**) Time course of weight, expressed as the % of the original weight at day 0 and assessed at days 0, 7, 10, 15, 20 post-IAV in lethally-irradiated CD45.1/CD45.2 WT mice reconstituted with CD45.2 *Il4ra*^−/−^ BM cells (*Il4ra*^−/−^ BM -> WT) or CD45.2 WT BM cells (WT BM -> WT) and infected with IAV 4 weeks later. (**H**) Cell confluence of AT2 cells (MLE-12) quantified 12 hours after a standardized scratch by live cell analysis when AT2 cells were co-cultured in the presence of Neu, IM-like cells, iMo or Ly6G^+^ Macs isolated from the lungs of WT mice at day 10 post-IAV. (**I**) Representative picture of cell confluence of AT2 cells at 0 and 12 hours post-scratch when AT2 cells were cultured with IL-4/13 or with conditioned medium (CM) of unpulsed or IL-4/13-pulsed Ly6G^+^ Macs isolated from the lungs of WT mice at day 10 post-IAV. (**J**) Bar graph of cell confluence of AT2 cells quantified 0, 12 and 18 hours after scratch, as in (K). (**K**) Heatmap showing the proteome profiling of CM of vehicle and IL-4/13-treated Ly6G^+^ Macs isolated from the lungs of WT mice at day 10 post-IAV. (C,D,F,H,J) Data show mean + SEM and (C,D,F) are pooled from 2 independent experiments (C: *n*=6 mice; D,F: *n*= 8 mice); (H,J) are pooled from 3 independent sorting experiments. (G) Data show mean (centerline) ± SEM (colored area) and are pooled from 2 independent experiments (*n*=6 mice per group). *P* values were calculated using (C) a one-way ANOVA with Dunnett’s post hoc tests, (D,G) a two-way ANOVA with Sidak’s post hoc tests, (F) a two-tailed Student’s *t* test, (H) a one-way or (J) a two-way ANOVA with Tukey’s post hoc tests. *, *P<0.05;* **, *P*<0.01; ****, *P*<0.0001. FMO, fluorescence minus one; ns, not significant; p.i., post-infection.

To determine whether Ly6G^+^ Mac-rich perilesional areas were imprinted by a type 2 reparative environment (19, 42, 43), we mapped a type 2 signature score based on genes involved in IL-4 receptor downstream signaling pathways to the DSP spatial transcriptomic data and found that perilesional areas exhibited the highest type 2 score as compared to the other ROIs (Fig. 7A). Hence, we asked whether IL-4 receptor signaling, whose activation is known to induce a repair phenotype in Macs (19, 42, 43), was involved in Ly6G^+^ Mac identity and function. First, we found that Ly6G^+^ Macs expressed high levels of the IL-4 receptor α chain (IL-4Rα) (Fig. 7, B and C). Next, we generated BM competitive chimeras in which lethally irradiated CD45.1/CD45.2 WT mice were engrafted with a 1:1 mix of CD45.2 *Il4ra*^−/−^ and CD45.2 *Ms4a3^tdTom^* BM cells. At week 4 after reconstitution, efficient BM reconstitution was confirmed in the blood (fig. S10, A and B). At day 10 post-IAV, we found that Ly6G^+^ Macs of donor *Ms4a3^tdTom^* origin exhibited a competitive advantage over those of donor *Il4ra*^−/−^ origin, which was not observed among other lung myeloid cells (Fig. 7D and fig. S10, C and D), and the remaining *Il4ra*^−/−^ Ly6G^+^ Macs were impaired in their ability to express Arg-1 (Fig. 7, E and F). Finally, WT chimeric mice fully reconstituted with *Il4ra*^−/−^ or WT BM cells were generated (*Il4ra*^−/−^ BM -> WT or WT BM -> WT, respectively) and infected with IAV. We found, like in *Maf/Mafb^MyeloKO^* mice, that *Il4ra*^−/−^ BM -> WT mice had an impaired recovery post-IAV compared to WT BM -> WT mice (Fig. 7G). These data suggest that Ly6G^+^ Macs exert their function via IL-4R-dependent pathways, at least in part.

Finally, we asked whether Ly6G^+^ Macs could directly influence AT2 fate and whether cell-cell contacts were needed. To this end, we performed a scratch assay *in vitro* using the MLE-12 mouse AT2 cell line and evaluated the confluence of AT2 cells 12 hours post-scratch in the presence or absence of Ly6G^+^ Macs isolated from infected lung at day 10 post-IAV. Co-culture with Ly6G^+^ Macs, but not with Neu, IM-like cells or iMo was associated with an increase in cell confluence (Fig. 7H), indicating that Ly6G^+^ Macs can directly and specifically promote wound healing in vitro. A similar scratch assay was also performed using conditioned medium (CM) from FACS-sorted Ly6G^+^ Macs that were cultured overnight with or without the type 2 cytokines IL-4 and IL-13. In this setting, CM from IL-4/13-pulsed Ly6G^+^ Macs could promote wound healing compared to control medium (containing only IL-4 and IL-13) or CM from unpulsed Ly6G^+^ Macs (Fig. 7, I and J). We performed proteome profiling on such CM and found that Ly6G^+^ Macs were highly potent in secreting soluble factors, among which were chemokines (CCL5, CXCL16, CCL12, CXCL10), cytokines (TNF-α, IL-10, IL-1α) and osteopontin, some of which were increased upon IL-4R activation (Fig. 7K). Some of the molecules detected in the CM of Ly6G^+^ Macs had their transcript levels significantly upregulated in Ly6G^+^ Macs (C2) as compared to other clusters (fig. S11). Altogether, our data demonstrate that Ly6G^+^ Macs can release soluble factors upon IL-4 receptor triggering that act directly on AT2 to promote epithelial regeneration (fig. S12).

### Ly6G^+^ Macs belong to a conserved host response to injury across organs, triggers and species

**Fig. 8:**
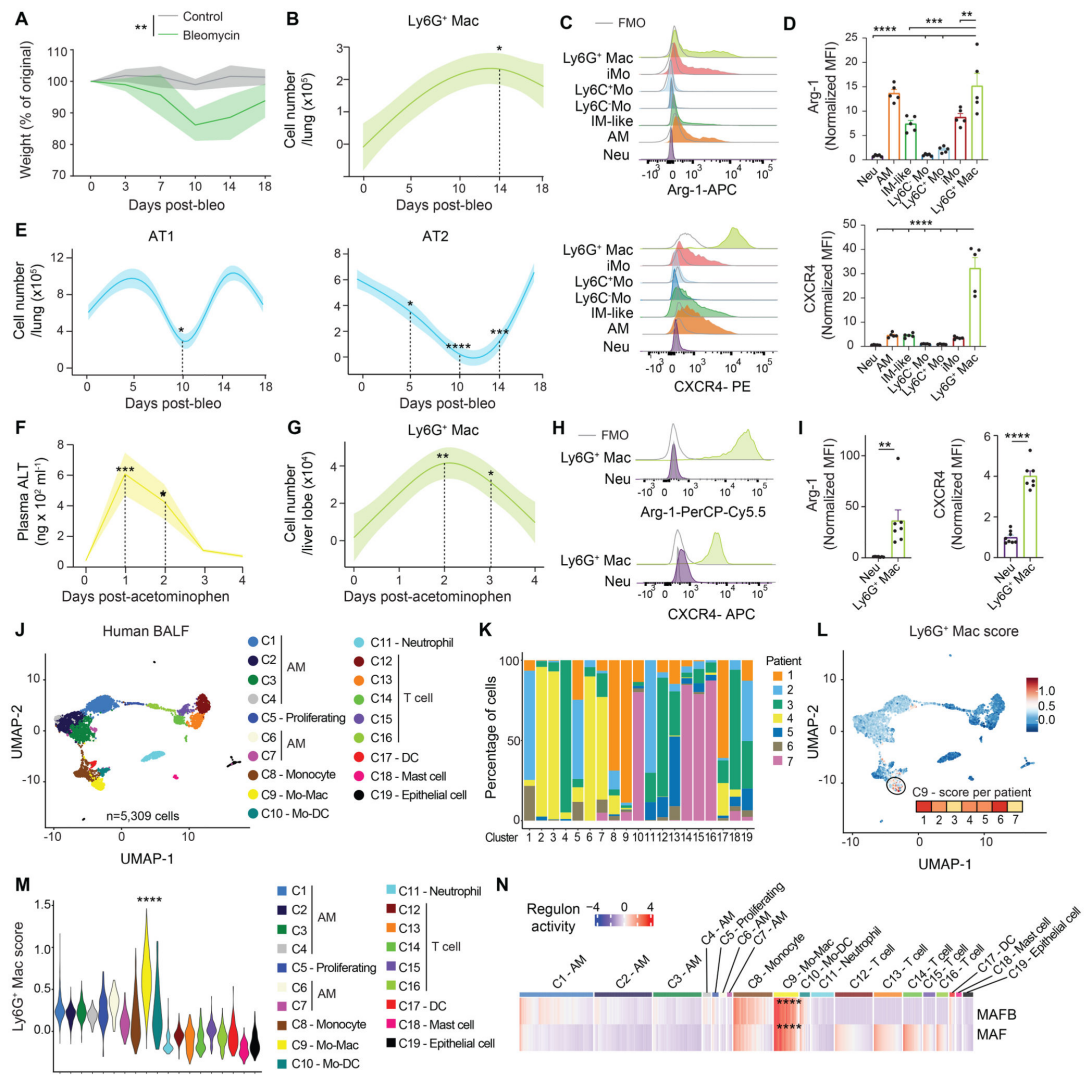
Ly6G^+^ Macs are triggered by other insults and have a human counterpart. (**A**) Time course of weight, expressed as the % of the original weight at day 0 and assessed at days 0, 3, 7, 10, 14 and 18 post-injection in C57BL/6 WT mice instilled i.t. with bleomycin (bleo). (**B**) Time course of absolute numbers of lung Ly6G^+^ Macs quantified at days 0, 5, 10, 14 and 18 post-bleo in WT mice. (**C**) Representative histograms of intracellular Arg-1 and CXCR4 expression in the indicated lung myeloid cell populations, quantified at day 14 post-bleo. (**D**) Quantification of intracellular Arg-1 and CXCR4 expression, as in (C). (**E**) Time course of absolute numbers of pSPC^-^Pdpn^+^ AT1 and pSPC^+^Pdpn^-^ AT2, quantified at days 0, 7, 10, 14 and 18 post-bleo. (**F**) Amount of alanine aminotransferase (ALT) in the plasma of WT mice injected i.p. with acetaminophen at days 0, 1, 2, 3 and 4 post-injection. (**G**) Time course of absolute numbers of liver Ly6G^+^ Macs quantified at days 0, 1, 2, 3 and 4 post-acetominophen. (**H**) Representative histograms of intracellular Arg-1 and CXCR4 expression in liver neutrophils (Neu) and Ly6G^+^ Macs, quantified at day 1 post-acetominophen. (**I**) Quantification of intracellular Arg-1 and CXCR4 expression, as in (H). (**J**) UMAP plot depicting the transcriptional identity of human BALF cells collected from 7 patients suspected of pneumonia and analyzed by scRNA-seq. Annotations of cell clusters are shown. (**K**) Representation of each patient within each cluster, shown as frequency. (**L**) UMAP feature plot, as in (J), according to the Ly6G^+^ Mac signature score. The score level in cluster C9 is shown for each patient. (**M**) Ly6G^+^ Mac signature score of single cells within each cluster, as depicted by violin plots (height: score; width: abundance of cells). (**N**) Heatmap depicting predicted activities of MAFB and MAF across BALF cell populations, evaluated by SCENIC analysis of the scRNA-seq data shown in (**J**). (A,B,E,F,G) Data show mean (centerline) ± SEM (colored area) and are pooled from 2 independent experiments (*n*=5-6 mice). (D,I) Data show mean + SEM and are pooled from 2 independent experiments (*n*=5-7 mice). *P* values were calculated using (A) a two-way ANOVA, (B,D,E,F,G) a one-way ANOVA with Dunnett’s post hoc tests, (I) a two-tailed Student’s *t* test or (M,N) a Wilcoxon rank sum test. (M) *P* values compare C9 vs. all other clusters. *, *P<0.05;* **, *P*<0.01; ***, *P*<0.001; ****, *P*<0.0001. FMO, fluorescence minus one.

We evaluated whether Ly6G^+^ Macs were specifically recruited in the IAV model or were also triggered in other models of injury. First, we used a model of non-infectious lung injury based on bleomycin (bleo) instillation and performed time-course flow cytometry analyses. Ly6G^+^ Macs expressing high levels of Arg-1 and CXCR4 were mostly present between day 7 and day 14 post-bleo, which correlated with signs of epithelial damage, as reflected by the decrease in numbers of AT1 and AT2 (Fig. 8A-E). We also found similar Ly6G^+^ Macs peaking at days 1 and 2 post-treatment in an acute model of acetaminophen-induced liver injury, which correlated with the release of alanine aminotransaminase (ALT) in plasma (Fig. 8F-I). Our data thus suggested that Ly6G^+^ Macs are a component of a conserved response to tissue damage, regardless of the organ or trigger.

Finally, we asked whether Macs sharing a similar transcriptomic signature were also present in the broncho-alveolar lavage fluid (BALF) of diseased humans. We performed scRNA-seq analyses of BALF cells from 7 patients with a suspicion of pneumonia and manually annotated the cell clusters based on the most upregulated genes (Fig. 8, J and K and fig. S13). Next, we mapped a Ly6G^+^ Mac score based on orthologous genes in humans to the BALF cells and found that cells exhibiting the highest Ly6G^+^ Mac score belonged to the same cluster C9 identified as Mo-Macs based on their high expression of monocyte genes and their low expression of AM-associated genes (Fig. 8, L and M). SCENIC analyses (36) predicted higher MAF and MAFB activities in the Mo-Mac cluster compared to other clusters (Fig. 8N), further supporting that the airspace of human pneumonia lungs contains Mo-Macs that are transcriptionally similar to mouse Ly6G^+^ Macs.

## Discussion

Restoration of gas exchanges after lung injury is critical for life and relies on appropriate regulation of inflammation and regeneration of the damaged alveoli. While recent progress has been made in understanding the epithelial-intrinsic mechanisms underlying alveolar regeneration post-injury (3, 38, 41, 44–47), an important gap resides in our understanding of the innate immune-epithelial crosstalk taking place to promote epithelial repair and host recovery. While recruited Mo-Macs are often seen as culprits and drivers of disease progression in different contexts, such as in Covid-19, interstitial fibrosis or lung cancer (13, 17, 18, 48), advances in single cell and spatial technologies have enabled new opportunities to investigate the spatiotemporal regulation of Mo-Mac responses in-depth. Here, using such approaches combined with lineage tracing, BM chimeras, gene targeting, multi-parameter flow cytometry and imaging, our work identifies a previously undescribed atypical population of short-lived recruited Ly6G^+^ Macs that critically contributes to alveolar epithelial regeneration post-injury in mice.

To our knowledge, there is no report of such Ly6G^+^ Macs in the literature. Of note, Ly6G is largely considered as a neutrophil-specific marker (49), and, in many studies employing cytometry, anti-Ly6G antibodies are included to gate out “neutrophils” before gating on Macs. Hence, Ly6G^+^ Macs might have been previously overlooked and considered as part of the neutrophil compartment. Here, we provide evidence that the Ly6G signal is specific and that lung iMo, the precursors of Ly6G^+^ Macs, can actively express Ly6G gene and protein upon GM-CSF stimulation *ex vivo*. Of note, GM-CSF is mainly produced by AT2 cells (41, 50), which are located in the vicinity of Ly6G^+^ Macs, and Ly6G^+^ Macs were dependent on GM-CSF receptor signaling for their generation and Arg-1 expression post-IAV infection *in vivo*. Further supporting an intrinsic upregulation of Ly6G, we found no evidence of Ly6G protein transfer from neutrophils to Ly6G^+^ Macs. In addition to Ly6G expression, Ly6G^+^ Macs were phenotypically and transcriptionally distinct from neutrophils and exhibited key developmental, phenotypic and transcriptomic macrophage features including their dependency on Ccr2, elevated Cx3cr1 expression, and their dependency on the transcription factors c-Maf and MafB. However, *Ly6g* does not appear as a target gene of c-Maf and MafB in the ChiP atlas, suggesting alternative gene regulatory mechanisms.

We found that Ly6G^+^ Macs were transiently recruited to the alveolar spaces of particular lung areas and remained phenotypically and transcriptionally distinct from tissue-resident AM. Our data thus suggest that the local microenvironment of Ly6G^+^ Macs, which can shape Mac identity (13, 51–53), is dynamically regulated and distinct from that of AM. First, we found that Ly6G^+^ Macs originate from BM-derived monocytes recruited to the lung in a Ccr2-dependent manner. Second, we showed that such inflammatory monocytes differentiating into Ly6G^+^ Macs could phagocytose GMP-derived myeloid cells, such as neutrophils, *in vivo*. Interestingly, such a process has been shown to trigger a metabolic rewiring that is associated with Arg-1 activity and aerobic respiration and is important for the resolution of inflammation and tissue repair (54, 55). Third, we provided evidence that GM-CSF and type 2 cytokine signaling through the IL-4 receptor are involved in generation of Ly6G^+^ Macs and their function. While the cellular source of type 2 cytokines remains unknown, a peak of T helper type 2 (Th2) and type 2 innate lymphoid cells (ILC2s) has been reported in lungs of IAV-infected mice around day 10 post-infection (56), a time point that coincides with the peak of Ly6G^+^ Macs. Moreover, Ly6G^+^ Macs can release the Th2-attracting and ILC2-activating signals CCL22 and IL-33, respectively, consistent with the idea that Ly6G^+^ Macs can contribute to the type 2 milieu that promotes their repair phenotype (57–59). Fourth, we found that Ly6G^+^ Macs were spatially-restricted to perilesional areas, zones that were enriched in transitional epithelial cell states involved in AT2-mediated alveolar regeneration (38). Perilesional areas were also sites of intense cytoskeleton activity, aerobic respiration, extracellular matrix deposition and cell migration, all of which are involved in active alveolar epithelial regeneration (3, 9, 41). Our findings are in line with a previous report that identified damaged zones of IAV-infected lungs that were in the periphery of consolidated areas and were sites of active tissue regeneration and AT2 cell proliferation and differentiation (47).

By disrupting myeloid-specific cMaf and MafB-dependent pathways, we obtained *Maf/Mafb^MyleoKO^* mice in which Ly6G^+^ Macs were no longer able to differentiate post-IAV, thus representing a valuable tool to address their functions. We identified Ly6G^+^ Macs as essential actors to license optimal alveolar epithelial regeneration requiring the differentiation of progenitor AT2 cells towards AT1 cells (41). In the absence of Ly6G^+^ Macs, AT2-to-AT1 transitioning cells were virtually absent and a more pronounced dysplastic alveolar repair associated with a bronchiolization of the alveoli was observed. Such epithelial phenotype was associated with exacerbated morbidity and is reminiscent of what is observed in severe forms of respiratory viral infections (3, 41). Additionally, local adoptive transfer of Ly6G^+^ Macs in IAV-infected *Maf/Mafb^MyeloKO^* mice improved weight recovery and restored AT2 numbers to levels seen in IAV-infected control mice, suggesting that Ly6G^+^ Macs could support AT2 expansion post-injury. We further dissected the underlying mechanisms *ex vivo* and found that Ly6G^+^ Macs could directly promote wound healing of murine AT2 cells through IL-4 receptor-mediated release of soluble factors. Among these factors, osteopontin is expressed by Ly6G^+^ Macs and is a ligand for the receptor CD44 (60). Of note, CD44^hi^ AT2 cells represent a subset of AT2 cells with stem cell properties (61), consistent with the hypothesis that the osteopontin-CD44 axis might trigger alveolar regeneration, even though osteopontin release was not potentiated by type 2 cytokine stimulation of Ly6G^+^ Macs under the experimental conditions tested. The elevated Arg-1 expression by Ly6G^+^ Macs could also influence AT2 cells, either via the local deprivation of L-Arginine or the generation of ornithine and polyamines (62, 63). Finally, Ly6G^+^ Macs can also secrete pro-inflammatory cytokines, such as IL-1 and TNF-α, which have been shown to support alveolar regeneration (38, 64).

In the last part of the work, we provided evidence that Macs similar to Ly6G^+^ Macs are part of a host response to injury that is independent of the organ or the initial trigger and that is conserved across species. Indeed, we found that Ly6G^+^ Macs were also recruited in a bleomycin-induced model of non-infectious lung injury and in a model of acute acetaminophen-induced liver injury in mice. Even though Ly6G^+^ Mac numbers were lower in these models compared to the IAV model, it is noteworthy that the peak of Ly6G^+^ Macs correlated with the presence of damage, as attested by the drop in AT1 and AT2 in the lungs or the release of the hepatic enzyme ALT in the liver. These data are consistent with the idea that they may contribute to tissue repair in these models as well, even though it remains to be addressed experimentally. Interestingly, we also reported the presence of transcriptionally similar Macs in the BALF of pneumonia patients by performing scRNA-seq analyses of BALF cells. Of note, cells exhibiting an elevated Ly6G^+^ Mac score belonged to a cluster identified as Mo-Macs, and SCENIC analyses suggested that such Mo-Macs displayed high MAF and MAFB activities, reminiscent of Ly6G^+^ Macs in mice. Speculating that such Mo-Macs also depend on GM-CSF and exhibit similar reparative functions in human lungs, our findings provide a rationale to investigate the benefits of inhaled GM-CSF, beyond the restoration of the AM niche (65), to improve epithelial regeneration after severe viral-induced disorders. Indeed, one could speculate that the fate or functions of Ly6G^+^ Macs are modified and become dysregulated in uncontrolled forms of respiratory viral infections or in chronic fibrotic diseases. Of note, *SPP1*, which encodes osteopontin, has been linked to fibrosis and is often used as a proxy for “pro-fibrotic”, pathogenic Macs (48, 66–68). Our data support that Spp1^+^ Mo-Macs, like Ly6G^+^ Macs, can also exert beneficial roles, while other Spp1^+^ Mo-Macs can become dysregulated, persistent and pathogenic, like in chronic Covid-19 or idiopathic pulmonary fibrosis. Understanding what drives beneficial or pathological responses of *SPP1*^+^ Macs represents an interesting avenue for future research.

No therapeutic options exist to promote lung regeneration so far. By characterizing in-depth a short-lived atypical Mac population that licenses alveolar regeneration post-injury, our findings could serve as a basis to devise novel myeloid-centered regenerative strategies for medically relevant conditions such as severe or chronic respiratory viral infections or acute respiratory distress syndrome.

## Materials and Methods

### Study design

In this study, we investigated the spatio-temporal distribution, transcriptional regulation, fate, identity and function of Ly6G^+^ Macs in an infectious model of lung injury. To this end, we employed flow cytometry, microscopy, single-cell and spatial transcriptomic approaches, bone marrow (BM) chimeras, monocyte fate-mapping and gene targeting. In most of the mouse experiments, 4 to 10 mice per group per time point were used to identify differences between groups with at least 80% power and 5% significance level. In some experiments, no statistical methods were used to pre-determine sample sizes but our sample sizes are similar to those reported in previous publications (39, 69, 74, 75). No statistical methods to pre-determine sample size were used for the analyses of human BALF cells from pneumonia patients. Data from independent experiments were pooled for analysis in each data panel, unless otherwise indicated. No data were excluded from the analyses and all attempts at replication were successful and gave similar results. Histopathological examination of lung sections was blinded. Allocation of animals into experimental groups was done randomly at the start of the experiments. The specific numbers of mice, the number of experimental replicates and the statistical tests performed are all included in the respective figure legends.

### Mice

All experiments, unless otherwise specified, were performed on age-matched 8–12-wk-old male and female mice on the C57BL/6 background. Details about the transgenic strains can be found in the Supplementary Materials. Mice were housed under specific pathogen-free conditions and maintained in a 12-h light–dark cycle with food and water ad libitum. All animal experiments described in this study were carried out in an animal biosafety level 3 containment unit. Experiments were reviewed and approved by the Institutional Animal Care and Use Committee of the University of Liège (ethical approval #2276). The ‘Guide for the Care and Use of Laboratory Animals,’ prepared by the Institute of Laboratory Animal Resources, National Research Council, and published by the National Academy Press, as well as European and local legislations, was followed carefully. Accordingly, the temperature and relative humidity were 21°C and 45-60%, respectively

### *In vivo* models of injury

The mouse-adapted influenza strain A/Puerto Rico/8/34 (H1N1; PR8) was kindly provided by F. Trottein (Institut Pasteur, France). The viral stock suspension (10^8^ Plaque Forming Units [PFU] ml^–1^) was diluted and 5 PFU were administered intranasally (i.n.) to isoflurane-anesthetized mice in 50 μl of PBS (Thermo Fisher). Control groups received an equal volume of PBS i.n. for mock infection.

For bleomycin-induced lung injury, isoflurane-anesthetized mice were treated intratracheally (i.t.) with a single instillation of 0.06 IU of bleomycin (Bio-Connect) in a volume of 50μl PBS. Control animals received 50μl PBS alone.

For acetaminophen-induced liver injury, mice were fasted during 15 hours with free access to water and were injected intraperitoneally (i.p.) with 300mg kg^-1^ of acetaminophen (Sigma) in saline solution (NaCl 0.9%). Free access to food was allowed after treatment.

### Reagents and antibodies

A complete list of the reagents and antibodies used in this manuscript can be found in Tables S2 and S3, respectively.

### Flow cytometry

Staining reactions were performed in the dark at 4°C for 30 minutes with 2% v/v of Fc block (BD Biosciences) to avoid nonspecific binding. For intracellular staining, extracellular-stained cells were fixed and permeabilized with the Foxp3/Transcription factor Staining Buffer Set (Thermo Fisher). For EdU staining, extracellular-stained cells were permeabilized and stained using Click-iT EdU Alexa Fluor 488 Flow Cytometry Assay Kit (Thermo Fisher), according to the manufacturer’s instructions.

Cell viability was assessed using 7-AAD (BD Bioscience) or Fixable Viability Dye eFluor™ 780 (Thermo Fisher). Cell suspensions was analysed with a FACSCANTO II or a LSRFortessa (BD Biosciences). Results were analyzed using FlowJo software (Tree Star). For scRNA-seq, transmission electron microscopy, cytological examination and *ex vivo* experiments, lung myeloid cells were sorted using a FACSAria III (BD Biosciences) or a Sony MA900.

### *In vivo* treatments

For EdU incorporation experiments shown in fig. S3, B and C, mice were injected i.p. at day 10 post-IAV with 1 mg EdU (Santa Cruz Biotechnology) in 200 μl PBS 4 hours before sacrifice. For experiments addressing the lifespan of Ly6G^+^ Macs (Fig. 3G), 1mg EdU in 200 μl PBS was injected i.p. twice 5 hours apart at day 7 post-IAV, and EdU incorporation was evaluated in blood leucocytes at day 8 post-IAV. The incorporation of EdU in lung myeloid cells was evaluated at days 10, 14 and 17 post-IAV. Assessment of phagocytic activity was performed as previously described (69). Briefly, isoflurane-anesthetized mice were instilled i.t. with 2.10^8^ pHrodo™ Green E. coli BioParticules (Thermo Fisher) in 100 μl PBS. Lungs were harvested 3 hours later for flow cytometry analyses.

### Generation of BM (competitive) chimeras

CD45.2, CD45.1 or CD45.1/CD45.2 WT mice were anesthetized by i.p. injection of 200 μl PBS containing ketamine (Nimatek, Dechra, 75 mg kg^-1^) and xylazine (Rompun, Bayer, 10 mg kg^-1^). When mentioned, the thoracic cavity was protected with a 0.6-cm-thick lead cover. Mice were irradiated with two consecutive doses of 6 Gy 15 minutes apart. Once recovered from the anaesthesia, mice were reconstituted by intravenous (i.v.) administration of 2.10^6^ BM cells from *Ms4a3^tdtom^* or *Il4ra*^−/−^ mice, for full chimeras. For mixed BM chimeras, mice were reconstituted i.v. with 2.10^6^ BM cells consisting of a 1:1 mix of BM cells obtained from the following mice: CD45.1 WT, *Ms4a3^tdtom^*, *Ccr2*^−/−^, CD45.1 *Csf2ra*^−/−^, CD45.2 *Csf2ra^+/+^*, *Cx3cr1*^GFP+^, *Il4ra*^−/−^, or homozygous *Ly6g^CreERT2^* mice (also called *Ly6g*^−/−^ mice). From the day of irradiation, mice were treated for 4 weeks with 0.05 mg ml^−1^ of enrofloxacin (Baytril, Bayer) in drinking water. Chimerism was assessed by flow cytometry in the blood 4 weeks after irradiation.

### scRNA-sequencing and analyses

For mouse experiments, lung myeloid cells were FACS-sorted as living singlet CD45^+^, F4/80^+^ and/or CD11b^+^ cells from lung single-cell suspensions pooled from 5 mock-infected and IAV-infected C57BL/6 male WT mice at day 10 post-IAV. For each sample, an aliquot of Trypan blue-treated cells was examined under the microscope for counting, viability and aggregate assessment following FACS sorting. Viability was above 90% for all samples and no aggregates were observed. Cell preparations were centrifuged and pellets were resuspended in calcium- and magnesium-free PBS containing 0.4 mg ml^−1^ UltraPure BSA (Thermo Fisher Scientific). The 10X Genomics platform (Single Cell 3’ Solution) was used. For library preparation, approximately 2,000 (Mock group) and 6,000 (IAV group) cells were loaded into the Chromium Controller, in which they were partitioned, their polyA RNAs captured and barcoded using Chromium Single Cell 3’ GEM, Library & Gel Bead Kit v3 (10X Genomics). The cDNAs were amplified and libraries compatible with Illumina sequencers were generated using Chromium Single Cell 3’ GEM, Library & Gel Bead Kit v3 (10X Genomics).

For human BALF cell analyses, chromium Fixed RNA Profiling for multiplexed samples (10X Genomics) was used for scRNA-seq analysis of human BALF cells, allowing the storage of fixed cells and enabling analysis of multiple samples in one single GEM reaction. Fresh samples were directly fixed in a 4% formaldehyde solution after collection for storage at -80°C. For GEM creation, the Multiplex-compatible Chromium Next GEM Single Cell Fixed RNA Human Transcriptome Probe Kit including a Probe Barcode that permits sample multiplexing and subsequent demultiplexing was used.

Details about scRNA-seq analyses can be found in the Supplementary Materials.

### Transmission electron microscopy

FACS-sorted myeloid cell populations or lung tissues from IAV-infected mice at day 10 post-IAV were fixed in 2.5% glutaraldehyde (diluted in Sorensen’s buffer: 0.1 M Na2HPO4/NaH2PO4 buffer, pH 7.4) for 1h at 4 °C and postfixed for 30 min in 2% OsO4 (diluted in 0.1 M Sorensen’s Buffer). After dehydration in graded ethanol, samples were embedded in Epon resin. Ultrathin sections obtained with a Reichert Ultracut S ultramicrotome (Reichert Technologies) were contrasted with 2% uranyl acetate and 4% lead citrate. For ultrastructural analyses, random fields of cells were examined under a Jeol TEM JEM-1400 Transmission Electron Microscope at 80 kV, and photographed using an 11-megapixel camera system (Quemesa, Olympus).

### Extracellular flux analysis

Oxygen consumption rate (OCR) was measured using Seahorse XF Cell Mito Stress Test (Agilent) according to manufacturer’s recommendations and as described previously (70, 71). Briefly, Neu, IM-like cells and Ly6G^+^ Macs were FACS-sorted at day 10 post-IAV and seeded (10.10^4^, 7.10^4^ and 8.10^4^ cells/well, respectively) in XFp mini-plates (Agilent) pre-coated with CellTak. Cells were kept in unbuffered serum-free DMEM supplemented with pyruvate (1mM), glutamine (2mM), glucose (10mM), at pH 7.4, 37 °C and ambient CO_2_ for 1h before the assay. Analysis was performed using the XFp analyser (Seahorse Bioscience) as per manufacturer’s instructions. Additional details can be found in the Supplementary Materials.

### Spatial transcriptomic analyses using Digital Spatial Profiling (DSP)

Five-μm-thick formalin-fixed, paraffin-embedded (FFPE) sections were prepared using the protocol from NanoString Technologies. Briefly, 2 tissue slides, each containing 1 mock and 2 IAV samples harvested 10 days post-IAV, were analyzed. Slides were first stained with antibodies against CD68, Ly6G (clone 1A8), and DNA was visualized with 500 nM Syto83. Mouse Whole Transcriptome Atlas probes targeting more than 19,000 targets were hybridized, and slides were loaded on the GeoMx DSP. Briefly, entire slides were imaged at x20 magnification, and Regions of Interest (ROIs) were chosen based on serial Hematoxylin & Eosin sections and on morphological markers to select lesional, perilesional and extralesional areas. ROIs were exposed to ultraviolet light, releasing the indexing oligos and collecting them in a 96-well plate for subsequent processing and sequencing, as described (72). Raw count, third quartile (Q3)− normalized count data of target genes from ROIs were provided by the vendor, which were used as input to downstream analyses. Additional details can be found in the Supplementary Materials.

### Immunofluorescence

Immunofluorescence staining of mouse lungs were performed as previously described (39). Briefly, lungs from WT or *Cx3cr1^GFP+^* mice were perfused with 5 ml PBS through the right ventricle then with 5 ml paraformaldehyde (PFA) 4% (Thermo Fisher) in PBS, and lungs were collected. Lungs were fixed for 4 h in 4% PAF at 4 °C, then cryoprotected overnight in 30% sucrose (VWR) in PBS at 4 °C, followed by embedding in optimal cutting temperature compound (OCT) (VWR) and stored at −80 °C. Seven-μm-thick sections were cut and left in a methanol 100% (Merck) bath at −20 °C for 20 minutes prior to be stained. Additional details can be found in the Supplementary Materials. All images were acquired on an LSM 980 with Airyscan 2 inverted confocal microscope (Zeiss) using a LD C-Apochromat ×40/1.1 W objective and Zen Black software. Additional details can be found in the Supplementary Materials.

### *Ex vivo* experiments

For *ex vivo* stimulation and co-culture experiments, single cell suspensions isolated from IAV-infected lungs of WT or *Ly6g^tdTom^* mice at day 10 post-IAV were enriched in CD11b^+^ cells by a magnetic-activated cell sorting (MACS) using CD11b MicroBeads (Myltenyi). Cells were then stained and FACS-sorted using the gating strategy shown in Fig. 1A. After sorting, cells were counted, spinned down, and either directly added to the co-culture with MLE-12 cells, or seeded in 96 wells at a concentration 5.10^4^ cells/well in complete RPMI (ThermoFischer), containing 1mM sodium pyruvate, 1% vol/vol MEM non-essential amino acids, 50 U ml^−1^ Penicillin-Streptomycin and 10% vol/vol FBS. For stimulation experiments, recombinant mouse GM-CSF (20 ng ml^-1^, Peprotech), mouse M-CSF (20 ng ml^-1^, Peprotech), mouse IL-4 (20 ng ml^-1^, Peprotech) or mouse IL-13 (20 ng ml^-1^, Peprotech) were added. When required, Cre-ERT2 activation was achieved by adding 0.02 mg ml^-1^ of 4-hydroxytamoxifen (Sigma-Aldrich). After 18 hours of culture, cell supernatants were collected (conditioned medium, CM) and cells were harvested for flow cytometry phenotyping.

Murine lung epithelial (MLE)-12 cells (ATCC, CVCL_3751) were used. Scratch Wound Assay were performed using IncucyteS3 (Sartorius). MLE-12 cells were seeded in 96-well (Sartorius) at density of 4.10^4^ cells/well and incubated 24 hours in DMEM/F12 medium. An open wound area was created in the cell monolayer using the IncuCyte ® Wound Maker tool, and subsequently co-cultured with FCAS-sorted cells or incubated with CM from unpulsed or IL-4/IL-13-pulsed Ly6G^+^ Macs.

Additional details can be found in the Supplementary Materials.

### Adoptive transfer of Ly6G^+^ Macs *in vivo*

Ly6G^+^ Macs were isolated from the lungs of CD45.2 WT mice at day 10 poist-IAV. Lung single cell suspensions were first enriched in CD11b^+^ cells by MACS using CD11b MicroBeads (Miltenyi Biotec) and were FACS-sorted using a Sony MA900. Four hundred thousand (4 x 10^5^) Ly6G^+^ Macs were resuspended in 50 μl sterile PBS and were instilled i.t. in lightly isoflurane-anesthetized *Maf/Mafb^MyeloKO^* mice at days 8, 11, 13, 15 post-IAV. Control *Maf/Mafb*^MyeloKO^ mice and WT mice received 50 μl PBS as vehicle.

### Human BALFs

The use of human BALF cells was approved in 2022 by the Ethics Reviewing Board of the University Hospital of Liege, Belgium (ref. 2022/159). The characteristics of the patients are summarized in Table S1. Human BALFs were fixed directly after collection for storage and scRNA-seq analyses.

### Statistical analysis

Graphs were prepared with GraphPad Prism 9 (GraphPad software) or R Bioconductor (3.5.1) (73). Data distribution was assumed to be normal when parametric tests were performed. Data from independent experiments were pooled for analysis in each data panel, unless otherwise indicated. No data were excluded from the analyses. Statistical analyses were performed with Prism 9 (GraphPad software), and with R Bioconductor (3.5.1) (73) and Seurat (76) for scRNA-seq data, respectively. The statistical analyses performed for each experiment are indicated in the respective figure legends. We considered a *P* value lower than 0.05 to be significant (*, *P* < 0.05; **, *P* < 0.01; ***, *P* < 0.001; ****, *P* < 0.0001; ns, not significant).

Additional sections and details about Materials and Methods can be found in the Supplemental Materials.

## Supplementary Material

Supplementary Materials

## Data Availability

*Ms4a3^Cre^* mice are used under the terms of a material transfer agreement (MTA) signed between Singapore Immunology Network (SIgN), A*STAR Research Entities (Singapore) and the University of Liege (Belgium). *Ly6g^CreERT2^* mice are used under the terms of an MTA signed between Centro Nacional de Investigaciones Cardiovasculares Carlos III (CNIC, Madrid, Spain), and the University of Liege (Belgium). Mouse and human single-cell RNA-seq data have been deposited at GEO and are available under GEO accession GSE244765. All original codes have been deposited at Zenodo and are available via this link: https://zenodo.org/records/11354523. Requests for *Mab^fl/fl^* mice should be addressed to t.marichal@uliege.be and will be shared via an MTA.

## One-Sentence Summary

Short-lived perilesional Ly6G^+^ macrophages license alveolar epithelial regeneration after viral-triggered lung injury.
