# Supplementary material for: Recruited atypical Ly6G+ macrophages license alveolar regeneration after lung injury
Source: Sci Immunol. Author manuscript; Available in PMC 2024 Sep 7. (PMC7616420; doi:10.1126/sciimmunol.ado1227)
Supplement: Supplementary Materials [file EMS198347-supplement-Supplementary_Materials.pdf]

Supplementary Materials for

Recruited atypical Ly6G$^+$ macrophages license alveolar regeneration after lung injury

C. Ruscitti[1,2], J. Abinet[1,2], P. Maréchal[1,2], M. Meunier[1,2], C. de meeûs[2,3], D. Vanneste[1,2], P. Janssen[1,2], M. Dourcy[2,4], M. Thiry[5], F. Bureau[2,6], C. Schneider[7], B. Machiels[2,4], A. Hidalgo[8,9], F. Ginhoux[10,11,12,13], B.G. Dewals[2,4], J. Guiot[14,15], F. Schleich[14,15], M-M. Garigliany[2,3], A. Bellahcène[16], C. Radermecker[1,2,*], T. Marichal[1,2,17,*]

Corresponding authors: c.radermecker@uliege.be; t.marichal@uliege.be

The PDF file includes:

Materials and Methods
Fig. S1. Morphology and phenotype of lung myeloid cells at day 10 post-IAV.
Fig. S2. Gene expression in and features of myeloid cell clusters identified by scRNA-seq at day 10 post-IAV.
Fig. S3. Ly6G$^+$ Macs do not proliferate, are short-lived and arise from recruited BM-derived monocytes post-IAV.
Fig. S4. Specificity of the anti-Ly6G staining and analysis of IAV-infected *Csf2ra*$^{-/-}$: *Csf2ra*$^{+/+}$ mixed BM chimeras.
Fig. S5. Transcriptomic comparison of Ly6G$^+$ Macs and SatM monocytes.
Fig. S6. Efferocytic abilities of lung myeloid cells post-IAV.
Fig. S7. Ly6G$^+$ Macs cluster with regenerating AT2 in perilesional areas post-IAV.
Fig. S8. Identification of Ly6G$^+$ Macs-AT2 clusters by confocal microscopy in lung perilesional areas post-IAV.
Fig. S9. SCENIC analysis of lung myeloid cells at day 10 post-IAV.
Fig. S10. Analysis of IAV-infected *Il4ra*$^{-/-}$:*Il4ra*$^{+/+}$ mixed BM chimeras.
Fig. S11. Gene expression in myeloid cell clusters identified by scRNA-seq at day 10 post-IAV.
Fig. S12. Proposed model of Ly6G$^+$ Mac-mediated alveolar epithelial regeneration after IAV-triggered injury.
Fig. S13. Transcriptomic identities of human BALF single cells analyzed by scRNA-seq.
Table S1. Characteristics of patients from whom originate the BALF cells analyzed by scRNA-seq.
Table S2. List of reagents used in this study.
Table S3. List of antibodies used in this study.

Materials and Methods

Mice

All experiments, unless otherwise specified, were performed on age-matched 8–12-wk-old male and female mice on the C57BL/6 background. The following strains of mice were used: CD45.2 wild-type (WT) C57BL/6J (The Jackson Laboratory, #000664); CD45.1 WT (The Jackson Laboratory, #002014); *Cx3cr1*$^{GFP/+}$ (*77*) (The Jackson Laboratory, #005582); *Ccr2*$^{-/-}$ (*78*) (The Jackson Laboratory, #004999); *Ms4a3*^{Cre} (*31*); *Ly6g*^{CreERT2} (*37*); *R26*$^{LSL\text{tdTomato}}$ (*79*) (The Jackson Laboratory, #007909); *Maf*$^{fl/fl}$ (*80*), *Mafb*$^{fl/fl}$ (*39*); *Lyz2*^{Cre} (*81*) (The Jackson Laboratory, #004781); *Il4ra*$^{-/-}$*;* CD45.1 *Csf2ra*$^{-/-}$ (*82*). *Il4ra*$^{-/-}$ mice were kindly provided by Bernhard Ryffel (CNRS Orléans, France). CD45.1/CD45.2 WT C57BL/6J mice were obtained from CD45.1 WT crossed with CD45.2 WT mice. Myeloid-restriced c-Maf and Mafb depletion was achieved by crossing *Maf*$^{fl/fl}$ and *Mafb*$^{fl/fl}$ mice with Lyz2^{Cre} mice. *Ms4a3*^{tdTom} and *Ly6g*^{tdTom} mice were obtained by crossing *R26*$^{LSL\text{tdTomato}}$ with *Ms4a3*^{Cre} and *Ly6g*^{CreERT2} mice, respectively.

Mice were housed under specific pathogen-free conditions and maintained in a 12-h light–dark cycle with food and water ad libitum. All animal experiments described in this study were carried out in an animal biosafety level 3 containment unit. Experiments were reviewed and approved by the Institutional Animal Care and Use Committee of the University of Liège (ethical approval #2276). The 'Guide for the Care and Use of Laboratory Animals,' prepared by the Institute of Laboratory Animal Resources, National Research Council, and published by the National Academy Press, as well as European and local legislations, was followed carefully. Accordingly, the temperature and relative humidity were 21°C and 45-60%, respectively

In vivo models of injury

The mouse-adapted influenza strain A/Puerto Rico/8/34 (H1N1; PR8) was kindly provided by F. Trottein (Institut Pasteur, France). The viral stock suspension (10^8 Plaque Forming Units [PFU] ml^{-1}) was diluted and 5 PFU were administered intranasally (i.n.) to isoflurane-anesthetized mice in 50 µl of PBS (Thermo Fisher). Control groups received an equal volume of PBS i.n. for mock infection.

For bleomycin-induced lung injury, isoflurane-anesthetized mice were treated intratracheally (i.t.) with a single instillation of 0.06 IU of bleomycin (Bio-Connect) in a volume of 50µl PBS. Control animals received 50µl PBS alone.

For acetaminophen-induced liver injury, mice were fasted during 15 hours with free access to water and were injected intraperitoneally (i.p.) with 300mg kg^{-1} of acetaminophen (Sigma) in saline solution (NaCl 0.9%). Free access to food was allowed after treatment.

Assessment of viral NS1 mRNA levels

Whole lungs were excised and total mRNA was isolated from homogenized tissues according to the Immgen protocol (www.immgen.org). cDNA was obtained with RevertAid First Strand cDNA Synthesis Kit (Thermo Fisher), and qPCR was performed in duplicate with iTaq Universal SYBR green supermix (BioRad). Primer sequences were as follows: 5′-TTCACCATTGCCTTCTCTTC-3′ and 5′-CCCATTCTCATTACTGCTTC-3′ for viral NS1, 5′-CATGGCTCGCTCGGTGACC-3′ and 5′-AATGTGAGGCGGGTGGAACTG-3′ for housekeeping gene B2m. Expression levels of NS1 were normalized relative B2M control gene.

Reagents and antibodies

A complete list of the reagents and antibodies used in this manuscript can be found in Tables S2 and S3, respectively.

Bone marrow, blood and tissue cell isolation

Cell isolation was achieved as previously described (*39*, *74*). Briefly, for BM cells, femurs were dissected and cleaned of soft adhering tissue. Distal and proximal ends were opened, and BM cells were flushed out. After centrifugation, cell pellets were re-suspended in ice-cold PBS containing 10 mM EDTA and cell suspensions were filtered using a cell strainer (70 µM, Corning) to obtain a single cell suspension. Blood was collected from the tail vein in a 100 mM EDTA (Merck Millipore)-containing tube, and red blood cells were lysed with RBC lysis buffer (ThermoFischer). For the isolation of lung leucocytes and structural cells, lung vessels were perfused with 5 ml PBS through the right ventricle and 1 ml HBSS (Lonza) containing 0.5 UI dispase II (Sigma-Aldrich), 0.1mg ml^{-1} elastase (MedChemExpress), 0.075 mg ml^{-1} DNAse (Roche) was injected i.t. before dissecting the lung and digesting it for 30 minutes at 37°C in the same digestion medium. After 30 minutes of digestion, lungs were cut into small pieces with razor blades and further digested for 30 minutes at 37°C in HBSS containing 5% vol/vol FBS (Thermo Fisher), 0.5 UI dispase II, 0.05 mg ml^{-1} DNAse, 1 mg ml^{-1} collagenase A (Roche). After 30 minutes, the cell suspension was flushed using a 18-gauge needle to dissociate aggregates. Ice-cold PBS containing 10 mM EDTA was added to stop the digestion process and cell suspensions were filtered using a 70 µm cell strainer. Residual red blood cells were lysed with RBC lysis buffer. Leucocytes isolation from the liver was obtained as previously described (*83*). Briefly, after sacrifice, mice were perfused with 10 ml PBS through right ventricle, the liver middle lobe was dissected and the gallbladder removed. The liver was cut into small pieces with razor blades and digested for 45 minutes at 37°C in 10 ml HBSS containing 0.2mg ml^{-1} collagenase IV (ThermoFisher), 5U ml^{-1} DNase I and 10% vol/vol FBS (Thermo Fischer). After incubation, the homogenized liver suspension was filtered using a 70 µm cell strainer, washed with 10 ml ice-cold PBS containing 10mM EDTA and centrifuged at 50 rcf for 3 minutes. The aqueous phase was recovered, filtered in a new tube and centrifuged at 400 rfc for 5 minutes. The cell pellet was suspended in RBC lysis buffer for 2 minutes, then washed with 10 ml ice-cold PBS containing 10mM EDTA. In all experiments, the number of cells was counted using an automatic cell counter (iPrasense Norma XS).

Flow cytometry

Staining reactions were performed in the dark at 4°C for 30 minutes with 2% v/v of Fc block (BD Biosciences) to avoid nonspecific binding. For intracellular stainings, extracellular-stained cells were fixed and permeabilized with the Foxp3/Transcription factor Staining Buffer Set (Thermo Fisher). For EdU stainings, extracellular-stained cells were permeabilized and stained using Click-iT EdU Alexa Fluor 488 Flow Cytometry Assay Kit (Thermo Fisher), according to the manufacturer's instructions.

Cell viability was assessed using 7-AAD (BD Bioscience) or Fixable Viability Dye eFluor™ 780 (Thermo Fisher). Cell suspensions was analysed with a FACSCANTO II or a LSRFortessa (BD Biosciences). Results were analyzed using FlowJo software (Tree Star). For scRNA-seq, transmission electron microscopy, cytological examination and *ex vivo* experiments, lung myeloid cells were sorted using a FACSAria III (BD Biosciences) or a Sony MA900.

Cytologic examination

Cytologic examination of FACS-sorted neutrophils, iMo, IM-like cells, iMo and Ly6G$^+$ Macs at 10 days post-IAV was performed on cytospin preparations stained with Hemacolor (Merck KgaA). Sections were examined with an Echo Revolve microscope.

Annexin V/Propidium iodide assay

Annexin V/Propidium iodide (PI) apoptosis assay was performed as described (*84*). Briefly, after lung cell isolation, cells were resuspended in Annexin V binding buffer (ThermoFisher) and 2.10^6 cells were stained with Annexin V-APC and incubated in the dark for 15 minutes at room temperature. Cells were washed with 100 µl of Annexin V binding buffer and 2 µg ml^{-1} PI (ThermoFisher) were added to each sample and incubated in the dark for 15 minutes at room temperature. After washing with 500µl of Annexin V buffer, cells were centrifuged, cell pellet was resuspended in fixative solution of 1% vol/vol formaldehyde in PBS and incubated for 10 minutes on ice. Cells were washed with PBS, centrifuged, resuspended in PBS supplemented with 50 µg ml^{-1} RNase A (Merck Millipore) and incubated for 15 minutes at 37°C. Cell suspension was washed with PBS, centrifuged and resuspended for flow cytometry stainings.

In vivo treatments

For EdU incorporation experiments shown in fig. S3, B and C, mice were injected i.p. at day 10 post-IAV with 1 mg EdU (Santa Cruz Biotechnology) in 200 µl PBS 4 hours before sacrifice. For experiments addressing the lifespan of Ly6G$^+$ Macs (Fig. 3G), 1mg EdU in 200 µL PBS was injected i.p. twice 5 hours apart at day 7 post-IAV, and EdU incorporation was evaluated in blood leucocytes at day 8 post-IAV. The incorporation of EdU in lung myeloid cells was evaluated at days 10, 14 and 17 post-IAV. Assessment of phagocytic activity was performed as previously described (*69*). Briefly, isoflurane-anesthetized mice were instilled i.t. with 2.10^8 pHrodo™ Green E. coli BioParticules (Thermo Fisher) in 100 µl PBS. Lungs were harvested 3 hours later for flow cytometry analyses.

Generation of BM (competitive) chimeras

CD45.2, CD45.1 or CD45.1/CD45.2 WT mice were anesthetized by i.p. injection of 200 µl PBS containing ketamine (Nimatek, Dechra, 75 mg kg^{-1}) and xylazine (Rompun, Bayer, 10 mg kg^{-1}). When mentioned, the thoracic cavity was protected with a 0.6-cm-thick lead cover. Mice were irradiated with two consecutive doses of 6 Gy 15 minutes apart. Once recovered from the anaesthesia, mice were reconstituted by intravenous (i.v.) administration of 2.10^6 BM cells from *Ms4a3^{tdtom}* or *Il4ra$^{-/-}$* mice, for full chimeras. For mixed BM chimeras, mice were reconstituted i.v. with 2.10^6 BM cells consisting of a 1:1 mix of BM cells obtained from the following mice: CD45.1 WT, *Ms4a3^{tdtom}*, *Ccr2$^{-/-}$*, CD45.1 *Csf2ra$^{-/-}$*, CD45.2 *Csf2ra$^{+/+}$*, *Cx3cr1*$^{GFP+}$, *Il4ra$^{-/-}$*, or homozygous *Ly6g^{CreERT2}* mice (also called *Ly6g$^{-/-}$* mice). From the day of irradiation, mice were treated for 4 weeks with 0.05 mg ml^{-1} of enrofloxacin (Baytril, Bayer) in drinking water. Chimerism was assessed by flow cytometry in the blood 4 weeks after irradiation.

scRNA-sequencing and analyses

Mouse scRNA-seq analyses

Lung myeloid cells were FACS-sorted as living singlet CD45$^+$, F4/80$^+$ and/or CD11b$^+$ cells from lung single-cell suspensions pooled from 5 mock-infected and IAV-infected C57BL/6 male WT

mice at day 10 post-IAV. For each sample, an aliquot of Trypan blue-treated cells was examined under the microscope for counting, viability and aggregate assessment following FACS sorting. Viability was above 90% for all samples and no aggregates were observed. Cell preparations were centrifuged and pellets were resuspended in calcium- and magnesium-free PBS containing 0.4 mg ml^{-1} UltraPure BSA (Thermo Fisher Scientific).

The 10X Genomics platform (Single Cell 3' Solution) was used. For library preparation, approximately 2,000 (Mock group) and 6,000 (IAV group) cells were loaded into the Chromium Controller, in which they were partitioned, their polyA RNAs captured and barcoded using Chromium Single Cell 3' GEM, Library & Gel Bead Kit v3 (10X Genomics). The cDNAs were amplified and libraries compatible with Illumina sequencers were generated using Chromium Single Cell 3' GEM, Library & Gel Bead Kit v3 (10X Genomics). The libraries were sequenced on an Illumina NovaSeq sequencer on an SP100 cell flow (Read1: 28 cy, read2: 76 cy, index1: 10cy, index2: 10cy) at a depth of 50,000 reads per cell.

The Cell Ranger (v6.1.2) application (10x Genomics) was used to demultiplex the BCL files into FASTQ files (cellranger mkfastq), to perform alignment (to Cell Ranger mouse genome references 6.1.2 GRCm38/release 102), filtering and unique molecular identifier counting and to produce gene-barcode matrices.

Filtered matrix files were used for further scRNA-seq analyses with R Bioconductor (3.17) and Seurat (4.3.0) (31178118). Briefly, filtered matrices containing cell IDs and feature names in each sample were used to build a Seurat object. We performed quality control by filtering out the cells with less than 200 detected genes, the genes detected in less than three cells and the cells exhibiting more than 10% of mitochondrial genes. Gene counts in each sample were normalized separately by default method 'LogNormalize' with a scale factor of 10,000 and log transformation. Two thousand highly variable features were identified with the 'vst' method. After merging cells from all samples, cell contaminants were removed based on the expression of cell-specific genes, and 12 clusters were identified in the remaining cells using the FindClusters function (15 Principal Components [PC] included and a resolution of 0.7 was selected) and the differentially expressed genes (DEGs) were calculated using the FindAllMarkers function (Seurat package). ScRNA_seq datasets containing steady-state $CD64^+$ lung cells (GSE194021) were integrated with the lung myeloid cells of this study using FindIntegrationAnchors function (Seurat) with anchor.features = 2000.

Single-cell regulatory network inference and clustering analysis

To predict the potential active transcription factors, lung myeloid cells analyzed by scRNA-seq were subjected to SCENIC analysis (*36*). The normalized counts, nFeature_RNA and nCount_RNA in the merged Seurat object were used for the initial SCENIC analysis. The genes expressed with a value of 3 in 0.1% of the cells and detected in 1% of the cells were kept, and coexpression network analysis was made with GENIE3 in the SCENIC package. To represent the SCENIC results, the results of the '3.4_regulonAUC' output were added to the metadata of Seurat object so that regulon AUC scores could be plotted as a heatmap.

Slingshot and tradeSeq pseudotime trajectory analyses

To evaluate trajectory-based differential expression analysis, $CD206^-$ IMs, $Ly6G^+$ Macs, $Ly6C^+$ Mos, iMos, $CD206^+$ IMs and dying Macs were subjected to Slingshot analysis (*32*). The trajectories along pseudotime were built using umap embedding from the Seurat object. To

compare the expression patterns of DEGs across pseudotime, the counts matrix, pseudotime and cell weights calculated above were then used as input in fitGAM function (tradeSeq package) (*85*). The association of average expression of each gene with pseudotime was tested using associationTest and the DEGs between IMs and Ly6G$^+$ Mac trajectories were calculated with the patternTest function. The value of the estimated smoother on a grid of pseudotimes was estimated for each DEG using predictSmooth. The 200 DEGs with the biggest FcMedian and waldStat > 200 were annotated as 'changed genes', meaning that their expression patterns were different in IMs and Ly6G$^+$ Mac trajectories, while the 200 genes whose average expression was associated with pseudotime in both lineages were selected based on their Fold change and labeled as 'unchanged genes'. Genes whose expression patterns appeared to be influenced by a small number of cells behaving as outliers were manually removed. Finally, the scaled estimated smoothers calculated by predictSmooth were used to build heat maps with the ComplexHeatmap package (*86*).

Gene Set Enrichment Analyses (GSEA)

In order to analyse enrichment of published signatures in the scRNA-seq data, the normalized counts were used as expression datasets in GSEA. GSEA was carried out using the GSEA software (version 4.1.0) (*87*). We used the hallmark gene sets from the Molecular Signatures Database (MSigDB) to test for enrichment. The analyses involved a gene set permutation method with 1,000 permutations to calculate the enrichment scores.

scRNA-seq of human BALF cells

Chromium Fixed RNA Profiling for multiplexed samples (10X Genomics) was used for scRNA-seq analysis of human BALF cells, allowing the storage of fixed cells and enabling analysis of multiple samples in one single GEM reaction. Fresh samples were directly fixed in a 4% formaldehyde solution after collection for storage at -80°C. For GEM creation, the Multiplex-compatible Chromium Next GEM Single Cell Fixed RNA Human Transcriptome Probe Kit including a Probe Barcode that permits sample multiplexing and subsequent demultiplexing was used. The Cell Ranger (v7.1.0) application (10x Genomics) was used to demultiplex the BCL files into FASTQ files (cellranger mkfastq), to perform alignment (to Cell Ranger human genome reference GRCh38-2020-A), filtering and unique molecular identifier counting and to produce gene-barcode matrices. Filtered matrix files were used for further scRNA-seq analyses. Samples from two different multiplexed batches were integrated with FindIntegrationAnchorsfunction (using canonical correlation analysis). A total of 19 clusters were identified, with 16 PCs were included and a resolution of 0.9.

Orthologous genes of the Ly6G$^+$ Mac signature in humans were manually identified using the gene database of NCBI. The signature was then used to calculate the score for each cell using AddModuleScore function (Seurat). The scores were stored in the seurat object and plotted using FeaturePlot function.

Single-cell regulatory network inference and clustering analysis was performed on scRNA-seq data from human BALF cells, as explained above.

Transmission electron microscopy

FACS-sorted myeloid cell populations or lung tissues from IAV-infected mice at day 10 post-IAV were fixed in 2.5% glutaraldehyde (diluted in Sorensen's buffer: 0.1 M Na2HPO4/NaH2PO4 buffer, pH 7.4) for 1h at 4 °C and postfixed for 30 min in 2% OsO4 (diluted in 0.1 M Sorensen's

Buffer). After dehydration in graded ethanol, samples were embedded in Epon resin. Ultrathin sections obtained with a Reichert Ultracut S ultramicrotome (Reichert Technologies) were contrasted with 2% uranyl acetate and 4% lead citrate.

For ultrastructural analyses, random fields of cells were examined under a Jeol TEM JEM-1400 Transmission Electron Microscope at 80 kV, and photographed using an 11-megapixel camera system (Quemesa, Olympus).

Extracellular flux analysis

Oxygen consumption rate (OCR) was measured using Seahorse XF Cell Mito Stress Test (Agilent) according to manufacturer's recommendations and as described previously (*70*, *71*). Briefly, Neu, IM-like cells and Ly6G$^+$ Macs were FACS-sorted at day 10 post-IAV and seeded (10.10^4, 7.10^4 and 8.10^4 cells/well, respectively) in XFp mini-plates (Agilent) pre-coated with CellTak. Cells were kept in unbuffered serum-free DMEM supplemented with pyruvate (1mM), glutamine (2mM), glucose (10mM), at pH 7.4, 37 °C and ambient CO_2 for 1h before the assay. Analysis was performed using the XFp analyser (Seahorse Bioscience) as per manufacturer's instructions. Cells were sequentially challenged with 1 µM oligomycin, 1 µM carbonyl cyanide p-(trifluoromethoxy) phenylhydrazone (FCCP), and rotenone/antimycin mix (0.5 µM each). All results were normalized according to the cell number evaluated by Hoechst (2 mg ml^{-1}) incorporation after cold methanol/acetone fixation.

Spatial transcriptomic analyses using Digital Spatial Profiling (DSP)

Five-µm-thick formalin-fixed, paraffin-embedded (FFPE) sections were prepared using the protocol from NanoString Technologies. Briefly, 2 tissue slides, each containing 1 mock and 2 IAV samples harvested 10 days post-IAV, were analyzed. Slides were first stained with antibodies against CD68, Ly6G (clone 1A8), and DNA was visualized with 500 nM Syto83. Mouse Whole Transcriptome Atlas probes targeting more than 19,000 targets were hybridized, and slides were loaded on the GeoMx DSP. Briefly, entire slides were imaged at x20 magnification, and Regions of Interest (ROIs) were chosen based on serial Hematoxylin & Eosin sections and on morphological markers to select lesional, perilesional and extralesional areas. ROIs were exposed to ultraviolet light, releasing the indexing oligos and collecting them in a 96-well plate for subsequent processing and sequencing, as described (*72*). Raw count, third quartile (Q3)–normalized count data of target genes from ROIs were provided by the vendor, which were used as input to downstream analyses. Pairwise differential expression analysis between perilesional, lesional, extralesional and control ROIs were performed using the GeoMx Digital Spatial Profiler Data Analysis Suite (DSPDA version 3.0.0.111). The R script SpatialDecon (*88*) was loaded into the DSPDA and run using the Mouse Adult Lung profile matrix. For cell signature scoring, the gene signatures were obtained from scRNA-seq data using the 20 most specific markers obtained using the FindAllMarkers function (Seurat package), genes were then ordered according to their average $\log_2$FC. Lung myeloid cell signature scores were obtained from our own scRNA-seq datasets, while the AT2, primed AT2, DATPs and AT1 signature score were calculated from previously published data (*38*) using the same procedure. The geneset from the activation pathway of IL4 (BIOCARTA_IL4_PATHWAY) was download from MSigDB and was used to generate the type 2 signature score shown in Fig. 7A. The signatures were then used to calculate the score for each ROI using the simpleScore function (singscore package) (*89*) on the ranked gene expression matrix.

Immunofluorescence

Immunofluorescence stainings of mouse lungs were performed as previously described (*39*). Briefly, lungs from WT or *Cx3cr1*$^{GFP+}$ mice were perfused with 5 ml PBS through the right ventricle then with 5 ml paraformaldehyde (PFA) 4% (Thermo Fisher) in PBS, and lungs were collected. Lungs were fixed for 4 h in 4% PAF at 4 °C, then cryoprotected overnight in 30% sucrose (VWR) in PBS at 4 °C, followed by embedding in optimal cutting temperature compound (OCT) (VWR) and stored at −80 °C.

For staining of lungs from *Cx3cr1*$^{GFP+}$ mice (Fig. 5A), 7-µm-thick sections were cut and left in a methanol 100% (Merck) bath at −20 °C for 20 minutes prior to be stained for 2h at room temperature with a rabbit anti-GFP antibody (ThermoFischer) and a rat anti-mouse Ly6G (BDBioscience). After washing samples with PBS, a secondary anti-rat AF594 (Invitrogen) was added in blocking buffer and incubated for 2 hours in the dark at room temperature.

For stainings of WT lungs (Fig. 5H and fig. S8), 7-µm-thick sections were cut and left in a methanol 100% (Merck) bath at −20 °C for 20 minutes prior to be stained overnight at 4°C in blocking buffer (PBS with 0.3% Triton X-100 [Merck], 2% donkey serum [Merck]) with the following antibodies: rabbit anti-mouse pSPC (Abcam); rat anti-mouse Ly6G (BDBioscience). After washing samples with PBS, secondary antibodies (anti-rabbit AF532; anti-rat AF594 [Invitrogen]) were added in blocking buffer and incubated for 2 hours in the dark at room temperature. Samples were washed with PBS and incubated with directly-coupled antibodies (eFluor570-Ki67 [Invitrogen]; anti-mouse AF700-MHC-II [ThermoFischer]; anti-mouse Superbright432-Pdpn [ThermoFischer]) in blocking buffer for 6 hours at 4 °C.

Finally, all samples were washed one last time with PBS and were mounted with 10 µl ProLong Antifade reagent (Invitrogen) containing 0.1% Sytox blue nucleic acid stain (Invitrogen) on glass slides and stored at room temperature in the dark overnight.

Images shown in Fig. 3K and 5A were acquired on an LSM 980 inverted confocal microscope using Plan-Apochromat 20x/0.8 or LD C-Apochromat 40x/1.1 W objectives. Fluorophores were excited simultaneously at 405/561 nm (Fig. 3K) or 405/488/561 nm (Fig. 5A) with detection wavelength at 300-735/499-594/573-627 with GaAsP-PMT in Zeiss FastAiryScanSheppardSum SR-4y:3.7 mode and bidirectional acquisition. Analysis was performed with Zeiss Blue software.

Images shown in Fig. 5H and fig. S8 were acquired on a Leica Stellaris 8 Inverted Confocal microsocope with a White Light Laser (WLL) using 20x APO CS2 or 40x 1.30 NA Oil objectives and 512x512, 400 Hz, unidirectional acquisition. The acquisition was finalized by 3 sequential settings, all with WLL at 85% and a pinhole at 1 AU (65,3). We used, for the first sequence, the combination of diode laser 405 (dsetector HyD S 415-443 nm) and WLL with a laser line of 556 nm (Detector HyD S 570-620nm); for the second sequence, the WWL with a laser line of 528 nm (Detector HyD S 541-564 nm) and 445 nm (Detector HyD S 450-515nm); for the third sequence, the WLL with a laser line of 499nm (Detector HyD S 504-539 nm) and 696nm (Detector HyD X 706-752 nm). The images were then analyzed with LAS X software version 4.7.0.28176.

Histology

Seven-µm-thick sections from frozen lung tissues obtained from mock- and IAV-infected mice at day 20 post-IAV were mounted onto glass slides and stained with Hematoxylin & Eosin (H&E) or periodic-acid Schiff (PAS). The slides were scanned with an Axioscan 7 scanner (Zeiss,

Germany). Whole slide images were analysed with an open-source automated software analysis program for digital pathology (QuPath version 0.4.3). Briefly, lesional areas were determined manually and automated tissue detection was performed in the lesional area to correct for alveolar blank spaces. Thereafter, built-in algorithms for pixel classification of QuPath and machine learning were used on PAS sections and a threshold was determined to quantify % of mucus^{+} cells within the lesional area.

***Ex vivo* experiments**

For *ex vivo* stimulation and co-culture experiments, single cell suspensions isolated from IAV-infected lungs of WT or *Ly6g^{tdTom}* mice at day 10 post-IAV were enriched in CD11b^{+} cells by a magnetic-activated cell sorting (MACS) using CD11b MicroBeads (Myltenyi). Cells were then stained and FACS-sorted using the gating strategy shown in Fig. 1A. After sorting, cells were counted, spinned down, and either directly added to the co-culture with MLE-12 cells, or seeded in 96 wells at a concentration 5.10^{4} cells/well in complete RPMI (ThermoFischer), containing 1mM sodium pyruvate, 1% vol/vol MEM non-essential amino acids, 50 U ml^{-1} Penicillin-Streptomycin and 10% vol/vol FBS. For stimulation experiments, recombinant mouse GM-CSF (20 ng ml^{-1}, Peprotech), mouse M-CSF (20 ng ml^{-1}, Peprotech), mouse IL-4 (20 ng ml^{-1}, Peprotech) or mouse IL-13 (20 ng ml^{-1}, Peprotech) were added. When required, Cre-ERT2 activation was achieved by adding 0.02 mg ml^{-1} of 4-hydroxytamoxifen (Sigma-Aldrich). After 18 hours of culture, cell supernatants were collected (conditioned medium, CM) and cells were harvested for flow cytometry phenotyping. For the visualisation of tdTomato induction in tamoxifen-treated iMos from *Ly6g^{tdTom}* mice by confocal microscopy, iMos were seeded and cultured in 8-chambers slides (Nunc Lab-Tek II Chamber Slide system, Sigma) precoated with poly-D-lysin hydrobromide (Sigma). Cells were incubated in complete RPMI containing 0.02 mg ml^{-1} 4-hydroxytamoxifen and 20ng ml^{-1} GM-CSF or vehicle. Supernatants were removed and chambers were rinsed with PBS. Slides were then fixed with paraformaldehyde 10% for 10 minutes, rinsed twice with PBS and mounted with ProLong Antifade reagent with DAPI (ThermoFisher). Images were acquired as above.

Proteome profiler (R&D) was performed on CM from Ly6G^{+}Macs cultured for 18h in complete RPMI with or without 20ng ml^{-1} IL-4 and 20ng ml^{-1} IL-13 treatment. The assay was performed following the manufacturer's instructions and was analysed using the Protein Array Analyzer plug-in for ImageJ.

Murine lung epithelial (MLE)-12 cells (ATCC, CVCL_3751) were cultured in DMEM/F12 (ThermoFisher) complemented with 1% Insulin-Transferrin-Selenium (ITS-G) (ThermoFisher), L-glutamine 2mM (ThermoFisher), FBS 2% and HEPES 10mM (ThermoFisher). Cells were incubated at 37 °C in a humidified atmosphere containing 5% CO2. Cells were passaged at 80–90% confluence using 0.05% Trypsin-EDTA (ThermoFisher). Experiments were performed with passage numbers ranging from 4 to 6. Scratch Wound Assay were performed using IncucyteS3 (Sartorius). MLE-12 cells were seeded in 96-well (Sartorius) at density of 4.10^{4} cells/well and incubated 24 hours in DMEM/F12 medium. An open wound area was created in the cell monolayer using the IncuCyte ® Wound Maker tool, washed with PBS and subsequently co-cultured with 5.10^{4} Neu, IM-like cells, iMos or Ly6G^{+} Macs, or incubated with CM from unpulsed or IL-4/IL-13-pulsed Ly6G^{+} Macs. Complete DMEM medium containing 20 ng ml^{-1} IL-4 and 20 ng ml^{-1} IL-13 was used as control. Cells were imaged after wounding every 3 hours at 10 x magnification using the Sartorius Incucyte S3 Inverted brighfield microscope with motorized XYZ in a chamber

for 37°C temperature, 5% CO2 and 90-95% humidity. Images were acquired by Basler Ace 1920-155um, acA1920-155umEBS camera with 10 x 0.3 NA, Dry, 16mm WD objective. For each time point, relative wound closure was calculated using the Scratch Wound analyses pipeline of the IncuCyte 2023A Rev1 software.

Adoptive transfer of Ly6G$^+$ Macs *in vivo*

Ly6G$^+$ Macs were isolated from the lungs of CD45.2 WT mice at day 10 poist-IAV. Lung single cell suspensions were first enriched in CD11b$^+$ cells by MACS using CD11b MicroBeads (Miltenyi Biotec) and were FACS-sorted using a Sony MA900. Four hundred thousands (4 x 10^5) Ly6G$^+$ Macs were resuspended in 50 µl sterile PBS and were instilled i.t. in lightly isoflurane-anesthetized *Maf/Mafb*^{MyeloKO} mice at days 8, 11, 13, 15 post-IAV. Control *Maf/Mafb*^{MyeloKO} mice and WT mice received 50 µl PBS as vehicle.

Quantification of alanine aminotransferase (ALT)

Blood was collected in a a 100 mM EDTA (Merck Millipore)-containing tube and centrifuged at 4000 rpm for 4 minutes. Plasma was collected and levels of ALT were determined usinf a mouse ALT ELISA Kit (Abcam) following manufacturer's instructions.

Human BALFs

The use of human BALF cells was approved in 2022 by the Ethics Reviewing Board of the University Hospital of Liege, Belgium (ref. 2022/159). The characteristics of the patients are summarized in Table S1. Human BALFs were fixed directly after collection for storage and scRNA-seq analyses.

Statistical analysis

Graphs were prepared with GraphPad Prism 9 (GraphPad software) or R Bioconductor (3.5.1) (*73*). Data distribution was assumed to be normal when parametric tests were performed. Data from independent experiments were pooled for analysis in each data panel, unless otherwise indicated. No data were excluded from the analyses. Statistical analyses were performed with Prism 9 (GraphPad software), and with R Bioconductor (3.5.1) (*73*) and Seurat (*76*) for scRNA-seq data, respectively. The statistical analyses performed for each experiment are indicated in the respective figure legends. We considered a P value lower than 0.05 to be significant (*, $P < 0.05$; **, $P < 0.01$; ***, $P < 0.001$; ****, $P < 0.0001$; ns, not significant).

Additional sections and details about Materials and Methods can be found in the Supplemental Materials.

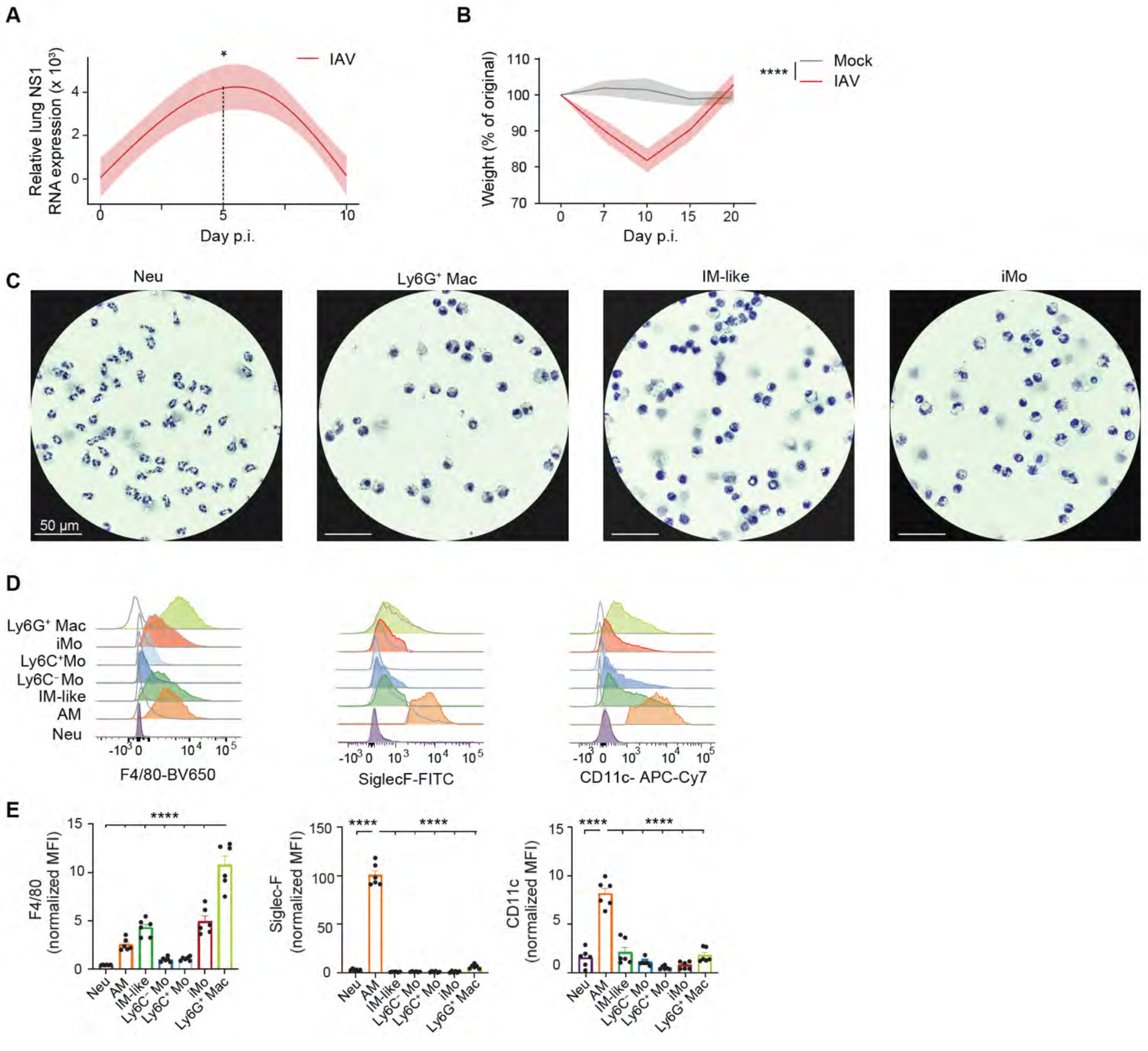

Fig. S1. Morphology and phenotype of lung myeloid cells at day 10 post-IAV. (**A**) Time course of relative lung NS1 RNA expression, assessed by RT-qPCR at days 0, 5 and 10 post-IAV in WT mice. (**B**) Time course of weight, expressed as the % of the original weight at day 0 and assessed at days 0, 7, 10, 15 and 20 post-IAV in WT mice. (**C**) Photographs of Neu, Ly6G$^+$ Macs, IM-like cells and iMo sorted by FACS from IAV-infected WT mice at day 10 p.i.. Pictures are representative of 1 of 3 independent sorting experiments, each giving similar results. (**D**) Representative histograms of F4/80, SiglecF and CD11c expression in the indicated myeloid cell populations, quantified by flow cytometry at day 10 post-IAV in WT mice. (**E**) Quantification of expression of the indicated markers, as in (D). (A,B) Data show mean (centerline) ± SEM (colored area) and are pooled from 2 independent experiments (*n*=6-7 mice per time point). (E) Data show mean + SEM and are pooled from 2 independent experiments (*n*= 6 mice). *P* values compare day 5 vs. day 0 in (A) and were calculated using (A,D) a one-way ANOVA with Dunnett's post hoc tests or (B) a two-way ANOVA. *, P<0.05; ****, P<0.0001. p.i., post-infection. (C) Scale bar: 50 μm.

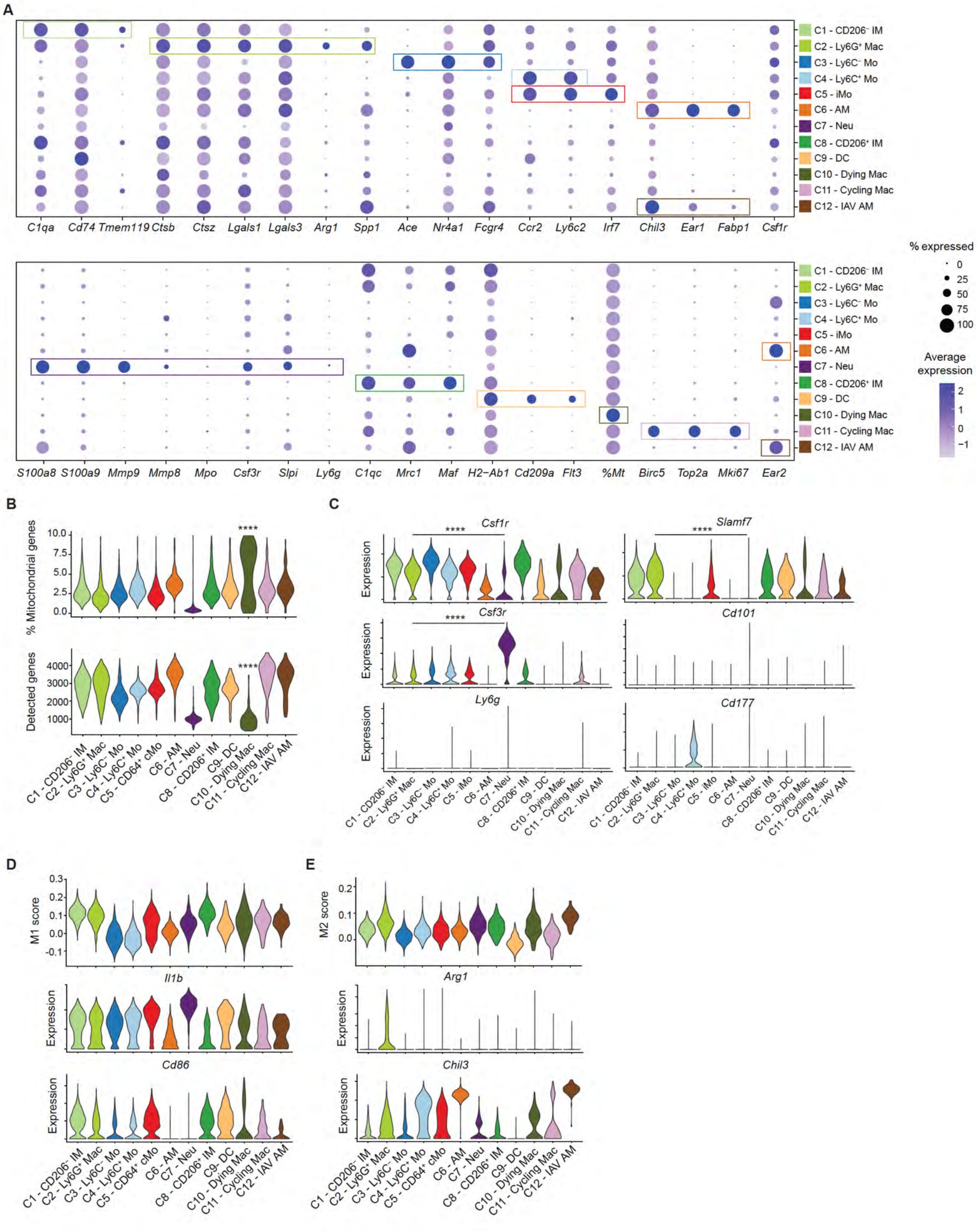

A
B
C
D
E
C1 - CD206⁻ IM
C2 - Ly6G⁺ Mac
C3 - Ly6C⁻ Mo
C4 - Ly6C⁺ Mo
C5 - iMo
C6 - AM
C7 - Neu
C8 - CD206⁺ IM
C9 - DC
C10 - Dying Mac
C11 - Cycling Mac
C12 - IAV AM
C1qa Cd74 Tmem119 Ctsb Ctsz Lgals1 Lgals3 Arg1 Spp1 Ace Nr4a1 Fcgr4 Ccr2 Ly6c2 Irf7 Chil3 Ear1 Fabp1 Csf1r
S100a8 S100a9 Mmp9 Mmp8 Mpo Csf3r Slpi Ly6g C1qc Mrc1 Maf H2−Ab1 Cd209a Flt3 %Mt Birc5 Top2a Mki67 Ear2
% expressed
Average expression
% Mitochondrial genes
Detected genes
C5 - CD64⁺ cMo
Expression
Csf1r
Slamf7
Csf3r
Cd101
Ly6g
Cd177
M1 score
Il1b
Cd86
M2 score
Arg1
Chil3
****

Fig. S2. Gene expression in and features of myeloid cell clusters identified by scRNA-seq at day 10 post-IAV. (**A**) Dot plots showing average expression of the indicated genes and % of cells expressing the genes within each cluster, related to Fig. 2A. (**B**) Percentage of mitochondrial genes (top) and number of detected genes (bottom) within each cluster, as depicted by violin plots (width: abundance of cells). (**C**) Expression of the indicated genes within each cluster, as depicted by violin plots (height: gene expression; width: abundance of cells). (**D**) M1 signature score (top) and expression of M1-related genes within each cluster, as depicted by violin plots (height: M1 score or gene expression; width: abundance of cells). (**E**) M2 signature score (top) and expression of M2-related genes within each cluster, as depicted by violin plots (height: M2 score or gene expression; width: abundance of cells). *P* values compare (B) C10 – dyings Macs *vs.* all other clusters or (C) C2 – Ly6G^{+} Macs *vs.* C7 – Neutrophils and were calculated using a Wilcoxon rank sum test. ****, *P*<0.0001.

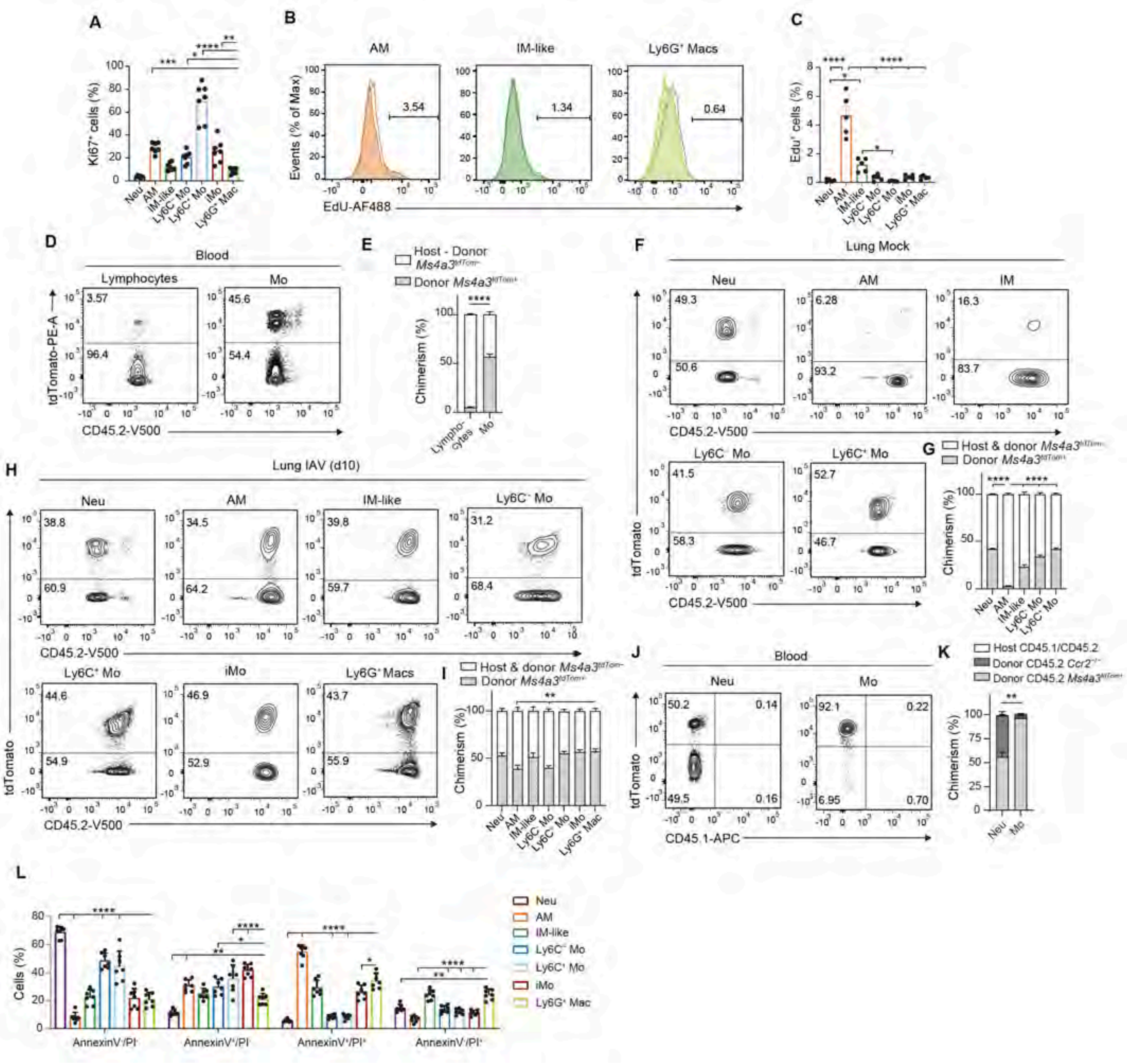
A
Ki67+ cells (%)
Neu
AM
IM-like
Ly6C- Mo
Ly6C+ Mo
iMo
Ly6G+ Mac
B
AM
IM-like
Ly6G+ Macs
Events (% of Max)
3.54
1.34
0.64
EdU-AF488
C
Edu+ cells (%)
D
Blood
Lymphocytes
Mo
tdTomato-PE-A
3.57
96.4
45.6
54.4
CD45.2-V500
E
Host - Donor Ms4a3tdTom-
Donor Ms4a3tdTom+
Chimerism (%)
Lymphocytes
Mo
F
Lung Mock
Neu
AM
IM
49.3
50.6
6.28
93.2
16.3
83.7
CD45.2-V500
Ly6C- Mo
Ly6C+ Mo
41.5
58.3
52.7
46.7
tdTomato
G
Host & donor Ms4a3tdTom-
Donor Ms4a3tdTom+
Chimerism (%)
H
Lung IAV (d10)
Neu
AM
IM-like
Ly6C- Mo
38.8
60.9
34.5
64.2
39.8
59.7
31.2
68.4
CD45.2-V500
Ly6C+ Mo
iMo
Ly6G+Macs
44.6
54.9
46.9
52.9
43.7
55.9
tdTomato
I
Host & donor Ms4a3tdTom-
Donor Ms4a3tdTom+
Chimerism (%)
J
Blood
Neu
Mo
50.2
0.14
49.5
0.16
92.1
0.22
6.95
0.70
tdTomato
CD45.1-APC
K
Host CD45.1/CD45.2
Donor CD45.2 Ccr2-/-
Donor CD45.2 Ms4a3tdTom+
Chimerism (%)
L
Cells (%)
AnnexinV-/PI-
AnnexinV+/PI-
AnnexinV+/PI+
AnnexinV-/PI+
Neu
AM
IM-like
Ly6C- Mo
Ly6C+ Mo
iMo
Ly6G+ Mac

Fig. S3. Ly6G$^+$ Macs do not proliferate, are short-lived and arise from recruited BM-derived monocytes post-IAV. (**A**) Bar graphs showing the % of Ki67$^+$ cells in the indicated lung myeloid cell populations, as quantified by flow cytometry in lungs of WT mice at day 10 post-IAV. (**B**) Representative histograms of EdU levels in AMs, IM-like cells and Ly6G$^+$ Macs from EdU-pulsed WT mice at day 10 post-IAV. Unpulsed mice were used as controls (grey line). (**C**) Bar graphs showing the % of EdU$^+$ cells in the indicated lung myeloid cell populations, as in (B). (**D**) Representative tdTomato and CD45.2 contour plots and (**E**) bar graph showing % of host and donor *Ms4a3*$^{tdTom-}$ chimerism and donor *Ms4a3*$^{tdTom+}$ chimerism of blood lymphocytes and monocytes (Mo) from lethally-irradiated thorax-protected WT mice reconstituted with *Ms4a3*$^{tdTom+}$ BM donor cells, evaluated by flow cytometry 4 weeks after reconstitution. (**F**) Representative tdTomato and CD45.2 contour plots and (**G**) bar graph showing % of host and donor *Ms4a3*$^{tdTom-}$ chimerism and donor *Ms4a3*$^{tdTom+}$ chimerism of the indicated lung myeloid cell populations, as in (D-E), evaluated by flow cytometry 4 weeks after reconstitution and 10 days after mock infection. (**H**) Representative tdTomato and CD45.2 contour plots and (**I**) bar graphs showing % of host + donor *Ms4a3*$^{tdTom-}$ chimerism and donor *Ms4a3*$^{tdTom+}$ chimerism of the indicated lung myeloid cell populations, as in (F-G), evaluated by flow cytometry 4 weeks after reconstitution and 10 days after IAV infection. (**J**) Representative tdTomato and CD45.1 contour plots and (**K**) bar graph showing % of host CD45.1/CD45.2, CD45.2 donor *Ccr2*$^{-/-}$ and *Ms4a3*$^{tdTom+}$ chimerism of blood neutrophils (Neu) and Mo from lethally-irradiated CD45.1/CD45.2 mice reconstituted with a 1:1 mix of CD45.2 *Ccr2*$^{-/-}$ and *Ms4a3*$^{tdTom+}$ BM cells, evaluated by flow cytometry 4 weeks after reconstitution. (**L**) Bar graph showing the frequency of Annexin V and PI negative and/or positive fractions within the indicated lung myeloid cell populations, quantified by flow cytometry at day 10 post-IAV in WT mice. (A,C,E,G,I,K,L) Data show mean + SEM and are pooled from 2 independent experiments (n=4-10 mice). *P* values compare donor *Ms4a3*$^{tdTom+}$ chimerism in (D,F,H,J) and were calculated using (A,C) a one-way ANOVA with Dunnett's post hoc tests, (E,K) a two-way ANOVA with Sidak's post hoc tests or (G,I,L) a two-way ANOVA with Tukey's post hoc tests. *, $P<0.05$; **, $P<0.01$; ****, $P<0.0001$.

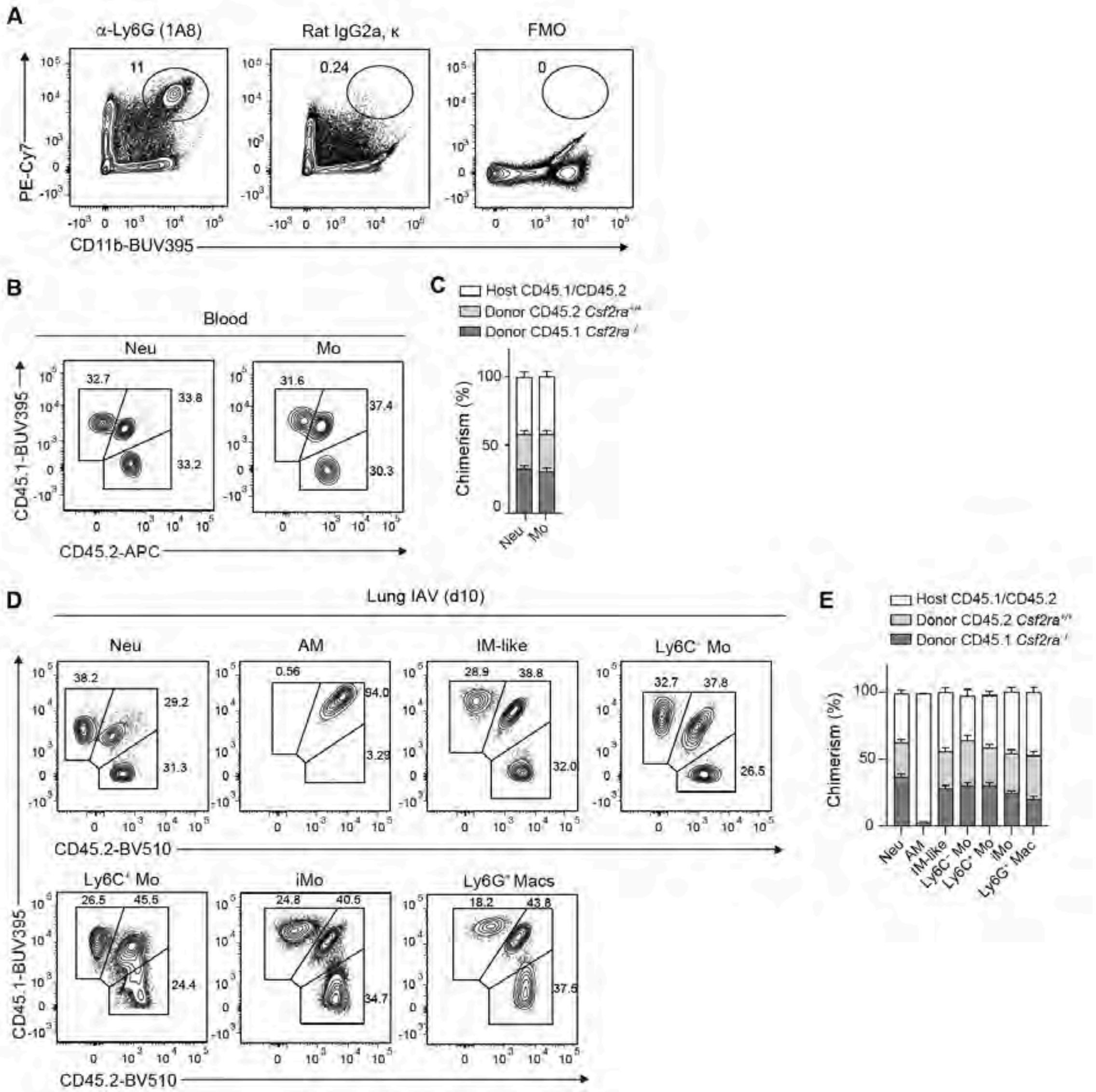

Fig. S4. Specificity of the anti-Ly6G staining and analysis of IAV-infected *Csf2ra*$^{-/-}$: *Csf2ra*$^{+/+}$ mixed BM chimeras. (**A**) Representative contour plots of PE-Cy7 and CD11b expression within lung live CD45^{+} cells in IAV-infected WT mice at day 10 post-IAV. Plots are representative of 1 of 6 mice analyzed, each giving similar results. (**B**) Representative CD45.1 and CD45.2 contour plots and (**C**) bar graph showing % of host CD45.1/CD45.2, CD45.2 donor *Csf2ra*$^{-/-}$ and CD45.1 *Csf2ra*$^{+/+}$ chimerism of blood neutrophils (Neu) and monocytes (Mo) from thorax-protected, lethally-irradiated CD45.1/CD45.2 mice reconstituted with a 1:1 mix of CD45.2 *Csf2ra*$^{-/-}$ and CD45.1 *Csf2ra*$^{+/+}$ BM cells, evaluated by flow cytometry 4 weeks after reconstitution. (**D**) Representative CD45.1 and CD45.2 contour plots and (**E**) bar graph showing % of host CD45.1/CD45.2, donor CD45.1 *Csf2ra*$^{-/-}$ and donor CD45.2 *Csf2ra*$^{+/+}$ chimerism of the indicated lung myeloid cell populations, as in (B-C), infected with IAV 4 weeks later and evaluated at day 10 post-IAV. (C,E) Data show mean + SEM and are pooled from 2 independent experiments (*n*=10 mice).

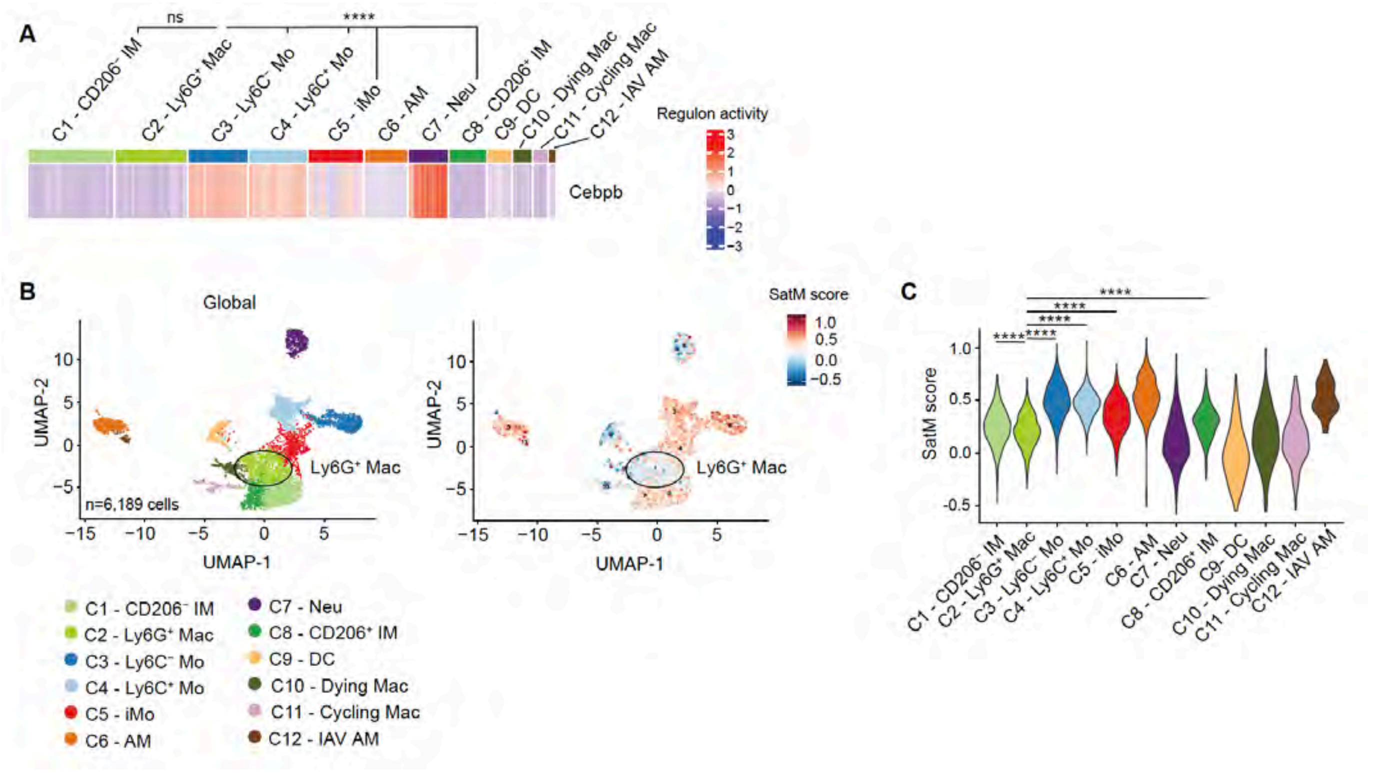

Fig. S5. Transcriptomic comparison of Ly6G^{+} Macs and SatM monocytes. (**A**) Heatmap depicting predicted activities of Cebpb across lung myeloid cells post-IAV, evaluated by SCENIC analysis of the scRNA-seq data shown in Fig. 2A. (**B**) UMAP plots of scRNA-seq data depicting (left) the transcriptional identity of sorted lung live CD45^{+}F4/80^{+} and/or CD11b^{+} cells from mock- or IAV-infected WT mice 10 days post-infection (5 mice per time points), merged with a published dataset of steady-state lung monocytes and IMs (*69*), and (right) a SatM monocyte signature score (*35*). (C) SatM monocyte signature score within each scRNA-seq cluster, as depicted by violin plots (height: score; width: abundance of cells). (A,C) *P* values were calculated using a Wilcoxon rank sum test. ****, *P*<0.0001. ns, not significant.

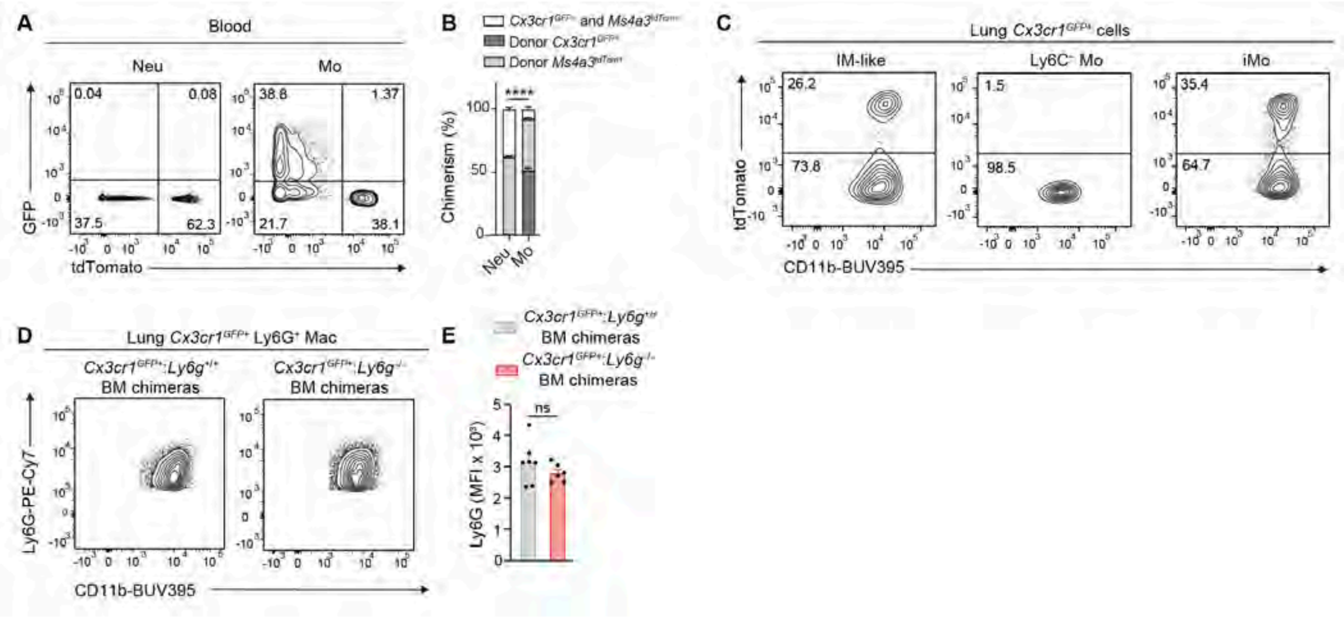

Fig. S6. Efferocytic abilities of lung myeloid cells post-IAV. (**A**) Representative GFP and tdTomato contour plots and (**B**) bar graph showing chimerism of *Cx3cr1*$^{GFP-}$*Ms4a3*$^{tdTom-}$, donor *Cx3cr1*$^{GFP+}$ and donor *Ms4a3*$^{tdTom+}$ cells in blood neutrophils (Neu) and monocytes (Mo) from lethally-irradiated CD45.2 WT mice reconstituted with a 1:1 mix of CD45.2 *Cx3cr1*$^{GFP+}$ and *Ms4a3*$^{tdTom+}$ BM cells, evaluated by flow cytometry 4 weeks after reconstitution. (**C**) Representative tdTomato and CD11b contour plots of the indicated lung *Cx3cr1*$^{GFP+}$ donor cell populations from lethally-irradiated CD45.2 WT mice reconstituted with a 1:1 mix of CD45.2 *Cx3cr1*$^{GFP+}$ and *Ms4a3*$^{tdTom+}$ BM cells, infected with IAV 4 weeks later and evaluated at day 10 post-IAV. (**D**) Representative Ly6G and CD11b contour plots of lung *Cx3cr1*$^{GFP+}$ Ly6G^{+} Macs from lethally-irradiated CD45.1/CD45.2 mice reconstituted with a 1:1 mix of CD45.2 *Cx3cr1*$^{GFP+}$ and *Ly6g*$^{+/+}$ (left) or *Ly6g*$^{-/-}$ (right) BM cells, infected with IAV 4 weeks later and evaluated at day 10 post-IAV. (**E**) Bar graph showing Ly6G expression levels in lung *Cx3cr1*$^{GFP+}$ Ly6G^{+} Macs, as in (D). (B,E) Data show mean + SEM and are pooled from 2 independent experiments (B: n=14 mice; E: n=6 mice). (B) P values compare donor *Cx3cr1*^{GFP} chimerism and were calculated using a two-way ANOVA with Sidak's post hoc tests. (E) P values were calculated using a two-tailed Student's t test. ns, not significant.

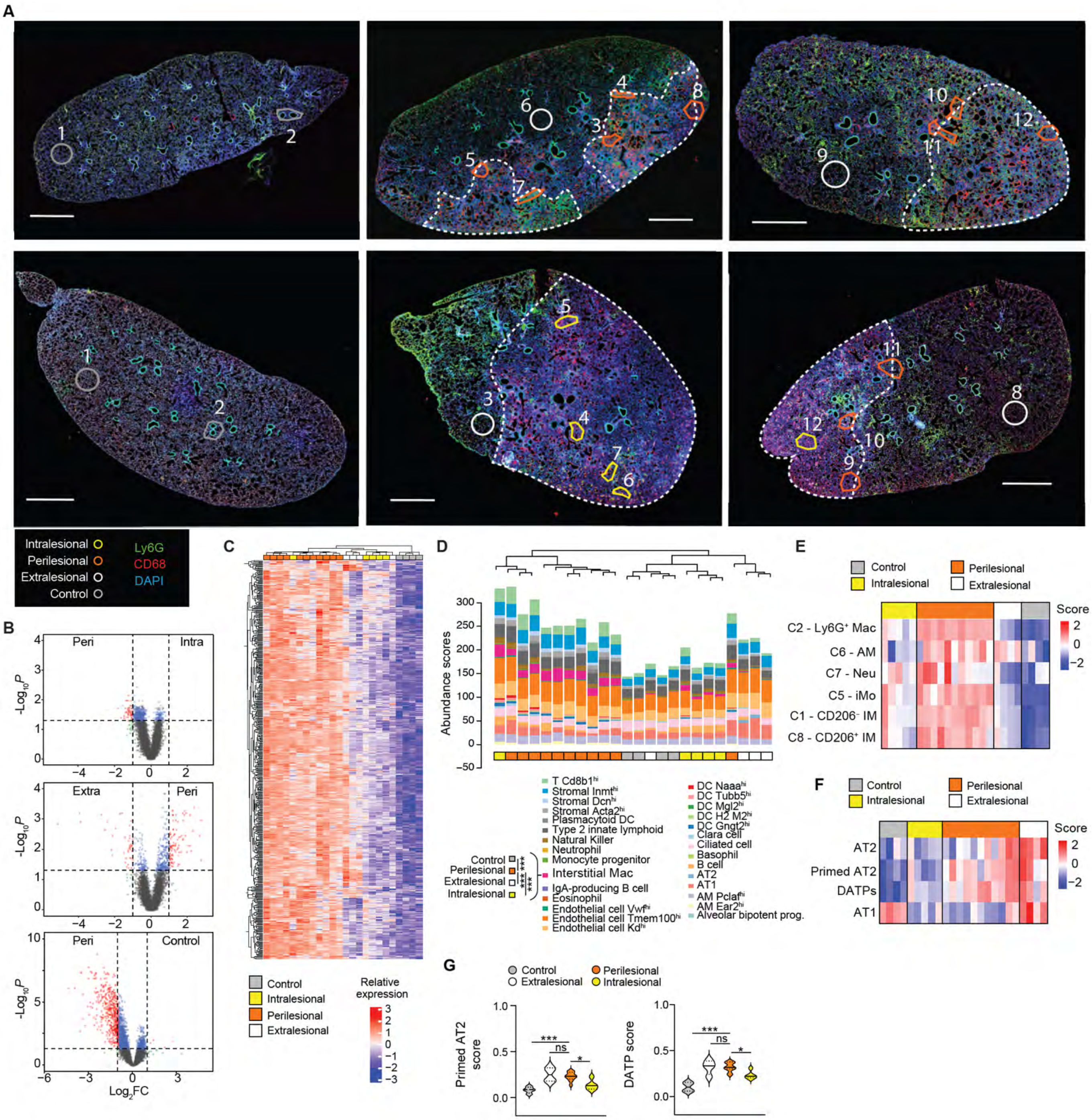
A
Intralesional
Perilesional
Extralesional
Control
Ly6G
CD68
DAPI
B
Peri
Intra
Extra
Peri
Peri
Control
-Log10P
Log2FC
C
Control
Intralesional
Perilesional
Extralesional
Relative expression
D
Abundance scores
T Cd8b1hi
Stromal Inmthi
Stromal Dcnhi
Stromal Acta2hi
Plasmacytoid DC
Type 2 innate lymphoid
Natural Killer
Neutrophil
Monocyte progenitor
Interstitial Mac
IgA-producing B cell
Eosinophil
Endothelial cell Vwfhi
Endothelial cell Tmem100hi
Endothelial cell Kdhi
DC Naaahi
DC Tubb5hi
DC Mgl2hi
DC H2 M2hi
DC Gngt2hi
Clara cell
Ciliated cell
Basophil
B cell
AT2
AT1
AM Pclafhi
AM Ear2hi
Alveolar bipotent prog.
E
Control
Intralesional
Perilesional
Extralesional
Score
C2 - Ly6G+ Mac
C6 - AM
C7 - Neu
C5 - iMo
C1 - CD206- IM
C8 - CD206+ IM
F
Control
Intralesional
Perilesional
Extralesional
Score
AT2
Primed AT2
DATPs
AT1
G
Control
Extralesional
Perilesional
Intralesional
Primed AT2 score
DATP score
ns

Fig. S7. Identification of Ly6G$^+$ Macs-AT2 clusters by confocal microscopy in lung perilesional areas post-IAV. (**A**) Selection of regions of interest (ROIs) on lung sections from mock- or IAV-infected WT mice at day 10 post-IAV, stained with anti-Ly6G and anti-CD68 antibodies. (**B**) Volcano Plot depicting the differentially expressed genes (DEGs) between perilesional (Peri) and intralesional (Intra) areas (top), extralesional (Extra) and Peri areas (middle), and Peri and control areas (bottom). (**C**) Heatmap depicting the significantly upregulated genes in Peri areas as compared to Intra areas. (**D**) Cell deconvolution of the ROIs and abundance score of cell populations in individual ROIs using SpatialDecon algorithm. (**E**) Heatmap showing the signature score of the indicated myeloid cell populations within individual ROIs, inferred from the scRNA-seq data presented in Fig. 2A. (**F**) Heatmap showing the signature score of transitional epithelial cell states during AT2-mediated regeneration after bleomycin-induced lung injury, inferred from previously published scRNA-seq data (***38***). (**G**) Primed AT2 (left) and DATPs (right) signature scores within control, extralesional, perilesional and intralesional ROIs, as in (F), as depicted by violin plots (height: scores; width: abundance of cells). *P* values were calculated using (D) a two-way ANOVA with Tukey's post-hoc tests or (G) a one-way ANOVA with Tukey's post-hoc tests. *, $P<0.05$; ***, $P<0.001$. ns, not significant.

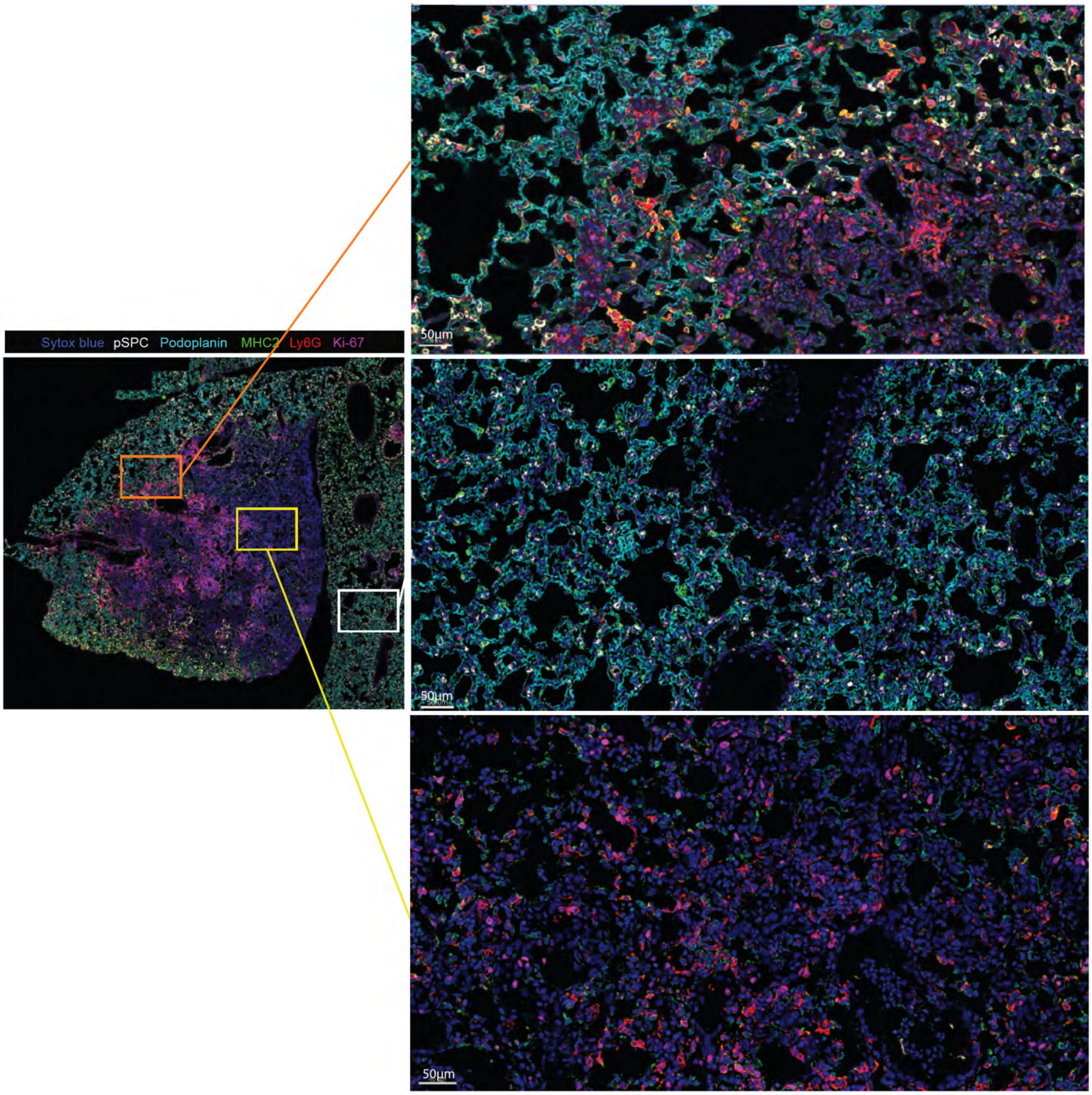

Sytox blue pSPC Podoplanin MHC2 Ly6G Ki-67
50µm
50µm
50µm

Fig. S8. Ly6G$^+$ Macs cluster with AT2 cells in perilesional areas. Representative high-resolution confocal laser scanning microscopy picture of a lung section from an IAV-infected WT mouse at day 10 post-IAV. Zooms of perilesional (orange) and intralesional (yellow) areas are shown. Pictures are representative of 1 of 6 mice analyzed, each of them giving similar results. Scale bar: 50 µm.

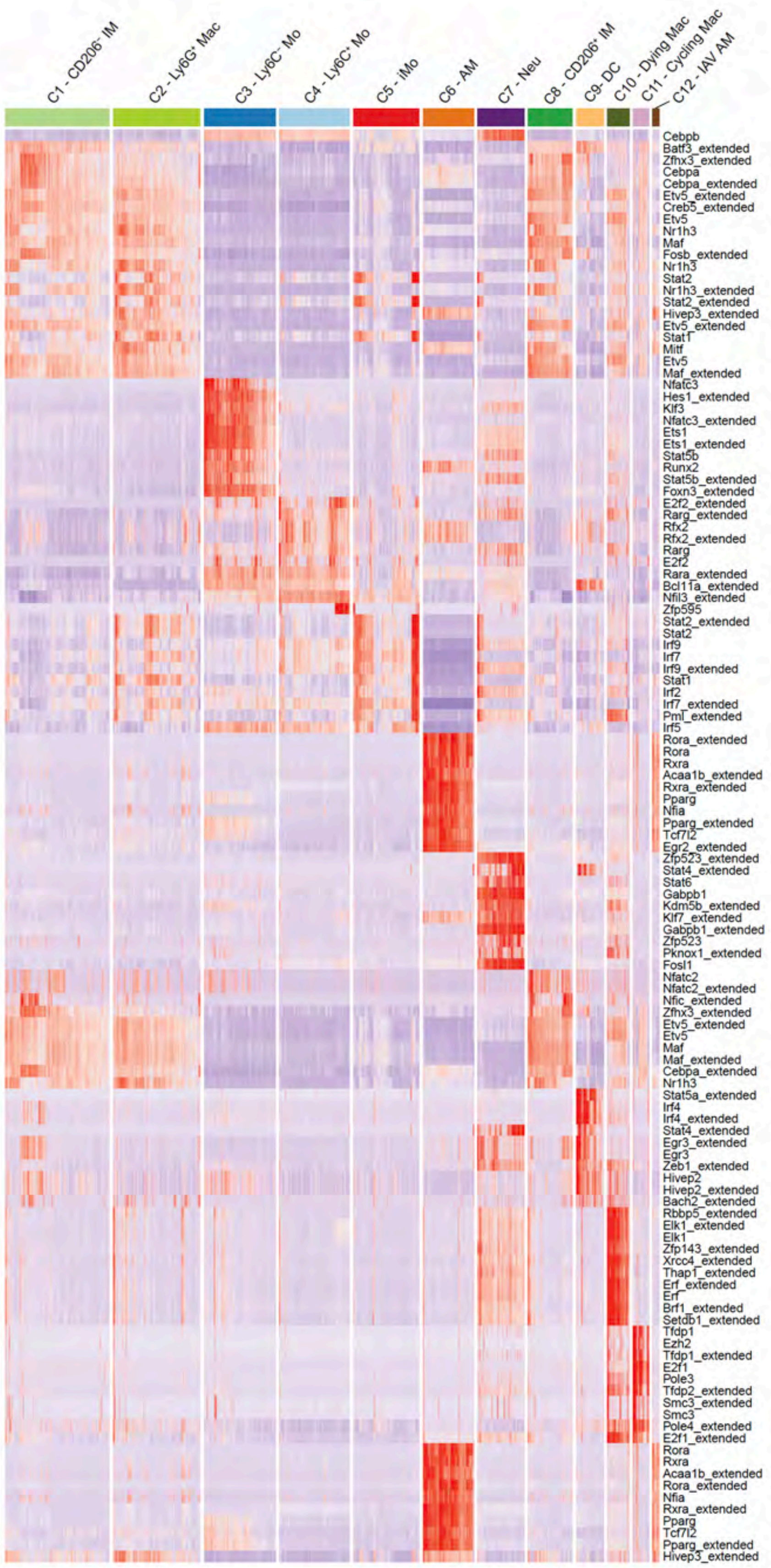

C1 - CD206⁻ IM
C2 - Ly6G⁺ Mac
C3 - Ly6C⁻ Mo
C4 - Ly6C⁺ Mo
C5 - iMo
C6 - AM
C7 - Neu
C8 - CD206⁺ IM
C9- DC
C10 - Dying Mac
C11 - Cycling Mac
C12 - IAV AM
Cebpb
Batf3_extended
Zfhx3_extended
Cebpa
Cebpa_extended
Etv5_extended
Creb5_extended
Etv5
Nr1h3
Maf
Fosb_extended
Nr1h3
Stat2
Nr1h3_extended
Stat2_extended
Hivep3_extended
Etv5_extended
Stat1
Mitf
Etv5
Maf_extended
Nfatc3
Hes1_extended
Klf3
Nfatc3_extended
Ets1
Ets1_extended
Stat5b
Runx2
Stat5b_extended
Foxn3_extended
E2f2_extended
Rarg_extended
Rfx2
Rfx2_extended
Rarg
E2f2
Rara_extended
Bcl11a_extended
Nfil3_extended
Zfp595
Stat2_extended
Stat2
Irf9
Irf7
Irf9_extended
Stat1
Irf2
Irf7_extended
Pml_extended
Irf5
Rora_extended
Rora
Rxra
Acaa1b_extended
Rxra_extended
Pparg
Nfia
Pparg_extended
Tcf7l2
Egr2_extended
Zfp523_extended
Stat4_extended
Stat6
Gabpb1
Kdm5b_extended
Klf7_extended
Gabpb1_extended
Zfp523
Pknox1_extended
Fosl1
Nfatc2
Nfatc2_extended
Nfic_extended
Zfhx3_extended
Etv5_extended
Etv5
Maf
Maf_extended
Cebpa_extended
Nr1h3
Stat5a_extended
Irf4
Irf4_extended
Stat4_extended
Egr3_extended
Egr3
Zeb1_extended
Hivep2
Hivep2_extended
Bach2_extended
Rbbp5_extended
Elk1_extended
Elk1
Zfp143_extended
Xrcc4_extended
Thap1_extended
Erf_extended
Erf
Brf1_extended
Setdb1_extended
Tfdp1
Ezh2
Tfdp1_extended
E2f1
Pole3
Tfdp2_extended
Smc3_extended
Smc3
Pole4_extended
E2f1_extended
Rora
Rxra
Acaa1b_extended
Rora_extended
Nfia
Rxra_extended
Pparg
Tcf7l2
Pparg_extended
Hivep3_extended

Fig. S9. SCENIC analysis of lung myeloid cells at day 10 post-IAV. Heat map depicting predicted transcription factor (TF) activities across lung myeloid cells at day 10 post-IAV, as assessed by SCENIC analysis of the scRNA-seq data shown in Fig. 2A.

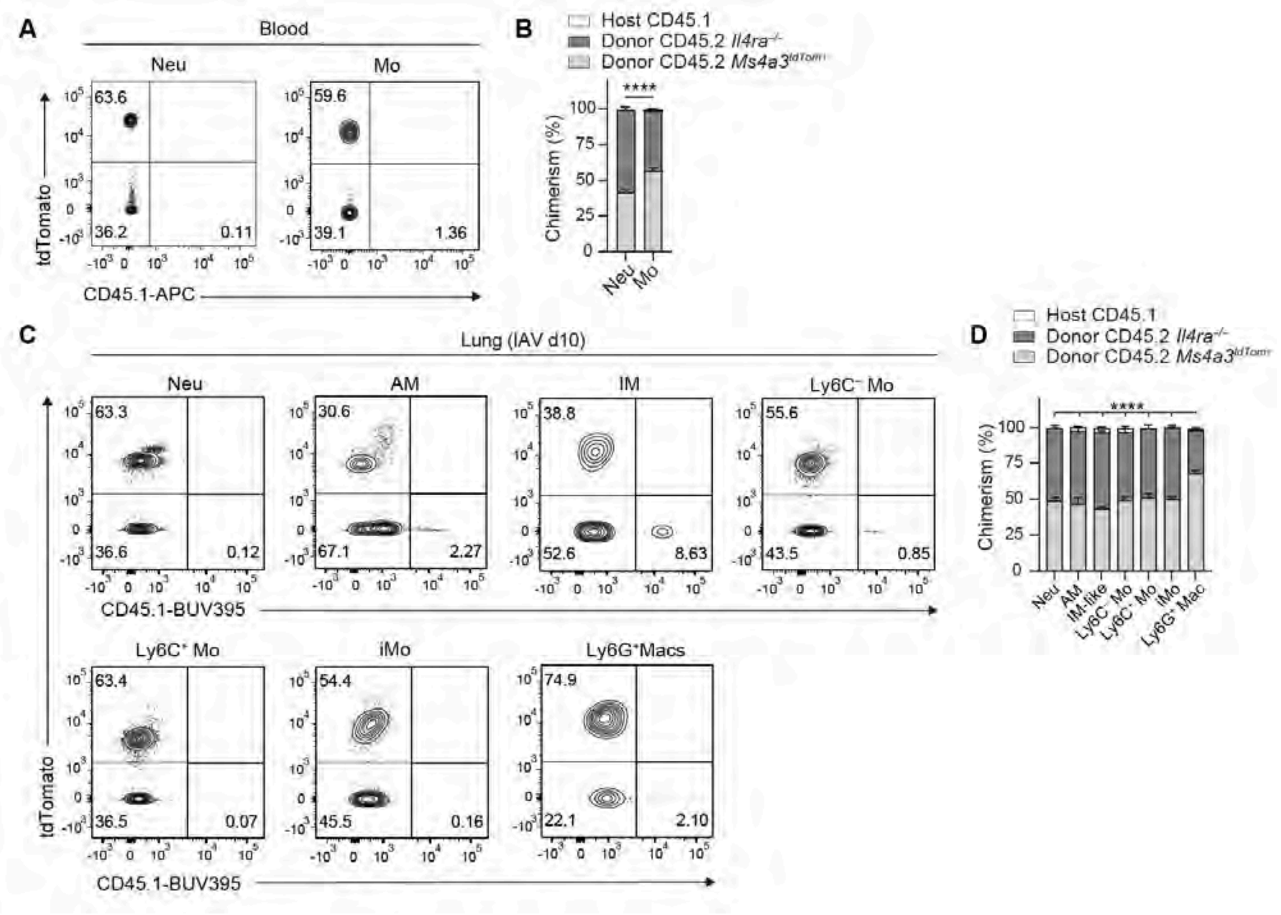

Fig. S10. Analysis of IAV-infected *Il4ra*$^{-/-}$:*Il4ra*$^{+/+}$ mixed BM chimeras. (**A**) Representative tdTomato and CD45.1 contour plots and (**B**) bar graph showing % of host CD45.1, donor CD45.2 *Il4ra*$^{-/-}$ and donor CD45.2 *Ms4a3*$^{tdTom+}$ chimerism of blood neutrophils (Neu) and monocytes (Mo) from lethally-irradiated CD45.1 mice reconstituted with a 1:1 mix of CD45.2 *Il4ra*$^{-/-}$ and *Ms4a3*$^{tdTom+}$ BM cells, evaluated 4 weeks after transplantation by flow cytometry. (**C**) Representative tdTomato and CD45.1 contour plots and (**D**) bar graph showing % of host CD45.1, donor CD45.2 *Il4ra*$^{-/-}$ and *Ms4a3*$^{tdTom+}$ chimerism of the indicated lung myeloid cell populations from lethally-irradiated CD45.1 mice reconstituted with a 1:1 mix of CD45.2 *Il4ra*$^{-/-}$ and *Ms4a3*$^{tdTom+}$ BM cells, infected with IAV 4 weeks later and evaluated at day 10 post-IAV. (B,D) Data show mean + SEM and are pooled from 2 independent experiments (B: *n*=4 mice; D: *n*=8 mice). (B,D) *P* values compare donor CD45.2 *Il4ra*$^{-/-}$ chimerism and were calculated using (B) a two-way ANOVA with Sidak's post hoc tests or (D) a two-way ANOVA with Tukey's post hoc tests. ***, *P*<0.001; ****, *P*<0.0001.

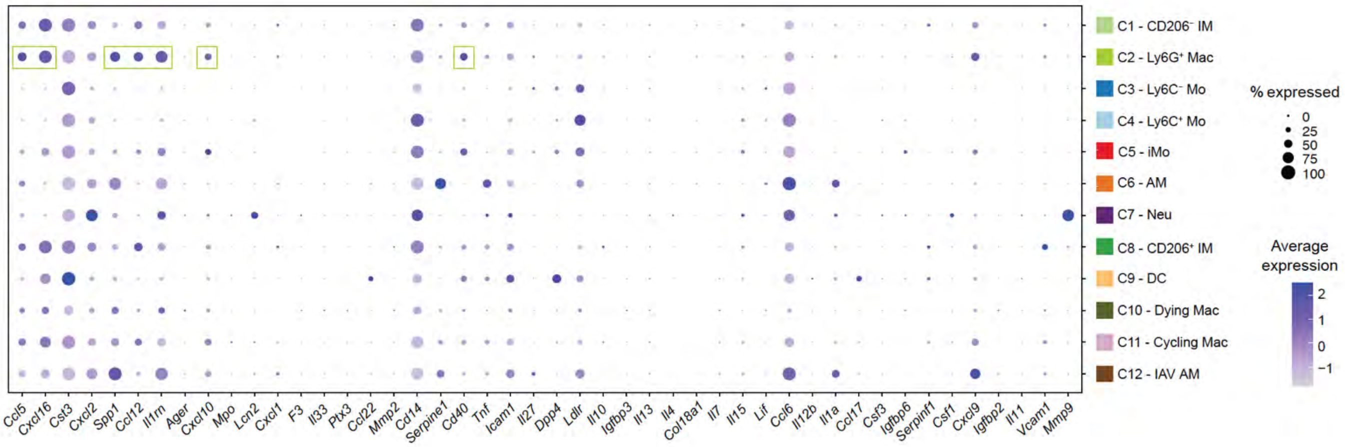

Fig. S11. Gene expression in myeloid cell clusters identified by scRNA-seq at day 10 post-IAV. Dot plots showing average expression of the indicated genes and % of cells expressing the genes within each cluster, related to Fig. 7K and 2A. Dot plots framed in green indicated gene that are significantly (P<0.0001) upregulated in C2 - Ly6G$^+$ Macs as compared to other clusters. *P* values were calculated using a Wilcoxon rank sum test.

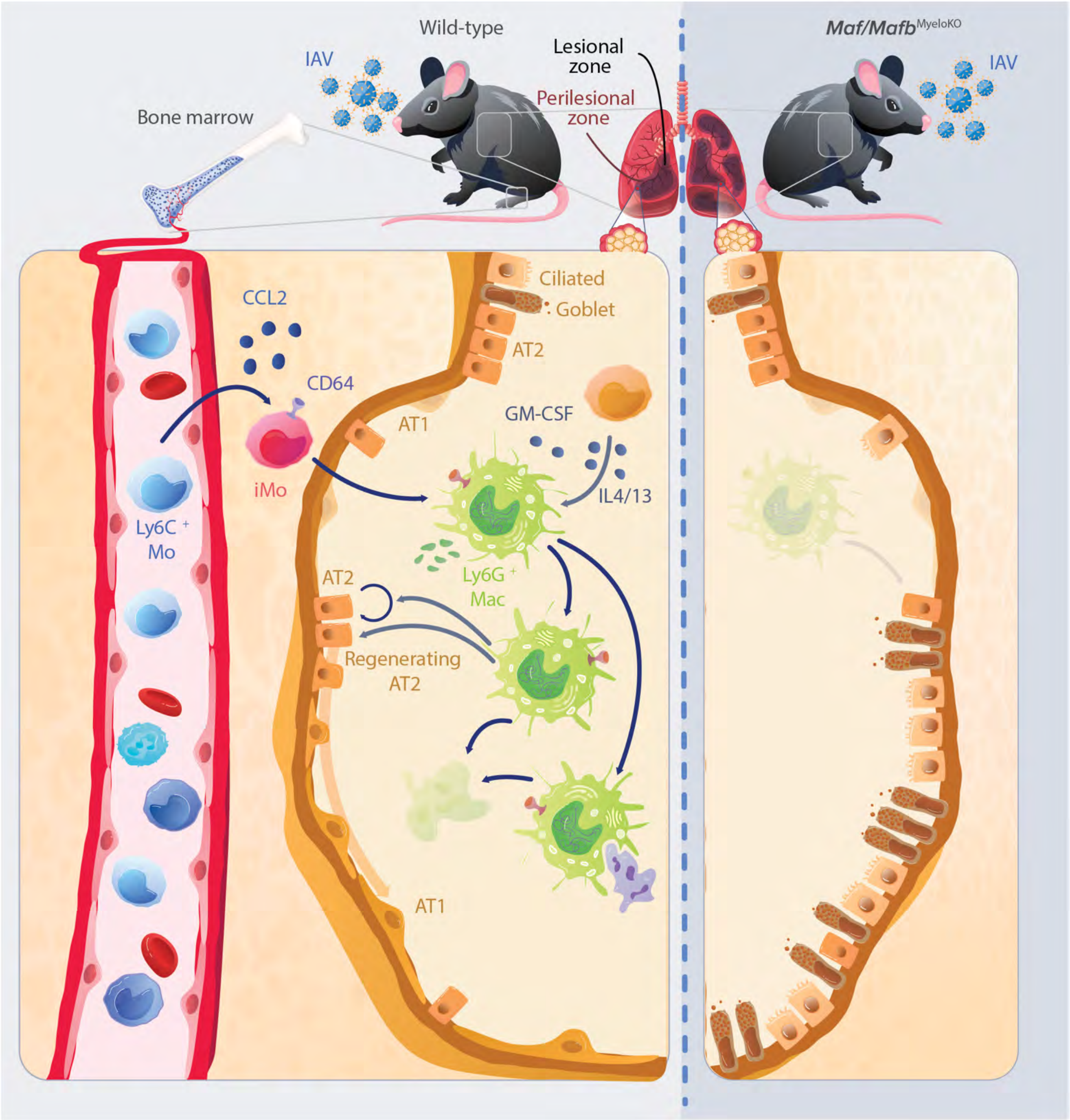
Wild-type
Maf/Mafb^MyeloKO
IAV
Lesional zone
Perilesional zone
Bone marrow
IAV
Ciliated
Goblet
AT2
CCL2
CD64
AT1
GM-CSF
iMo
IL4/13
Ly6C + Mo
AT2
Ly6G + Mac
Regenerating AT2
AT1

Fig. S12. Proposed model of Ly6G$^+$ Mac-mediated alveolar epithelial regeneration after IAV-triggered injury. In mice, an atypical population of Ly6G$^+$ Macs is recruited from BM-derived Ccr2-dependent monocytes during the early recovery phase of IAV infection. Ly6G$^+$ Macs exhibit atypical ultrastructural features, are metabolically very active and short-lived, and are endowed with powerful phagocytic and efferocytic capabilities. They inhabit the alveolar spaces of lung perilesional areas, where they promote euplastic alveolar regeneration and AT2-to-AT1 differentiation via IL-4R-dependent mechanisms and soluble factors. In the absence of Ly6G$^+$ Macs, *Maf/MafB*^{MyeloKO} mice exhibit exacerbated morbidity, pathology and dysplastic bronchiolization of the alveoli.

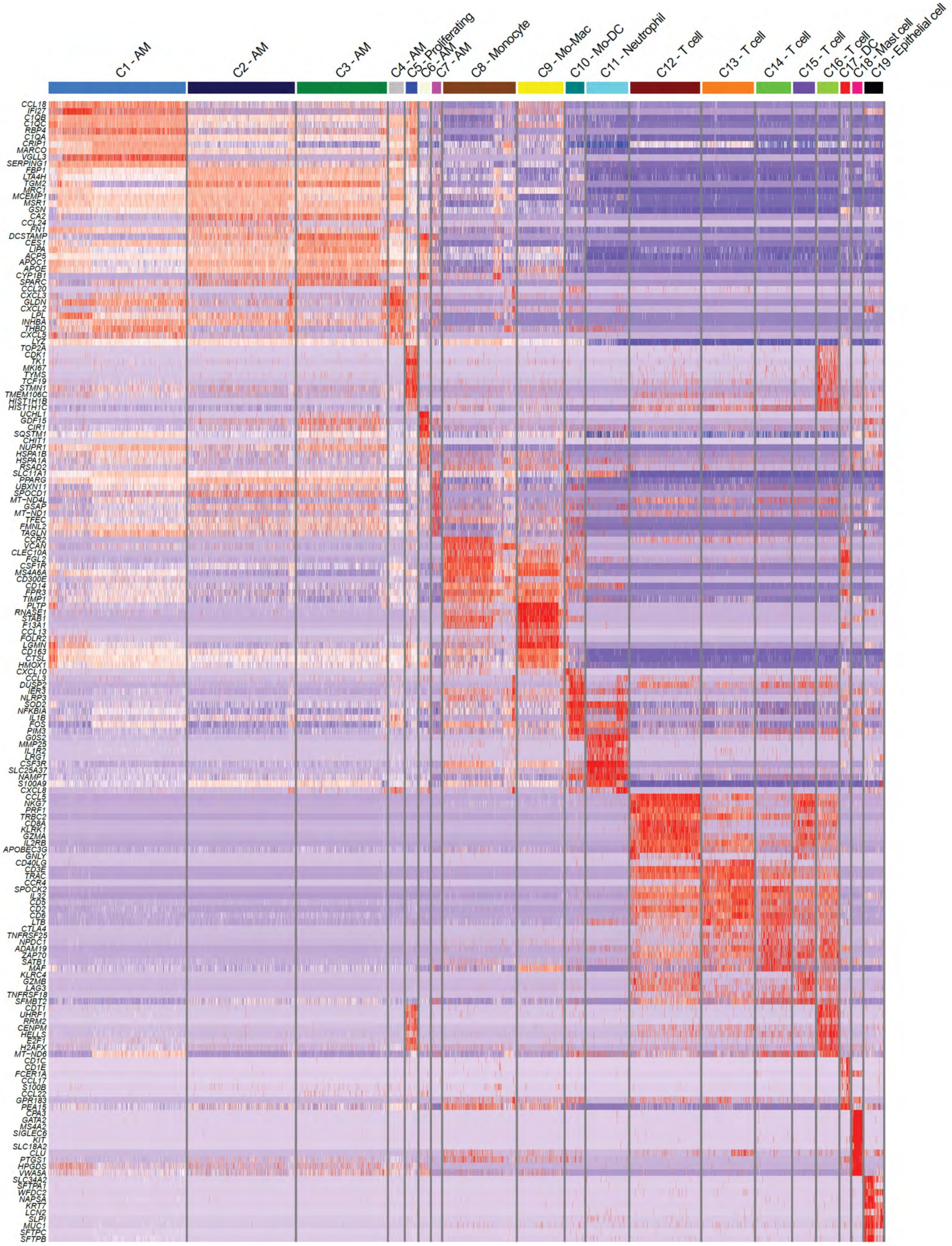

C1 - AM
C2 - AM
C3 - AM
C4 - AM
C5 - Proliferating
C6 - AM
C7 - AM
C8 - Monocyte
C9 - Mo-Mac
C10 - Mo-DC
C11 - Neutrophil
C12 - T cell
C13 - T cell
C14 - T cell
C15 - T cell
C16 - T cell
C17 - DC
C18 - Mast cell
C19 - Epithelial cell
CCL18
IFI27
C1QB
C1QC
RBP4
C1QA
CRIP1
MARCO
VGLL3
SERPING1
FBP1
LTA4H
TGM2
MRC1
MCEMP1
MSR1
GSN
CA2
CCL24
FN1
DCSTAMP
CES1
LIPA
ACP5
APOC1
APOE
CYP1B1
SPARC
CCL20
CXCL3
GLDN
CXCL2
LPL
INHBA
THBD
CXCL5
LYZ
TOP2A
CDK1
TK1
MKI67
TYMS
TCF19
STMN1
TMEM106C
HIST1H1B
HIST1H1C
UCHL1
GDF15
CIR1
SQSTM1
CHIT1
NUPR1
HSPA1B
HSPA1A
RSAD2
SLC11A1
PPARG
UBXN11
SPOCD1
MT−ND4L
GSAP
MT−ND1
TFEC
FMNL2
TAGLN
CCR2
VCAN
CLEC10A
FGL2
CSF1R
MS4A6A
CD300E
CD14
FPR3
TIMP1
PLTP
RNASE1
STAB1
F13A1
CCL13
FOLR2
LGMN
CD163
CTSL
HMOX1
CXCL10
CCL3
DUSP2
IER3
NLRP3
SOD2
NFKBIA
IL1B
FOS
PIM3
G0S2
MMP25
IL1R2
LRG1
CSF3R
SLC25A37
NAMPT
S100A9
CXCL8
CCL5
NKG7
PRF1
TRBC2
CD8A
KLRK1
GZMA
IL2RB
APOBEC3G
GNLY
CD40LG
CD3E
TRAC
CCR4
SPOCK2
IL32
CD5
CD2
CD6
LTB
CTLA4
TNFRSF25
NPDC1
ADAM19
ZAP70
SATB1
MAF
KLRC4
GZMB
LAG3
TNFRSF18
SFMBT2
CDT1
UHRF1
RRM2
CENPM
HELLS
E2F1
H2AFX
MT−ND6
CD1C
CD1E
FCER1A
CCL17
S100B
CCL22
GPR183
PEA15
CPA3
GATA2
MS4A2
SIGLEC6
KIT
SLC18A2
CLU
PTGS1
HPGDS
VWA5A
SLC34A2
SFTPA1
WFDC2
NAPSA
KRT7
LCN2
SLPI
MUC1
SFTPC
SFTPB

Fig. S13. Transcriptomic identities of human BALF single cells analyzed by scRNA-seq. Heatmap depicting the single cell expression of the 20 most upregulated genes within each cluster of human BALF cells analyzed by scRNA-seq, as shown in Fig. 8J.

Patient	Gender	Age	Disease status	Number of BALF cells analyzed
1	**M**	50	Bacterial pneumonia	858
2	**F**	63	Immunosuppressed, bacterial pneumonia	1,032
3	**M**	58	Interstitial pneumonia, metapneumovirus and cytomegalovirus infections	811
4	**M**	60	Pneumonia	1,445
5	**M**	71	Lung fibrosis and bacterial bronchopneumonia	311
6	**F**	62	Pneumonia	289
7	**M**	71	Immunosuppressed, bronchopneumonia	563

Table S1. Characteristics of patients from whom originate the BALF cells analyzed by scRNA-seq.

Reagents	Source	Cat. number
5-Ethynyl-2′-deoxyuridine	Santa Cruz Biotechnology	sc-284628
(Z)-4-Hydroxytamoxifen	Sigma-Aldrich	H7904
10X RBC Lysis Buffer (Multi-species)	Thermo Fisher Scientific	15270658
2-Mercaptoethanol	Thermo Fisher Scientific	21985023
Acetaminophen	Sigma-Aldrich	A7085
ALT ELISA Kit (Mouse)	Abcam	Ab2828-82
Agilent Seahorse XF Base Medium	Agilent	102353-100
Binding Buffer for Annexin V	Thermo Fisher Scientific	BMS500BB
Bleomycin sulfate - 10 mg	BIO-CONNECT	HY-17565
CD11b MicroBeads, human and mouse	Miltenyi Biotec	130-097-142
Cell-Tak™ Cell and Tissue Adhesive, 1 mg	Corning	354240
Click-iT™ Plus EdU Alexa Fluor™ 488 Flow Cytometry Assay Kit	Thermo Fisher Scientific	C10632
Collagenase IV	Thermo Fisher Scientific	17104019
Collagenase A	Roche	10103578001
Dispase® II protease	Sigma-Aldrich	D4693-1G
DMEM/F-12, no phenol red	Thermo Fisher Scientific	21041025
DNase I	Roche	11284932001
Donkey serum	Sigma-Aldrich	D9663
DPBS	Thermo Fisher Scientific	14190094
eBioscience™ Foxp3 / Transcription Factor Staining Buffer Set	Thermo Fisher Scientific	00-5523-00
Elastase from porcine pancreas	MedChemExpress	HY-P2974
Epoxy Embedding Medium kit	Sigma-Aldrich	45359
Fetal Bovine Serum	Thermo Fisher Scientific	A3160801
Formaldehyde, Extra Pure, Solution 37-41%, SLR, Fisher Chemical™	Thermo Fisher Scientific	F/1501/PB15
Hanks Balanced Salt Solution (HBSS)	Lonza	10-508F
Hemacolor	Sigma-Aldrich	111674
HEPES (1 M)	Thermo Fisher Scientific	15630080
Incucyte® Imagelock 96-well Plate	Sartorius	BA-04856
Insulin-Transferrin-Selenium (ITS -G) (100X)	Thermo Fisher Scientific	41400045
iTaq Universal SYBR Green Supermix	BioRad	1725120
L-Glutamine (200 mM)	Thermo Fisher Scientific	25030081
MEM Non-Essential Amino Acids Solution (100X)	Thermo Fisher Scientific	11140050
Méthanol	MerkMillipore	67-56-1
Milieu RPMI 1640	Thermo Fisher Scientific	21875091
Nimatek	Dechra	804132
Nunc® Lab-Tek® Chamber Slide™ system	Sigma-Aldrich	C7182
Penicillin-Streptomycin (10,000 U/mL)	Thermo Fisher Scientific	15140122
pHrodo™ Green E. coli BioParticles™ Conjugate for Phagocytosis	Thermo Fisher Scientific	P35366

Poly-D-lysine hydrobromide	Sigma-Aldrich	P6407
ProLong™ Diamond Antifade Mountant	Thermo Fisher Scientific	P36961
ProLong™ Gold Antifade Mountant with DNA Stain DAPI	Thermo Fisher Scientific	P36931
Propidium iodide	Thermo Fisher Scientific	P1304MP
Proteome Profiler Mouse XL Cytokine Array	R&D	ARY028
Recombinant Murine GM-CSF	Preprotech	315-03
Recombinant Murine IL-13	Preprotech	210-13
Recombinant Murine IL-4	Preprotech	214-14
Recombinant Murine M-CSF	Preprotech	315-02
RevertAid First Strand cDNA Synthesis Kit	Thermo Fisher Scientific	K1621
RNase A	Merck Millipore	70856
Rompun Sol Inj 2%	Bayer	76901
Sucrose	VWR	57-50-1
SYTOX™ Blue Nucleic Acid Stain	Thermo Fisher Scientific	S11348
Tissue-Tek® O.C.T. Compound	VWR	4583
Titriplex® III	Merck Millipore,	1084181000
TRITON® X-100 Detergent	MerkMillipore	648466
Trypsin-EDTA (0.05%), phenol red	Thermo Fisher Scientific	25300062
Tween 20	Acros Organics	AC233360010
UltraPure™ BSA (50 mg/mL)	Thermo Fisher Scientific	AM2616
Zytomed Systems HIER Citrate Buffer pH 6,0 (10 X)	Zytomed	ZUC028-500

Table S2. List of reagents used in this study.

Antibody	Source	Cat. number	RRID	Dilution (1.10^6 cells in 100 µL)
Annexin V APC Conjugate	Thermo Fisher	A35110		
Arginase 1 (A1exF5), APC	Thermo Fisher	17-3697-82	AB_2734835	1/100
Arginase 1 (A1exF5), PerCP-eFluor™ 710	Thermo Fisher	46-3697-82	AB_2734843	1/100
CD101 (Moushi101), PE	Thermo Fisher	12-1011-80	AB_1210729	1/100
CD115 (c-fms) (AFS98), APC	Thermo Fisher	17-1152-82	AB_1210789	1/100
CD11b (M1/70), BUV395	BD Bioscience	563553	AB_2738276	1/200
CD11b (M1/70), APC	Thermo Fisher	17-0112-83	AB_469344	1/100
CD11c (HL3), APC-Cy™7	BD Bioscience	561241	AB_10611727	1/200
CD124/IL4Ra (mILAR-M1), PE	BD Bioscience	561695	AB_10894018	1/100
CD170/Siglec F) (E50-2440), PE	BD Bioscience	552126	AB_394341	1/200
CD170/Siglec F (S17007L)FITC	Biolegend	155503	AB_2750232	1/200
CD177 Alexa Fluor™ 647	BD Bioscience	566599	AB_2869790	1/100
CD184/CXCR4 (2B11), APC	Thermo Fisher	17-9991-82	AB_10670878	1/60
CD31/PECAM-1 (390), APC	Thermo Fisher	17-0311-82	AB_657735	1/100
CD319 (4G2), APC	Biolegend	152003	AB_2632674	1/100
CD326/Epcam (G8.8), BV510	BD Bioscience	747748	AB_2738075	1/100
CD45.1(A20), APC	BD Bioscience	558701	AB_1645214	1/100
CD45.2 (104)V500	BD Bioscience	562129	AB_10897142	1/100
CD45.2 (104), BUV395	BD Bioscience	564616	AB_2738867	1/100
CD45.2 (104), FITC	BD Bioscience	561874	AB_10894189	1/100
CD64 (FcγRI) (X54-5/7.1), Brilliant Violet 421	Biolegend	139309	AB_2562694	1/100
c-MAF (sym0F1), PE	Thermo Fisher	12-9855-42	AB_2572747	1/100
CXCR4 (UMB2), Purified	AbCam	ab124824	AB_2564589	1/100
F4/80 (BM8), Brilliant Violet 650™	Biolegend	123149	AB_11220285	1/100
Ki-67 (SolA15), eFluor™ 570	Thermo Fisher	41-5698-82	AB_11040981	1/600
Ki-67 (SolA15), PerCP-eFluor™ 710	Thermo Fisher	46-5698-82	AB_11040981	0.3
Ly-6C (AL-21), PE-CF594	BD Bioscience	562728	AB_2737749	1/200
Ly-6G (1A8), PE-Cy™7	BD Bioscience	560601	AB_1727562	1/200
Ly-6G (1A8), Purified	BD Bioscience	551459	AB_394206	1/50
MafB (BRL046F), Purified	Bethyl Laboratories Inc.	A700-046		1/100

MHC Class II (I-A/I-E) (M5/114.15.2), Alexa Fluor™ 700	Thermo Fisher	56-5321-82	AB_494009	1/200
MHC II (IA/IE) (M5/114.15.2), PerCP/Cy5.5	Sony	1138130		1/600
Osteopontin (OPN), PE	R&D	IC808P	AB_10643832	1/100
Podoplanin (eBio8.1.1(8.1.1)), Super Bright™ 436	Thermo Fisher	62-5381-82	AB_2744800	1/200
Prosurfactant Protein C (EPR19839), Purified	AbCam	ab211326	AB_2927746	1/200
**Secondary antibodies**				
Goat anti-Rabbit IgG (H+L) Cross-Adsorbed Secondary Antibody, Alexa Fluor™ 532	Invitrogen	A11009	AB_2534076	1/1000
Goat anti-Rabbit IgG (H+L) Highly Cross-Adsorbed Secondary Antibody, Alexa Fluor™ 488	Invitrogen	A21209	AB_2535795	1/1000
GFP Polyclonal Antibody, Alexa Fluor™ 488	Invitrogen	A21311	AB_221477	1/200

Table S3. List of antibodies used in this study.
